# Potential Involvement of the South American Lungfish Intelectin-2 in Innate-Associated Immune Modulation

**DOI:** 10.3390/ijms25094798

**Published:** 2024-04-27

**Authors:** Gabriela Patrícia Martins de Almeida Bernardes, Gustavo Marques Serra, Lucas da Silva e Silva, Maíra Pompeu Martins, Louise Neiva Perez, Fábio Alberto de Molfetta, Agenor Valadares Santos, Maria Paula Cruz Schneider

**Affiliations:** 1 Laboratory of Genomics and Biotechnology, Biological Sciences Institute, Federal University of Pará (UFPA), Belém 66075-110, PA, Brazil; gabrielabiologa@hotmail.com (G.P.M.d.A.B.); silvalucas.ldss@gmail.com (L.d.S.e.S.); mariapaulacruzschneider@gmail.com (M.P.C.S.); 2 Laboratory of Biotechnology of Enzymes and Biotransformation, Biological Sciences Institute, Federal University of Pará (UFPA), Belém 66075-110, PA, Brazil; gustavo.serra@icb.ufpa.br; 3 Department of Biological Sciences, Louisiana State University, Baton Rouge, LA 70803, USA; louiseneivaperez@gmail.com; 4 Laboratory of Molecular Modeling, Exact and Natural Sciences Institute, Federal University of Pará (UFPA), Belém 66075-110, PA, Brazil; fabioam@ufpa.br

**Keywords:** innate immunity, carbohydrate-binding protein, molecular modeling, molecular docking, lectin

## Abstract

Intelectins belong to a family of lectins with specific and transitory carbohydrate interaction capabilities. These interactions are related to the activity of agglutinating pathogens, as intelectins play a significant role in immunity. Despite the prominent immune defense function of intelectins, limited information about its structural characteristics and carbohydrate interaction properties is available. This study investigated an intelectin transcript identified in RNA-seq data obtained from the South American lungfish (*Lepidosiren paradoxa*), namely LpITLN2-B. The structural analyses predicted LpITLN2-B to be a homo-trimeric globular protein with the fibrinogen-like functional domain (FReD), exhibiting a molecular mass of 57 kDa. The quaternary structure is subdivided into three monomers, A, B, and C, and each domain comprises 11 β-sheets: an anti-parallel β-sheet, a β-hairpin, and a disordered β-sheet structure. Molecular docking demonstrates a significant interaction with disaccharides rather than monosaccharides. The preferential interaction with disaccharides highlights the potential interaction with pathogen molecules, such as LPS and Poly(I:C). The hemagglutination assay inhibited lectins activity, especially maltose and sucrose, highlighting lectin activity in *L. paradoxa* samples. Overall, our results show the potential relevance of LpITLN2-B in *L. paradoxa* immune defense against pathogens.

## 1. Introduction

The lungfish *Lepidosiren paradoxa* is the only South American Dipnoi, part of the Sarcopterygii group, the closest-related living relatives of tetrapods. It has an elongated, eel-like body, a flat head, two pairs of lobed fins (pectoral and pelvic), and it can reach about 1.5 m in length [1]. In Brazil, the South American lungfish is mainly found in flooded areas, swamps, and lakes with low oxygen content in the Amazon region, remaining occasionally in burrows excavated in the mud [2,3].

Lungfish skin acts as a major physical defense barrier, supported by a mucus layer that wields a primary immunological barrier role, acting as a defensive line against a plethora of pathogens [3]. Both skin and mucus exert physical, chemical, and biological barrier activities, playing different roles in adaptation, maintenance, and protection, including sealing moisture, participating in respiration, and trapping and immobilizing pathogens before penetrating epithelial surfaces [4,5]. The lungfish skin-associated immune role was evaluated in *Protopterus dolloi* (the African lungfish) using its skin mucus. This study discovered humoral immunity molecules [6]. While the *L. paradoxa* epidermis is considered a key component of its immune defense, its molecular mechanisms remain unclear.

Lectins are proteins that are capable of binding cells through their specific and transient interaction with carbohydrates [7]. Immune-related lectins play a significant role in both innate and adaptive immunity. In the former, these molecules may function as pattern recognition receptors (PRRs), identifying foreign carbohydrate molecules as non-self and inducing opsonization and complement activation, phagocytosis, and lymphocyte proliferation [8,9].

In fishes, studies have demonstrated that skin mucus-associated lectins possess agglutination activity against bacteria [10,11], fungi [12], and parasites [9]. These macromolecules exhibit high specificity toward their ligands and can prevent potential pathogens from adhering to the fish body, thus inhibiting their invasion. Due to these properties, lectins are considered potential antimicrobial agents in mucus [9].

Intelectins are a family of Ca^2+^-dependent lectins that contain a conserved fibrinogen-like domain (FReD) [13]. Highly conserved across chordate evolution, these secreted lectins can agglutinate microorganisms, suggesting their role in innate immunity [14,15]. These carbohydrate-binding proteins are also referred to as X-lectins, since the first intelectin was reported in *Xenopus laevis* oocytes [16]. At least five *X. laevis* intelectins have been characterized for their structure and function: the cortical granule lectins (XCGL-1 and XCGL-1) that aid in the development of embryos [17] and oocytes [18]; lectins (XSL-1 and XSL-2) that are transcriptionally activated when exposed to lipopolysaccharide (LPS) [19]; and epidermal lectin (XEEL), released during development [20].

Intelectins were described in fishes, such as catfish (*Silurus asotus*) [21], goldfish (*Carassius auratus*) [22], and zebrafish (*Danio rerio*) [23]. The role of intelectins in fishes’ innate immune response was observed through agglutination activity due to their carbohydrate-binding capacity. In zebrafish, activity was observed against both Gram-positive and Gram-negative bacteria [23,24]. Its role in self-defense against bacteria was also observed in catfish [21], as confirmed through agglutination tests against *Aeromonas salmonicida*. *Megalobrama amblycephala* [25] was also studied to show the immune traits of intelectins, highlighting their relevance in pathogen defense.

In the present study, we assessed *L. paradoxa* transcriptomes to retrieve lectin transcripts. We explored in silico the Intelectin-2 LpITLN2-B, investigating its structural properties through protein folding analysis, putative recognition, and interaction with bacterial wall components (LPS) and viral nucleic acids (Poly(I:C)) exploring properties associated with potential innate immune function in *L. paradoxa*. Additionally, a hemagglutination assay with *L. paradoxa* mucus revealed lectin sugar-binding activity. Similarly to our in silico data that showed that intelectin LpITLN2-B has disaccharide affinity, the hemagglutination assay inhibited lectins activity, especially with maltose and sucrose. The inhibition of lectin-induced agglutination highlights its carbohydrate recognition properties.

## 2. Results

### 2.1. Lectin Identification and GO Classification

**Figure 1:**
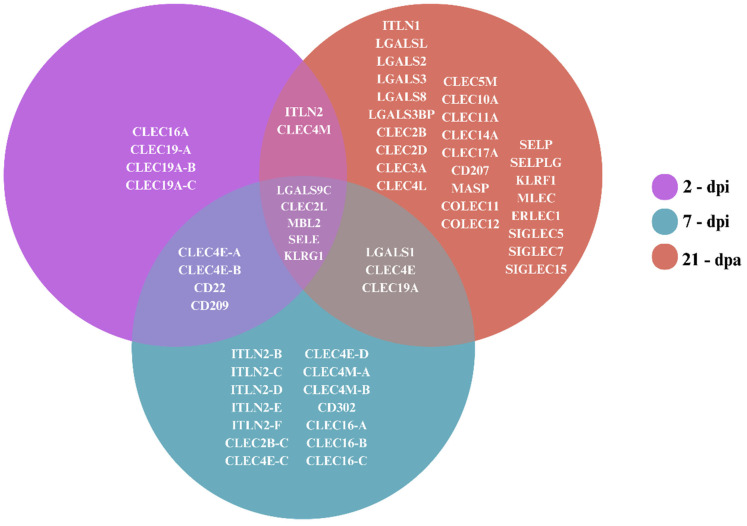
Venn diagram of *Lepidosiren paradoxa* lectins identified at 21 days post-amputation and 2 and 7 days post-skin injury through transcriptomes.

We searched for lectin in three *L. paradoxa* transcriptomes generated 2 and 7 days post-skin injury (dpi) and 21 days post-amputation (dpa) [26]. We identified 59 lectin genes: 4 were exclusive to the 2 dpi library; 14 were exclusive to 7 dpi, and 27 were exclusively identified in the 21 dpa transcriptome (Figure 1). The three evaluated time points shared 5 lectins.

**Figure 2:**
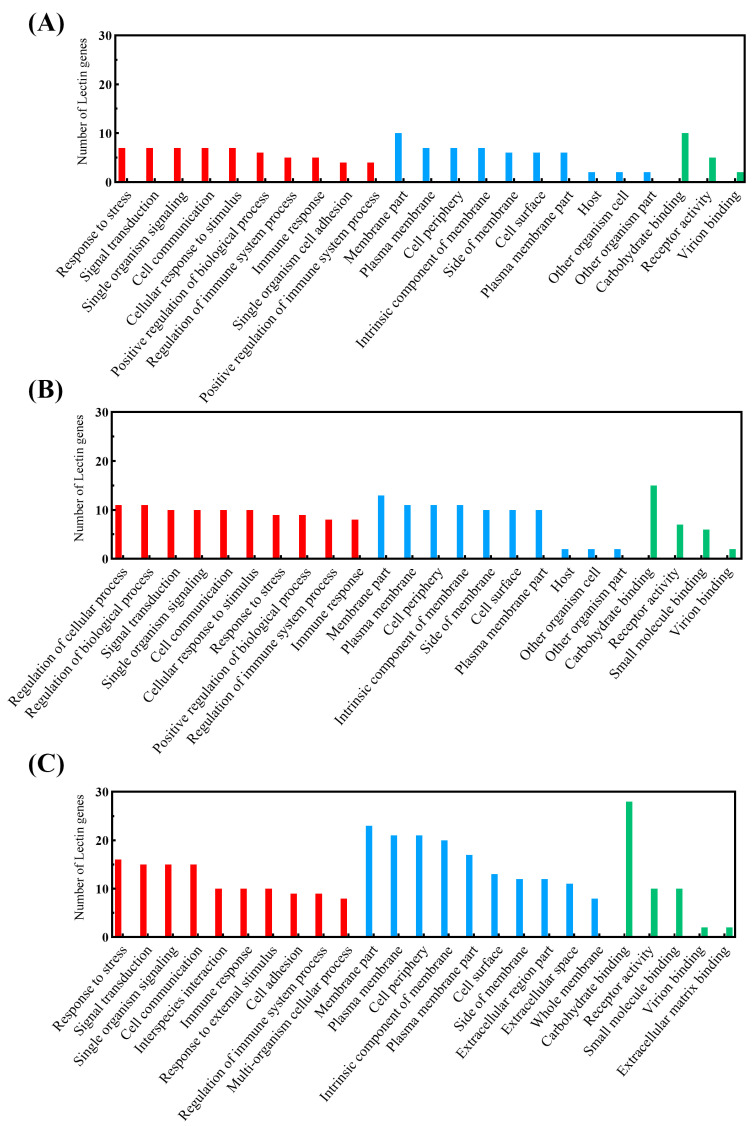
Gene ontology of the identified lectins at (**A**) 2 days post-skin injury (dpi), (**B**) 7 dpi, and (**C**) 21 days post-amputation (dpa). Colors are as follows: biological processes (red), cellular component (blue), molecular function (green).

Gene ontology enrichment showed the association of the lectins present at 2 dpi with the GO terms stress response, signal transduction, single organism signaling, cell communication, and cellular response to stimuli in the biological process (BP) category (Figure 2A). In the 7 dpi transcriptome (Figure 2B), the most evident GO terms were the regulation of cellular processes and biological regulation. The enrichment of the 21 dpa transcriptome (Figure 2C) showed stress response as the most evident term, followed by signal transduction, single-organism signaling, and cell communication. At all time points, proteins were associated with the immune response term.

Among the lectin transcripts identified in RNA-seq results, we selected the members of the intelectin family for further evaluation. Five intelectins, ITLN2-B, -C, -D, -E, and –F, exclusive to the 7 days post-skin injury (dpi) library, were considered as potential subjects to study their properties against different ligands. An extensive search through the PDB database determined that ITLN2-B presents the greatest accuracy among the five evaluated proteins, as it has the lowest RMSD (0.078 Å) and structural resolution (1.59 Å) values, with satisfactory values for structural and interactions-with-ligands studies.

### 2.2. Sequence Analysis of LpITLN2-B

**Figure 3:**
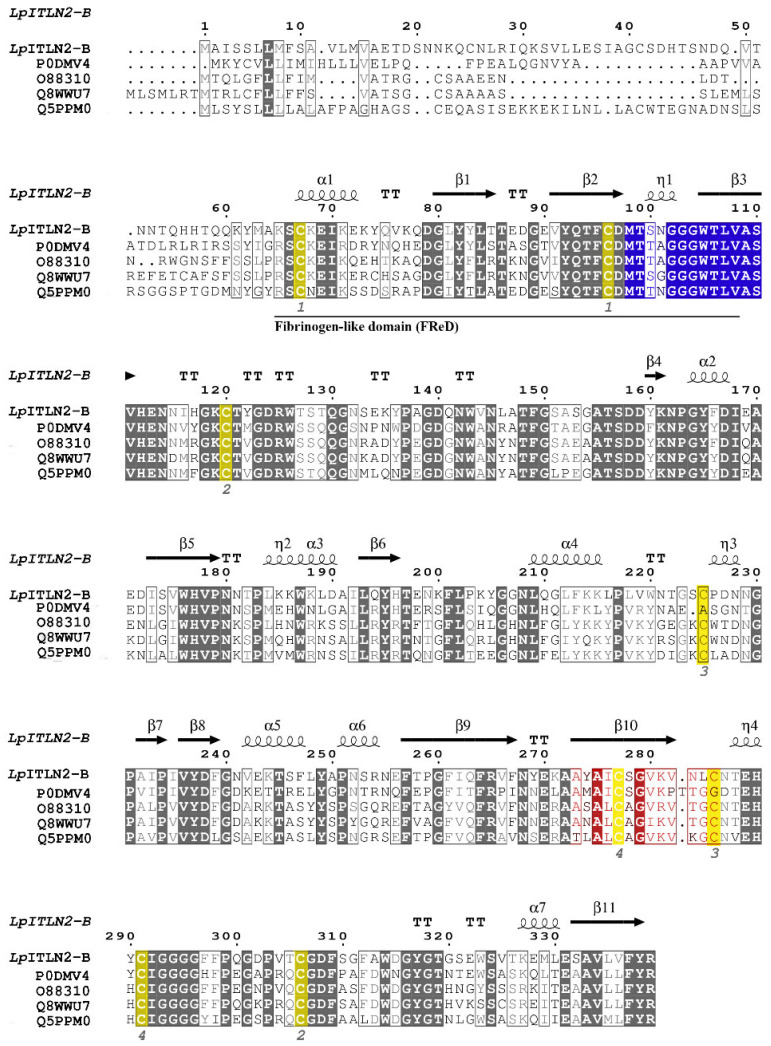
Multiple alignment of the amino acid sequence of *Lepidosiren paradoxa* intelectin-2-B with *O. mykiss* (UniProt Accession P0DMV4), *M. musculus* (UniProt Accession O88310), *H. sapiens* (UniProt Accession Q8WWU7), and *X. laevis* (UniProt Accession Q5PPM0) intelectins. Colors are as follows: cysteine residues (yellow), carbohydrate-binding site (blue), calcium-binding site and carbohydrate binding site (red), identical residues (grey), partially conserved amino acid residues (light grey). Symbols are as follows: helices (ŋ), α-strands (α), β-turns (TT).

To identify potential functional regions in the intelectin gene family, we analyzed primary structures through alignments of multiple intelectin sequences, and functional domains/motifs were predicted using SMART and Clustal Omega (Figure 3). The comparative analysis revealed similarity between the LpITLN2-B amino acid (aa) sequence and other intelectins, as follows: 57.06% identity (ID) to Intelectin-1 from *Oncorhynchus mykiss*, 54.29% ID to Intelectin from *Mus musculus*, 53.50% ID to Intelectin-2 from *Homo sapiens*, and 51.92% ID to Intelectin-1 from *Xenopus laevis*.

The comparative analysis between the LpITLN2-B amino acid sequence and the four orthologs showed that the most notable distinguishing features are at the N-terminus region. The conserved fibrinogen-like domain (FReD) comprises the amino acids Lys65-Val108 in the N-terminal region, such as those found in lamprey’s ITLN-homolog LSSL (40–50 amino acids), and it contains a predicted carbohydrate-binding site according to SMART results. Typically, these intelectins have FReD domains of approximately 200 amino acids [15]. The FReD domain found in lamprey’s LSSL possesses a high homology to human ITLN1 and ITLN2 and is described as a more efficient system for triggering an immune response [27]. COACH results demonstrated that the predicted LpITLN2-B binding site is composed of consensus residues 191, 195, 207, 208, 209, 213, 228, 229, 244, 269, 270, 286, and 289, with a confidence score (C-score) of 0.44. C-score values range between 0 and 1, where a higher score indicates a more reliable prediction. All regions predicted as carbohydrate-binding sites by COACH were used for subsequent molecular docking assays.

Moreover, intelectin protein sequences were highly conserved in some regions, including conserved residues of cysteine in positions 67, 96, 120, 225, 277, 285, 291, and 306, as observed in mouse and human ITLN1. Our results also showed that LpITLN2-B lacks one (Cys31) of the two Cys residues (Cys31 and Cys48) present in human ITLN1, responsible for stabilizing the disulfide-linked trimer. In addition to the Cys48 residue (position 41, Figure 3), LpITLN2-B presents with a Cys25 residue three amino acids away from the human Cys31 (position 22, Figure 3) [28].

Typically, intelectins present a calcium ion (Ca^2+^)-binding site. In LpITLN2-B, the conserved calcium-binding residues were identified in the 273–285 aa region. The estimated molecular weight of LpITLN2-B is 37.7 kDa. Intelectins of this family have been shown to exist as homo-oligomers of 35 to 40 kDa monomers [13,29]. The theoretical pI of LpITLN2-B was 5.39, a value similar to that observed for the human Intelectin-1 (hIntL-1) of approximately 5.5. As suggested for the human intelectin, the interaction may be mediated by bulk Coulombian interactions [29]. The instability index was computed to be 34.81, meaning this protein is predicted to best able under assay conditions. The total mean value of hydrophilicity (GRAVY) was −0.46. The total number of negatively charged (Asp + Glu) and positively charged (Arg + Lys) residues was 36 and 27, respectively.

### 2.3. Validation of Predicted Structure

To understand the structure and function of LpITLN2-B, we predicted its three-dimensional (3D) structure. The template chosen was the human protein (PDB ID) 6USC chain A [30]. Our results revealed 58.12% identity and an E-value of 7 × 10^−130^. The template structure solved by X-ray diffraction has a resolution of 1.59 Å and presented 279 aa residues.

**Figure 4:**
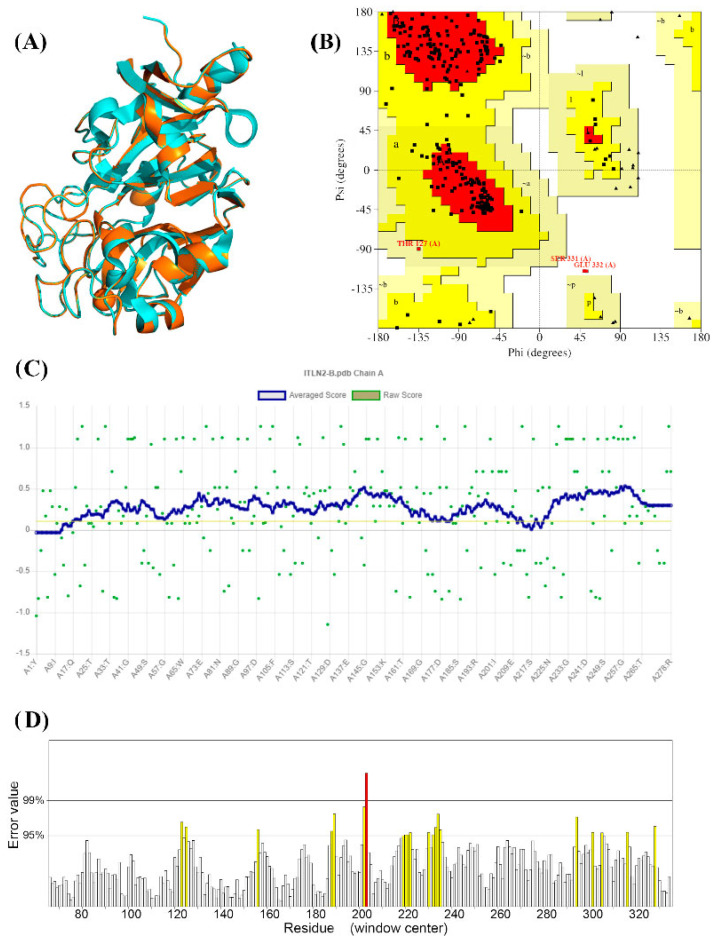
Model quality assessment and validation. (**A**) Superposition of the generated model and the template (PDB ID: 6USC). (**B**) Ramachandran plot by PROCHECK. (**C**) Verify3D graphic result and (**D**) ERRAT graphic result.

LpITLN2-B presented with an RMSD of 0.079 Å (Figure 4A), demonstrating that the superposition of the LpITLN2-B protein and the 6USC chain A template are closely related. The Ramachandran plot (Figure 4B) showed that 83.30% of the residues are in energetically favorable positions in the 3D structure, and Verify3D (Figure 4C) showed that 90.29% of the residues are in acceptable positions, with a mean score of 0.1. The overall quality of the homology protein model was 92.20% in the ERRAT plot (Figure 4D).

### 2.4. Structure of LpITLN2-B

**Figure 5:**
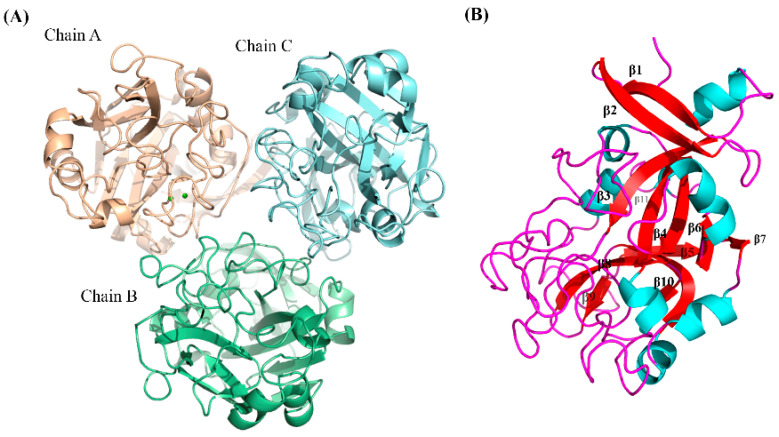
(**A**) Overall three-dimensional structure of LpITLN2-B. (**B**) Ribbon diagram of LpITLN2-Bstructure (β-strands, β1–11). Colors are as follows: (**A**) Chain A is colored in light pink, Chain B is in Green, Chain C is colored in lime green; (**B**) β-sheets are in red, α-helix in cyan, and loops are in pink.

Our results indicate that LpITLN2-B is predicted to be a homo-trimeric protein. We have established that the quaternary structure is subdivided into three monomers, A, B, and C (Figure 5A). The C-terminal of each of the monomeric sequences is highly conserved, and all three chains have globular folding. The N-terminal regions are not associated with the interaction among the three domains. The LpITLN2-B structure has weak interaction, with non-covalent bonds such as van der Waals, hydrogen bonds, and electrostatic interactions.

Each domain contains 11 β-sheets, an anti-parallel β-sheet-like group (β7–β10), a β-hairpin (β1–β2), and a highly disordered structure comprising β3–β6 (Figure 5B). The position and length of the model secondary structure match the template (Figure 4A). We observed the formation of disulfide bonds in positions Cys67–Cys96; Cys120–Cys306; Cys225–Cys285; and Cys277–Cys291. The LpITLN2-B binding pocket in the chain A monomer is solvent-exposed (Figure 5A).

**Figure 6:**
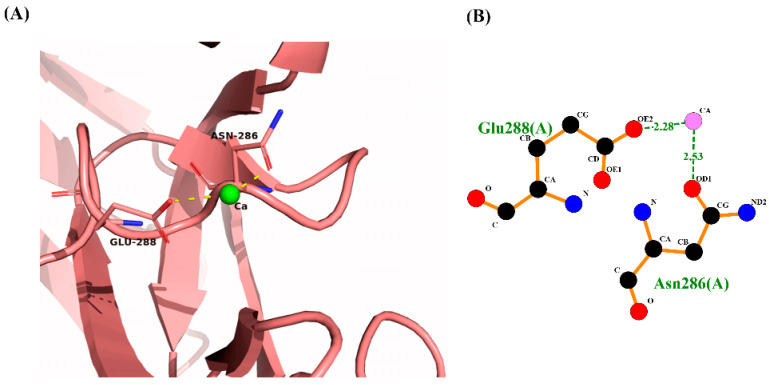
(**A**) 3D interaction of Ca^2+^ ion with binding site residues. (**B**) 2D representation, with the distance of hydrogen bonds expressed in Å.

Furthermore, a Ca^2+^ ion is present in one of the monomers (chain A), which is in the carbohydrate-binding pocket template, and two residues bind to the Ca^2+^ ion: one uncharged residue Asn286 and one charged residue Glu288 (Figure 6).

The LpITLN2-B and human ITLN1 (hITLN1) structures are comparable. The latter is a trimeric structure consisting of two highly distorted β-sheets surrounded by seven short α-helices and random coiled-coil regions [30]. Similar structures have been observed in the lamprey *Lethenteron camtschaticum* (PDB: 7E1E) and epidermal *X. laevis*, XEEL (PDB: 5ZC0) intelectins.

### 2.5. Molecular Docking

The best ligand conformations with the lowest free energy (kcal/mol), were determined based on the GoldScore function for GOLD and DockThor (Table 1). DockThor and GOLD results demonstrate LpITLN2-B’s capability to bind a range of carbohydrates. The results showed predicted high affinity to maltose, with GoldScore values of 72.46 and –7.69 kcal/mol of binding free energy, and also the Ca^2+^-dependent binding to other glycans such as lactose, mannose and *N*-acetylglucosamine. The interactions between docked ligands and catalytic site residues using GOLD and DockThor are summarized in Supplementary Tables S1 and S2, respectively.

#### 2.5.1. LpITLN2-B/Maltose Complex

To determine how LpITLN2-B binds its ligand, we determined the structure of the LpITLN2-B/maltose complex based on the highest values for specificity and, consequently, the best chance of identifying the majority of residues for binding to carbohydrates. LpITLN2-B/maltose showed good results with values of the GoldScore and lowest binding free energy of 72.46 and −7.69 kcal/mol, respectively (Table 1).

**Figure 7:**
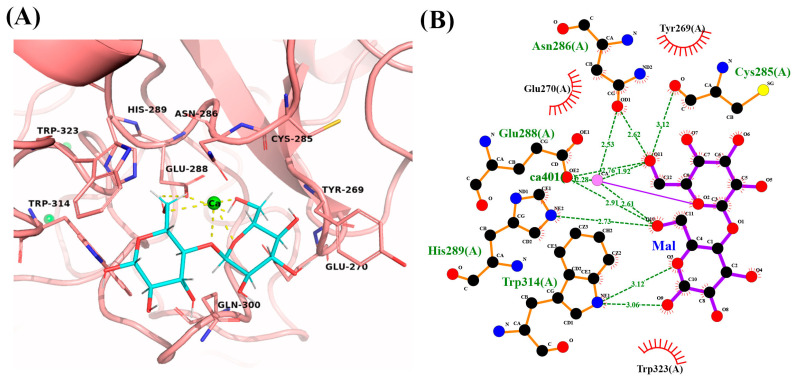
(**A**) Carbohydrate-binding site of LpITLN2-B forming a complex with maltose (cyan sticks). Yellow dashes represent polar contacts. (**B**) 2D representation of hydrogen bonds and hydrophobic interactions around maltose.

The LpITLN2-B/maltose complex interface was further analyzed to identify residues within 3.5 Å in the interactions (Figure 7A and Supplementary Table S1). The identified residues Cys285, Asn286, Glu288, His289, and Trp314 formed bonds to oxygen atoms O3, O9, O10, and O11 of the maltose molecule, contributing to the interaction between the carbohydrate and the intelectin. Hydrophobic interactions involving the amino acid residues Tyr269, Glu270, Gln300, and Trp323 also contribute to intelectin/disaccharide binding (Figure 7B).

#### 2.5.2. LpITLN2-B/Poly(I:C) Complex

**Figure 8:**
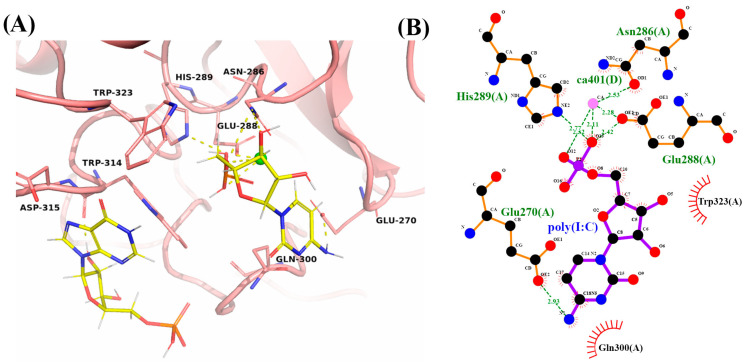
(**A**) Carbohydrate-binding site of LpITLN2-B with complex to poly(I:C) in yellow sticks. Yellow dashes represent polar contacts. (**B**) 2D representation of hydrogen bonds and hydrophobic interactions around poly(I:C).

Potential LpITLN2-B/Poly(I:C) complex formation was evaluated through GOLD and DockThor programs and resulted in a score of 64.91 in GoldScore function and −6.68 kcal/mol of binding free energy, respectively (Table 1). The interaction analysis of the LpITLN2-B/Poly(I:C) complex by GOLD is in Figure 8. The analysis showed interaction with Glu270, Asn286, Glu288, and His289 residues, with a distance smaller than 3.0 Å among residues and molecule atoms. Such distances contribute to a high probability of interaction (Figure 8B).

#### 2.5.3. LpITLN2-B/LPS Complex

**Figure 9:**
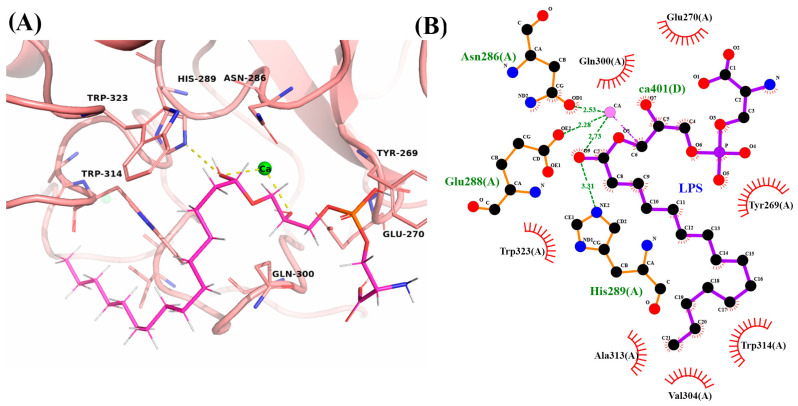
(**A**) Carbohydrate-binding site of LpITLN2-B forming a complex with LPS, represented by pink sticks. Yellow dashes represent polar contacts. (**B**) 2D representation of hydrogen bonds and hydrophobic interactions around LPS.

The selected DockThor and GOLD LpITLN2-B/LPS complex showed a GoldScore function of 61.03 and a binding free energy of –6.19 kcal/mol. Only a few interactions were observed in the LpITLN2-B/LPS complex (Figure 9 and Supplementary Table S1), resulting in longer-distance hydrogen bonds formed and a lower affinity value to the molecule in the DockThor program compared to other carbohydrates analyzed (Supplementary Table S2). Both programs corroborate their results from their analyses and suggest the potential interaction between intelectin and lipopolysaccharide. H-bonds are considered facilitators of protein–ligand binding [31], and the further analysis of the decomposition of binding free energy could contribute to clarifying these results.

### 2.6. Carbohydrate Inhibition Assay

The carbohydrate specificity profile of *L. paradoxa* lectins was tested for the agglutination of rabbit erythrocytes using 2 dpi and 7 dpi samples. Our results showed that most carbohydrates inhibited lectin activity, especially the disaccharides maltose and sucrose (Table 2). In the presence of 100 mM lactose, an interaction was observed in the 2 dpi sample, while lactose at 100 mM in the 7 dpi sample and at 200 mM in both 2 dpi and 7 dpi samples resulted in inhibited activity. Fructose showed a strong interaction at 100 mM in 2 dpi and 7 dpi samples and at 200 mM in the 2 dpi sample, with inhibition observed at 7 dpi at 200 mM of fructose.

## 3. Discussion

Lungfish have the ability to breathe air though the lungs. This trait can therefore evolutionarily be traced to the same origin of land-dwelling vertebrates, including humans [32]. The phylogenetic relationships among lungfish and other Sarcopterygii can be shown through highly similar protein sequences, as observed with intelectins. Intelectins are a unique group of carbohydrate-binding proteins or glycoproteins which possess specific and reversible carbohydrate interaction capability through at least one carbohydrate recognition domain (CRD). These proteins are widely distributed in vertebrates and invertebrates and act by activating the innate immune system through pathogen recognition, such as the specific recognition of carbohydrate epitopes on microbial surfaces [29]. Additionally, they are associated with other relevant functions such as pathogen agglutination and opsonization [33]. Intelectins present orthologs in species ranging from placozoans to vertebrates, including fishes (i.e., zebrafish, goldfish, common carp) [15,33]. To date, the structural and carbohydrate affinity contribution of South American lungfish intelectins has not been elucidated.

The analysis of three transcriptomic libraries revealed a diversified repertoire of lectins in the South American lungfish *Lepidosiren paradoxa*, including the C-type, galectin, and intelectin families. While at 2 dpi and 21 dpa, more C-type lectins were identified, at 7 dpi, a high number of intelectin transcripts were observed. The GO enrichment results suggest an attempt to restore damaged tissue through the modulation of lectins associated with cellular stress response activity, signal transduction, cellular communication, and immune response. At the three time points, we identified proteins related to virion binding, and at the two later time points, proteins were associated with the term small molecule binding. Lungfish can regenerate paired appendages through the induction of oncogenes and developmental genes [26]. Our results suggest that intelectins can participate in regenerative and healing processes, being recruited especially in the latter period post skin injury.

Comparative homology modeling has shown that intelectins are structurally diverse in different species, even though they have highly conserved sequences. LpITLN2-B is predicted to be a globular protein with three identical subunits and structural similarity to human Intelectin-1 (hItln-1) [29]. This similarity between lungfish intelectin and humans, in contrast to other fish, can be understood from the evolutionary link between both species. The A-chain binding pocket is solvent-exposed and is responsible for the protein–carbohydrate interaction, as found in other intelectins [29,34,35]. This role is reinforced by the presence of the FReD domain, which is homologous to hItln-1 and XEEL, with at least 50% identity to these proteins. These lectins present increased affinity to pentoses and galactofuranose [20,35], and these binding site regions are of paramount importance.

The oligomeric states linked by disulfide bonds of the intelectins in different species differ. In most mammalian intelectins, the intelectin genes contain 10 conserved cysteine residues [29,33,34,35,36,37]. Thus, in LpITLN2-B, it was possible to identify 8 cysteine residues responsible for the intermolecular formation of disulfide bonds and, therefore, protein structural stabilization [29,34,37]. Wesener and collaborators [29] support this hypothesis, since disulfide bonds are responsible for the integrity of structures relating to recognition and binding to carbohydrates, as well as their release. Furthermore, cysteine residues are involved in intermolecular trimerization and are important in forming monomers.

The binding pocket of lungfish intelectin is composed of cysteine, asparagine, glutamate, histidine, tyrosine, and tryptophan amino acid residues, facilitating hydrogen bonds and hydrophobic interactions that are vital for effective docking between proteins and carbohydrates. This functionality is primarily attributed to the presence of acid/base residues. We anticipate that this binding provides information on the interaction role of the host and the infectious agent and may not be directly related to the immune response but rather to the recognition and interaction of the presence of these agents. Our results showed that LpITLN2-B has an affinity mainly for disaccharides over monosaccharides, as observed for the amino saccharide *N*-acetylglucosamine (GlcNAc), a pathogen-associated molecular pattern (PAMP). These PAMPs are important in innate immune response, allowing the immune system to detect and respond to infections and pathogen invasions [33]. Conversely, *X. laevis* intelectin XEEL evidenced higher affinity to pentoses than disaccharides [28].

Furthermore, our results show a high interaction probability for LpITLN2-B and poly(I:C). The poly(I:C) molecule is commonly used to mimic viral dsRNA, suggesting potential antiviral-linked LpITLN2-B activity. Intelectins are described as having a complex interaction with viral molecules that depend on different factors to induce an efficient immune response; however, these molecules may have the ability to attenuate the immune response to viral molecules through direct interaction [36].

Lectins, including intelectins, are ubiquitous proteins that are able to bind to specific kinds of saccharide compounds. Since carbohydrates are universally present on the cell surface as a part of glycolipids and glycoproteins, lectin activity can help determine the specific saccharide for the lectin of interest, suggesting the membrane’s composition [38]. Our in silico molecular docking results predicted a potential LpITLN2-B/maltose complex formation, and the hemagglutination assay confirmed the potential activity of this intelectin, especially with the oligosaccharides maltose and sucrose, indicating a correlation between our in silico results and the *L. paradoxa* immune system.

Conclusively, the molecular configuration of LpITLN2-B illustrates an intricate and functionally significant architecture, underscoring its capacity to engage with microbial glycans, potentially exhibiting selective recognition for the detection or targeting of infectious agents for eradication. Given the ubiquitous presence of carbohydrates on cell surfaces as constituents of glycolipids and glycoproteins, lectin activity aids in discerning the specific saccharide targeted by the pertinent lectin, thereby indicating membrane composition [37]. Therefore, the study offers valuable insights into the structure and crucial interactions underlying the immune response of the South American lungfish.

## 4. Materials and Methods

### 4.1. Animals

A total of 3 adult *Lepidosiren paradoxa* specimens with an average weight of 7.2 kg were obtained from natural sources in the State of Pará, Brazil (SISBIO license n° 79802-2). Animals were kept in individual tanks with regular water changes. They were fed daily with a ration (the amount corresponding to 36% of the fish’s body weight) for ten days for laboratory acclimation. Animals were anesthetized in 0.1% clove oil diluted in water, and 6 mm biopsy punches were obtained from each animal. Three more samples were collected per animal 2 days and 7 days post-initial skin injury after anesthetization. Each of the samples was processed individually. All collected skin samples were maintained in a −80 °C freezer until RNA extraction. Animal care was conducted following the guidelines of the Animal Research Ethics Committee of the Federal University of Pará under protocol CEUA N° 8251300622. The 21 days post-amputation results are registered under Bioproject PRJNA301439 in the NCBI database [26].

### 4.2. RNA Extraction, Sequencing and Bioinformatic Analysis

Three *L. paradoxa* transcriptome datasets were analyzed: 2 and 7 days post-skin injury (dpi) and 21 days post-amputation (dpa) [26]. The RNA isolation of 2 and 7 days post-injury samples was carried out using TRIzol (Sigma-Aldrich, St. Louis, MO, USA) according to the manufacturer’s instructions. The quality, concentration, and integrity of RNA samples were determined by a NanoDrop™ (Thermo Fisher, Waltham, MA, USA) spectrophotometer and 1% agarose gel electrophoresis.

The RNA samples were sequenced at Novogene Co. (Sacramento, CA, USA). Libraries were generated (poly-A enrichment) and sequenced on an Illumina^®^ Novaseq platform (Illumina, San Diego, CA, USA) with 150 bp paired-end reads (NCBI Sequence Read Archive project number PRJNA1001397). We used FastQC v0.11.9 to check the average reads quality and Trimmomatic v0.40 [39] to retrieve the high-quality reads. We performed two de novo assemblies to obtain gene transcripts: a reference transcriptome using all the libraries and assemblies per condition (2 and 7 dpi), both using Trinity v2.15.0 [40]. The assemblies quality was checked with assembly stats v1.0.1 and BUSCO v3 [41]. The reference transcriptome transcripts were annotated with BLASTx (BLAST+ 2.13.0), and bowtie2 v2.5.1 was used to perform mapping against the assemblies per condition and, thus, identify lectins genes (Supplementary Material S1). One intelectin identified as Intelectin-2 variant B (LpITLN2-B) was retrieved from the 7 dpi transcriptome and analyzed in silico. The 21 dpa RNA-seq libraries used here are public datasets deposited in the NCBI SRA under accession numbers SRX1411321, SRX1411322, and SRX1411324.

### 4.3. GO Term Enrichment Analysis

Gene Ontology (GO) term enrichment analysis of the predicted proteins was performed using the DAVID (Database for Annotation, Visualization, and Integrated Discovery) tool [42]. To achieve a good balance between specificity and coverage, GO level 2 terms were used. The graphical representation considered the number of lectins expressed at 21 dpa and 2 and 7 dpi time points, and GO terms were considered statistically significant when *p*-values ≤ 0.05.

### 4.4. LpITLN2-B Analysis

Domains and motifs were predicted using the SMART database (http://smart.embl-heidelberg.de/, accessed on 2 May 2023). Multiple alignments with intelectin sequences from the UniProt database were performed using Clustal Omega (https://www.ebi.ac.uk/jdispatcher/msa/clustalo, accessed on 2 May 2023). Sequences used in this study were the following: *Oncorhynchus mykiss* ITLN1, ITLN_ONCMY (UniProt Accession: P0DMV4); *Mus musculus* ITLN1-A, ITL1A_MOUSE (UniProt Accession: O88310); *Homo sapiens* ITLN2, ITLN2_HUMAN (UniProt Accession: Q8WWU7); and *X. laevis* ITLN1, ITLN1_XENLA (UniProt Accession: Q5PPM0). Physicochemical parameters were measured using the Expasy ProtParam tool (https://web.expasy.org/protparam/, accessed on 21 May 2023). The active site residues of all the protein models were calculated by utilizing the COACH server [43].

### 4.5. D Model Construction

The protein structure 6USC, a human Intelectin-1 obtained at the Protein Data Bank (PDB), was used as a template, and the homology modeling of LpITLN2-B was generated via sequence alignment and structural modeling using Modeller v10.4 software [44]. The predicted 3D structure was refined to reduce side-chain steric clashes and bond length errors using the Galaxy Webserver [45].

Model quality assessment was conducted considering the RMSD (root mean square deviation), which evaluates the deviation in the model from its template (6USC), checked with ERRAT [46] and Verify3D [47]. A Ramachandran plot was created through PROCHECK [48] to verify if the model was folded correctly.

### 4.6. Molecular Docking

The molecular docking calculations were carried out using two molecular docking programs, and the interactions between LpITLN2-B and carbohydrates was performed using GOLD (Genetic Optimization for Ligand Docking) [49] and DockThor servers [50] (https://dockthor.lncc.br/v2/, accessed on 7 July 2023). GOLD was used to obtain a conformational search on the binding site to improve the hydrogen bonding network between the protein and its ligand. Hydrogen atoms were added to the protein, and ten docked solutions were ranked according to the GoldScore function and re-ranked using the ChemScore function [51]. DockThor was applied to validate and corroborate GOLD results, allowing ranking consensus.

Virtual screening was performed for the following carbohydrate 3D structures, all of which were retrieved from the ZINC database [52] (https://zinc.docking.org/, accessed on 5 July 2023) in mol2 format and subjected to molecular docking analysis: arabinose (Ara), fructose (Fru), fucose (Fuc), galactose (Gal), glucose (Glc), lactose (Lac), maltose (Mal), mannose (Man), *N-*acetylglucosamine (GlcNAc), rhamnose (Rha), ribose (Rib), sucrose (Suc), lipopolysaccharide from *Escherichia coli* (LPS), and polyinosinic:polycytidylic acid (Poly (I:C)). LigPlot+ was used to analyze 2D molecular interactions between the docked ligands and the amino acid residues of the protein [53], while the 3D visualization was performed by PyMol, showing the structure, hydrogen bonds (H-bond), and its length.

### 4.7. Hemagglutination Protocol Assay

Blood samples drawn in Alsever’s solution were stored at 4 °C until further use. Erythrocyte suspension (2%, *v*/*v*) was prepared in PBS. Tests were conducted with *L. paradoxa* macerated skin samples obtained at 2- and 7 days post-skin injury (dpi). Hemagglutination was determined visually through the appearance of mat formation as an indicator of lectin activity, whereas button formation was taken as an absence of lectin activity. Lectin titer was defined as the inverse of the highest dilution capable of producing the visible agglutination of erythrocytes. The carbohydrates tested as inhibitors were D-lactose, D-maltose, D-fructose, and sucrose at 100 and 200 mM concentrations (Table 2).

## 5. Conclusions

The transcripts of lectins derived from libraries constructed with skin samples from the injured South American lungfish *Lepidosiren paradoxa* have unveiled the presence of an intelectin identified as LpITLN2-B. This intelectin demonstrates a pronounced affinity for disaccharides, particularly D-maltose, suggesting a potential involvement in modulating innate-associated immune responses. The inhibition of lectins activity in the presence of maltose supports our in silico findings. Moreover, LpITLN2-B exhibits recognition capabilities toward poly(I:C) and LPS molecules, hinting at a prospective antimicrobial activity. A comprehensive exploration will be undertaken in forthcoming investigations into the lectins and intelectins of the South American lungfish. This will involve delving into experimental biochemical assays to further elucidate the intricacies of LpITLN2-B and validate the robustness of the data presented in this study. Concurrently, efforts will be directed toward characterizing the structural attributes of LpITLN2-B, with a concomitant evaluation of its efficacy against pathogenic entities.

## Figures and Tables

**Table 1:** Molecular docking results for LpITLN-B. ^a^ GOLD program result. ^b^ DockThor server result expressed in kcal/mol.

Ligand	Docking Score	
	GoldScore Function ^a^	Binding Free Energy ^b^
Maltose	72.46	−7.69
Lactose	70.71	−7.62
Sucrose	69.68	−7.56
Poly(I:C)	64.91	−6.68
Lipopolysaccharide	61.02	−6.19
Fructose	64.02	−6.89
Ribose	62.60	−6.76
*N-*acetylglucosamine	62.13	−7.13
Galactose	60.72	−6.89
Glucose	60.71	−6.84
Mannose	60.53	−6.77
Fucose	57.06	−6.86
Rhamnose	57.06	−6.79
Arabinose	55.91	−6.59

**Table 2:** Relative hemagglutinin inhibition activity (%).

Concentration (mM)			Carbohydrates			
		Control	D-Lactose	D-Maltose	D-Fructose	Sucrose
2 dpi	100	100	133	66.5	133	N.D. *
	200	100	50.7	50.7	101.4	50.7
7 dpi	100	100	66.5	66.5	133	66.5
	200	100	50.7	50.7	50.7	50.7

* Not Detected

## Data Availability

Data is contained within the article and Supplementary Materials.

## Supplementary Materials

The following supporting information can be downloaded athttps://www.mdpi.com/article/10.3390/ijms25094798/s1.
