# Supplementary material for: Potential Involvement of the South American Lungfish Intelectin-2 in Innate-Associated Immune Modulation
Source: Int J Mol Sci. 2024 Apr 27;25(9):4798. doi: 10.3390/ijms25094798 (PMC11084424; doi:10.3390/ijms25094798)
Supplement: Supplementary file 1 [file ijms-25-04798-s001.zip › Supplementary Gene Sequences.pdf]

Supplementary Material. Transcriptome Gene Sequences

>ITLN2_TRINITY_DN388_c0_g2_i4
GGAACATCAACAACTCCGAAAGTGCTTTTCATCTCTGTAAATTCAAACGTGGTAGTACT
TCTAATCTGTAGGTACAAATTATATATGTGATTATCTGAAGGATGGCGATATCCAGTTTG
CTTATGTTCAGTGCTGTGTTGATGGTGGCAGAGACAGATTCAAACAACAAACAGTGTAA
CCTGAGAATTCAGAAGAGTGTTTTACTTGAGAGTATTGCTGGCTGCTCTGATCACACATC
AAATGATCAGGTTACAAACAACACACAACACCACACCCAGCAGAAGTATATGGCAAA
AAGCTGCAAAGAGATCAAGGAAAAGTATCAAGTCAAGCAAGATGGCCTGTACTACCTA
ACTACAGAAGATGGTGAAGTCTACCAGACATTCTGTGACATGACATCGAATGGTGGGG
GCTGGACTCTCGTGGCCAGTGTTCATGAAAACAACATACATGGGAAATGCACATATGG
GGATCGCTGGACCAGTACTCAAGGAAACAGTGAGAAATATCCAGCAGGAGATCAAAA
CTGGGTCAATCTGGCCACATTTGGTTCAGCAAGTGGTGCAACCAGTGATGACTATAAGA
ACCCTGGATACTTTGACATTGAAGCTGAGGACATTTCTGTCTGGCATGTCCCTAATAAC
ACACCTCTCAAGAAATGGAAACTGGATGCCATTCTGCAATACCACACTGAAAACAAGT
TCTTGCCCAAGTATGGAGGCAACCTACAAGGGCTCTTTAAGAAACTGCCTCTTATGTTT
AATATTGGAAGCTGCCCTAATAGTAATGGTCCAGCCATACCAATTGTCTATGATTTTGG
AAATGCTGAGAAAACAATCAACTTATATTCACCCCTCTCCAAAAATGAGTGCATTCCTG
GTTTTATTCATTTTCGTGTTTTCAACTATGAGAAGGCTGCCATGGCAATTTGTTCAGGGGT
CAAAGTAAATCTGTGTAACACAGAGCATCACTGTATCGGAGGTGGTGGTTTTTTCCCAG
AAGGTAATCCAGCACAGTGCGGAGATTTCACAGGATTTGCATGGGATGGTTATGGAAC
TGGCCAAGGTGCTAGTGTAACCAAAGAGATGCTGGAATCAGCTGTTCTCATATTTTACC
GTTAAAGGACTTTGACTTTGGTGAATAACAGTTTCACTCAGCTCTTTTAATCTGCATTGC
ATTATTCCTCACCAACATTTATTTTGTATTTTCATGAATACTCATTAGTGCTGTATACTTTT
TAATTACACAATCAAAAACCAGAAAGCTGAATTTCTGTTACTTGCACTGCTGTAACAGA
AGTTCCATCTTCCACCAATACTCCCAAAAGGAGGCACCATGGCAGTGAAGGAAATTCA
TAGACCCTTATCAGGATAGTTATAGCATGAGAACAGGCTGCAGCATAATCAACAACAG
GCAACAGGAAACAGCTGTTAGAACATTAGAAGGCCACAAAAGAAACAATTTATTTTAA
CTGTAGACCTTCCTGTCGATTGTGCATCTGATTTTTTAACAGATTCAGAATTCATCGGTG
CTGCAGTAAGGTAGTAGAGTAAAATCTAATAGCATAGGTGGGATTAGCTGCATTCATTA
ATTTAAATGTTAGTTAAGTATGACTCAACAAATATTGTGTGTTACACAACTGTTTCTAAC
TTCCCACACTGTTAGATCAGGTTGTGGCTTGCACTGCTGTGTTATCTTGATGAAAATTAG
AATTTTAACAAAGAGCTTTAATACCAGAACTTCTATTCACCTCTTTTTTTACTAAATTTAT
TTTTCAGATCTGCTTACCTGGCTTGCTTCAGTTGTTTGAAGATTCATTTTGCCCTAAAAAT
GGTAGCATTAAACAATTCTGTTTCAGCAATAAGCTCTGTTATGTGCTTGCTTGTTTGTAA
AACTACTGTGACTTGAAAGTGCAGAGTATGGCTGTTAAAAACACTGACATCAAATTATC
TCAATTTTTCCCATTCTAAGTTCTTTTATGCTACAAGCAAATTCTTTGACATTTGTTATTTA
ACCATGTTTCTTCTATAGGATATGATAAAAGCATATTACAATTATGGAATGTATATGAAT
TCATGATTAGGACAAAATATTATGCAGATTATAAAACTGAACATACAGAAATATAACTT
TAATGGCATTATGTATTGAAATAGCAGCATGTTAGTGAAATAAAAATGAATGAGTTTAA
CAGTGGGTAGAGCAATAAACTA

>LGALS1_TRINITY_DN18437_c0_g1_i1
ATTTCAGACATGGCAGAGATAAAGCATGCTACCTTAACTGACCTGGATCTGCGTCCAGG
CACAGAGATTGAAGTTGAAGGATTTATAAAGCCTGACGCTCAAAGCTTTTCTGTAGAAC
TGGGAAAAAATGATGACACTGTTCTCCTGAAATTCAATCCTTGTTTTGATTACAAGGGT
GTTAAAAAGACAACTGTGTGCAGCGCTATAATTAATGGAAATTCTGTCGCAGAACAGA
ACATCCAAAACTTTCCCTTTCAGCAAGGAATGTCAACCAAGATGCTGTTCAAATTCAAT
GGTGCCGAAGGCGTCACTATAACATTTCCAAATAAAGATAAAATAACCTTCGCTAACC
ACACAACCATTTGGACAATAACTTATATTACCATCCATGGTAACATCGATGTGAAATGC
ATCAGGATTCAGGACCAGTGATGTACAATATGACATCTGCTGTATACTACATGCTACAC
CACATTTGTCAGCTTTGTTTCTTCATAATTATGCTGATTTTTTAATATACAGATTTGAAT

AAAGCATTTATTAACACTTGTTCTGACGGACAGATTTGTTTATAAAATGAATCTTCATGC
ACATCTCTGATCACAATACTGATTCTGTTTATTACTAAATTTAGGGTCCAGATTATAGTT
GTAGACTGAAAGGTAAAATTACTCTGTTATCTCAGATCACTGAAAATGATGTGCTGTCC
CTTGCTTATTTTAAATATTTATTTTATCCTTCAGCCATAAGCAGTCATAATTTTTTCCTGGT
GTTAGTTTATACTAGTTTGGGTTATTCTGTGACTATGTGCGCACAATAGAGTAACTATGT
AGCTACTTGCTCCACATGTTTAACTTACGCTATTTCTTCCAAGTGTTCTGAACATAATCGT
TGCTTGTAACATGATGTCTTGTTTACTTGTTTGTTATAATCTGTAACCCAGGCTGTAACAT
GGCTCTAGAGGTGGGTAAAGTTAGAAATGAGCAGAGGTGGATGAGGAGATGGAGACC
TATAGATTGGAGGGCAGAAAAGGGAGGAAGAAACCAGAAGGAAGGGGCTGCAGAAA
TATGGAGGAAACTGAGAATGCCACTTAAGTACTGGGAACAGATGGTAGTACATGAGGA
GAAAAAAGAATGGGGGCAAATAAGAGAAGAGTATGGGCTTGAAAATAGACATATACT
GAGATACCGAGGTTTAAAAAGAGAAGAGAGTAAGACAAGAAAAGAAGAGGGACAAA
TAAAAGCAGAAGAATGCCCAGAGTAGATAAAGGTTCTTATAGAACCCAGAGGAACAG
TGACAAAAATAATACTAAATTGATATCGAGGGCATACCAGGGCATAATAATAGGGTAT
AAGAAAGTGAGTGAACACATGAGACAGACATGGCAAATGAGATGGAATAGGGAGATA
TCAATAAAACAATGGGAGGAAATATGGAAAGCCCCAAGATATGCTACAAAGTCCATG
AAATTTAGAACTTTTGCATGGAAATGCTTTCACAAGTATTTCTATACATTAGTGAGGCAT
AAGAGGTTGTTTCCACAGACAGATGAAAGGTGGTGGAGATGTGAGACAGGACAAGTTG
GAACATGGAAGGATATCTTTTGGGAATGTGAAAAGCTGAAGGGAATTGAGGGGGAAGT
CAAATAAATATGGGCAAGGGCAAGTCTAAACAATAAAACATTTACAGAAATGGGAAT
GGACATAGAGATAGACCTGACCTGGAAGGCAAATAGAGAGGTAAGAGAACTCCTGAC
ACTTATAATAATGGTATACAAAAAAGCAATTACAAAGAAATGGAAAAATAGGGAGGT
CCCAGAGGTCACGGATCTTCATGATATCATGTGGGATATGCAAAAGATAGACTGCTGG
GGCTCCCAAGTCCCTAAAAGGGTTAAGAAAAAACGTGGTATTTTGTCAGCATGGAGAA
AAAGAAGATGAAAAATGACAGTTCCTTTAGTTATTCCTCACAGAAACCACTATGGGAT
GAATGAATGAATGAATAAATGAGTAAGAGTGTACAATAGAGGGGCGAGACATACGCG
CTCAGCCTTGACATGTAAATAGTTGGGTAGCAAGGTTTGAAATTAGCT

>LGALS9_TRINITY_DN2268_c0_g4_i4
AATATGAATGGTACATGCTTTATTGTTTCATTTGGTCCATGAAAATGTAATTGGTGAAGT
GAAGCTTTAGGAGGTAACTGGACAAATGATGATTACTGCTAAAAAAAAAAAATACATAT
CTGAAAATTTCAGACATGGTATGACTGGACAGGCATTTACTGTGTTTTCAACAAAAACA
GGTAGATACAGTAAGGCTTTCATTATATAAATGATACAAAACAGATTATACAAACACT
GAAACATGAGAGTGTGACTAAACCTGAAATATTCTGAACAGATTTACATTTTTGTATCT
CTGCTTAGACAACTAAGTTTGAACCTCCAAGTTAACTATTATCAACAATTTATTTATTTA
TAACTTTTGTTTACTGGGAAGACGCCACTGGAATACAAGCCCATTTTCAGTGGGTGCCC
AGGGAGAAAAGCCCCACATATACAACATTAAACAAAGACATAAAAAACAAAACATTT
TAAGTTCCATGGGTACACAATAAGCTTCAACAACTGTTAACACACATCAGTAAATACTA
AAAGTTTTCACATTTTTTTTAGAATAAAATTTATAGAAGTATTTAAGCAATTTATACAAA
TGACAACGTGCCTACATTTTCTCTCACACTAACATCTGATACATTGTTTAGTAGTTAGTC
CATAAATGGTAATGGTTACTAAACTAAGACAGAACTATAGAAGCTACAAATCATGCTA
TATGTTTCTAGAACTTATAAAAATATTCTGAATAATGATTTTTCCTTTCAAAAATACACA
CTTGTAAGCTGGACATTCCCACTTATTTCAAGCTTCTTTATTGACTGAAGGTCAGATACT
CGATGGTTGAAGTTAAACATGTTTCCATTTACACTGACTCTGAAACAATGATGTTCACA
GATAATGGAGATCTGAAAACTCTGCCCTTGGAATATGGGCATTGTGCCATTAGGCAAAC
TCCGTTCCTCTGCTCCCCAAGCATTATTCAGAAAACTATTTCGAACTAAAGCATTTTCTC
CAAATCTTACAGCCAGATGAAATGCAATGTCAGAATTCCTCTTCAGGTCAATACGGAA
GCTGTCAGCATGATGAGGCACTGATCCAGAAATGGCAATGTTTCTTGGTGGATACAGCC
CCTCTGGTATGTAAACTTCATATGGAACTTGAAAATTAGCTGACTGAGCTGCAGCAGAC
GGGTAACCGGGAGCTACTGGTGGTGCCGCACAGTAAGTGGGGAAAGCAGAAGCAGTT
GGTGGTGCCGCAGAGTAAGCGGGGAAAGCAGAAGCCGCCGAAACAAAAGCACCTGAA

GATGGAAGCACTTGTGAGGCCCGCATGTCCTGAAAACTGATGACACTCAGCTGAATCTC
TCCTGACACACCGATTGTATCCACCCTGGACAGAGGGATCCTGTGTTTATATTCTAGGA
AATGTACCCCATTCACTGCCACCATGAAGGAAGTAGTTTGCGCAAGAAAAAGAATTTC
AAAGCGCTGCCCAAATTTAAAGGGCATTTCATATTTCCGCTCTTCTGAACCCCATGTCTC
ATACTGTAAAGTATTGCACACGACACAGCTCTCGCCAAAGCGTGGGTTGAAGTGGAAT
GCAATATCGGAACGAGGAGTCACATTGGTTCCACACTGGAAATTCACAGCAAACCTAT
CACAGTAGCTGTTGACCTGTCCTTGTACTGCCACTATTTTTCCATCATAAAGACCACCAT
GAATGGCACCAGTAAAGGGAATTGGGGGACTGTAGAAAGGCGCCTGAGTCTGAAACC
CCGACATTGTTCGTACAGCCA

>CLEC2L_TRINITY_DN98733_c0_g1_i2
GGATTTAAGTTTTATTTTATTTTTCTGCAGTGTCATTTATAACCACATTTGAAAACATCCT
TAAAAAGCAAACAGATATTGAAAGCTTTATAGTCTTAGTCTTTATTTTTATGTCATGCTG
TCCAGTAGAGCAAACTGTTTTGTTAAAAAAAAAGGTTGCTTCATTAGGATAGCTGTACT
TTACCATTTTTTTTCAATCATTTTACAAATATGATATTGTGACTCCCTTGCAGAGCTGATA
AGACAGCAAAAAGCTATACCTAAAAGTAATCAGAGGTACATTCTTTGGAGAAAGAGCA
CTCTACTGATATATCATCATTTAGTTCTTTGGATCAGTACTGTCCAGTGTTGCTCTGGCAG
ACAAGCCTTCATCCCTTCACATATACATATTTGTAGCAATATCATCATAAACGTATATCG
CTTGCTCTTATTGCAAGTGGTTAACTGCTTTCTTCACACAAATATAAGGAAAGAGTGCTG
AGCAATGACGATCAAATAGCATATCAGGTTCAATAGTGATGCATTTGTGCTCAGGACTA
ACACTTCCTCTATTGATTCTAGTATCATTACCATCAATACTGAAGAACGTATCAATCCAG
TGAGCTTTATCTTTTACAAATTTTGCTGCAAATTCCAACATGTCATTGTTTAGATGTGTAA
ACAATGATGCATTCTGAGAAGAACATGACTCTTCTGAGGCATTGTAGTTTTTTTCTTCTTT
GGAAAACAGGTAGCATTTGCTTTTGTATCCTATCCAGTGTTCTGGGCACATATAGTAATT
CTCTGTACCGTTACCATGTGCCTCTCTGAATAGTCCTCTCTGATGTTCCATCTGTGCATTA
TTTAACTTTTGAGTATATTTATGAATAAGCAGCACTTTCCACGCAAATATCAAGGCAAG
ACAGGTGATGCTGAGTGCAGTCATGGAGTAATAACAGAACAGCAGCTTGCCCATTCTCT
GAGCTGTAGAACTCTGTTGTCCAATTCCACCTTTAATTTTTTCACACTTGTATCTTCGTAG
CTTTGTCAGTGT

>CLEC2B_TRINITY_DN2263_c0_g2_i4
AACTGTCGAACAAGTTTCGGATGATGAGAGGTTGAGAACGATATATACGTGTGGTTATT
AAGGAAATGCAGACTTTACTTGAGGAAGCATGTTTCTGCAGTGCTTGTCATGTGCAGGG
GAGAGCAGGAAATTGGAAGGAGGACTACAACTGTTGCTCTCATATACAGCATTAGAAA
TGAATTGTCTCCTTCTAGTGACAGCTGTGCTTGCAGAAGGCTCCATATAAACTACAATA
AAACTTTTGGAATCGCTCCCAAAGTGGCTTTGCGGGGTATTGCAAATTTCCATGGAGGT
GTGCCTATTAAATTTTCATTGGGCTGAATTTCATTGAATATATTGGATTTGCTTTAGAAAC
CGTGGCCCGCGACGCAAATGGTTTTGTAACATGATTAGAGCAAGGAGCACCGAGAACC
AAAGAACACCACTCAGGATGTCTTCATCTGAGAAGTCTAGCAGATCACTCGCAAACTT
AAGTTCAGATATTTCTTTCAAAACATTAGTGTTTGTTGTGCTGACTGCAATACTATCAAT
ATTCCTTTTGTTAAGCTATATTGCGCCTCACTGTGAGATGATTTATAGAAAAAGTGTTTC
GACTACAGATAGAGCTGCAAAGGACTTGGACATCTTAAACAAAATGAAAGAGCTTTGC
CATGAATCAAAGGCTAATGATAATGAAAATGATAGAGCTGCAAAGGACTTGGACATCT
TAAACAAAATGAAAGAGCTTTGCCATGAATCAAAGGCTAATGATAATGAAAATGATAG
AGCTGCAAAGGACTTGGACATCTTAAACAAAATGAAAGAGCTTTGCCATGAATCAAAG
GCTAATGATAATGAAAATGAAGTTTCTTGTAAGTTGTGTCCTCATGGATGGATTGGAGA
TCCAAAAAACTGCTATTTCTTCTCAACTATAGAAAAAGATTTCAAGAGCAGTGCAGAGT
GGTGTTCTTCAAGACATGCATCCCTTGTGCTGATTAAAGATAAGGAGAAACTGAACTTC
ATAAAGAACTGGGCCTACAGCAATACATACTATATTGGACTGAAGCGAAATGATATGG
GACAATGGATGTGGGCTGACAATACATTCCTTGATAAAACGAGATTCATACTGGAAAA
CACTACTGAACTTAACTGTGTTTTTATGGAACAAGATAAAATTCATCCAACTTCTTGCAA

CTCATATCATCGATGGATATGTGAAAAACAAGTTACAAGAATATGGTGACCAAATAAT
GTTAAAAAGTGCAAAAAAAATTCTGGCTCTGTTTTTTTTTTTTTTTTTATTG

>CLEC4E-A_TRINITY_DN90481_c0_g2_i3
CAGTAGGAAGAGACATATCTCAACACGTATCACCCAAGTAAGATTTGACAAGTGTTTC
ACTTTTTATTTCTTATCAGGGGACAGCAGACTATTTAAAAAAAAGTCATATTGCCCTCTT
CACACAAAATACCCTATTGATGCTTTCTCACAAATCCAGTTATGTTTTATTGAGCATTTA
ATGTCAAGCCATGTTCCGTTTACTTGCAAAACAACACAGTCATCCACATTTGACACTGC
ATCCTGGTTGTTAGGTCTGCCTTGTGCCCAGAACTTGTCTGTGGCGGAAAAATCAGTGCC
ATCCACCCACTGAAATTTGCCTTCTACTGTCTGGTCACTAAGTCCAATGAAGTATGTTTT
ACTTGCAAGGGAACTCTTCAAAAATCCCTGTTCTGCTCCATCATTGATGATAACAAGCT
CAGATTTCATAGATATGCATCTCTTATAAGCAGTATTCCAGTTTTCAGTCTCTGTAGAGA
AGTAGTAACATTTTTCTTTATATTGCTTCCATGGGCTCGAGCAGGGAGAACATAAAAAT
CCACTTTTATTGGCAGATGTCTTCTTGCACAATGCATTGGAGAGGATTTTGCTGTCACTC
TGCAGGTCTTTGATGCTTTTGGCTTGACCATCATTTTTATTGTGGAGTTCAGAAATTTGGG
ATGACATCTTCTCCTCCAACTGTATCATTTCATCAATCAGTGCATCATATTTGCGGTATC
CCACTGTGAAGAATGCTATAATCAGCACAAAGCAGATCATTAAAAAAATGACTAGTAT
GATAAGGAGCTTTGCTGGCACCACTCTCTTCCTCGCTTTCTTCATAGATAAAAATGCTGA
GTTGTCTGGACTTCCTCCATCTTGTTTTAGTGGGTTACTTGAAGTATCTGGCTTTGGAACA
GGAACATCTAAAACACAAATGTCATTCTTTCTTTCTTTCTCTTTCTCCAGTGGTATCCTTA
TTGGGGCGTTCATGTAAACGTGTTCCATCTCCATG

>CLEC4E-B_TRINITY_DN68583_c1_g1_i2
GTGTTCAGCAATACACTGTGATGCAGAAGGAAATGGCACAATACTTGCAAAGTTTTTAG
AACACTTGTCCCTTTTCATTAACATTTAAATACTAAAGAGCAGACATGATTTATGAAGA
GCTTAATCTTCCAACTCAGGACACTTACAATACAATCGAGCAGCTTGAGACTGAGAGA
CAAGGGAAAGAAATGACACCAGGACTGAAGAAAGCTCCGGCAGTACAAGATGATAAA
GTGAAGCACAACAATGGATGCACAAAGATGCTAATTACACTGGCTGTCACAGTGATCA
CCCTGCTGATAATAATTATAATCTTAATTATTCTGAATCTACAGATGTCAGAACAGATA
ATTCAGTACAAGAGGTCATCTGTGTTCCCAAAAATTACAATAATGACAAATGGAGGGA
GTTGTTTCTTATCATGTCCAGATGAATGGCTAGAGTTCAAACAGTCGTGCTACTTCTTTTC
AAAGGACAGCTTCAATTGGAATATGAGCAGATATGCCTGCATGGCTAAGAAAGCTGAT
CTGGTGGTGATTACCAGTAAAGAAGAACAGGACTTTATAAACCATCACTATATTGACAT
GGCCTGGATTGGTCTAACAGATAATGAGCAGGAAGACATTTGGAAATGGGTTGATGGC
ACACACTATGACAACAAAACCAGCTTTTGGTGTCGTGGAGAGCCTAATAATGCACTGGT
GCACAATACTGAAGGTGAGGATTGTGCCATGCTGTGGAATACTACATCATGTCACTTAG
GCTGGAATGATTTAACTTGCCAAAGAGACTTGAAGAGAATTTGTGAAAAACAACTCTG
CACAGTGCTTATGTAAACAGTGGCCTATGCAATGACTTTGACCAGCAGAAAATGTGAG
AAAGTATGTATGCCGGTGAAATATTTGGCAGTAGCTGCCATTTTATTTACACAACCTCAT
TAACAACATTAACATCAATAAAATTTGTTACCACAAATAGTTAGTGTCTGGCCTGTAAT
GTTACTTTATTCAGTCTTGCACCCACTTTTAGTCTGTAATCAGTGACATGCAAAAAGTTT
TTAGACAGTATGCTATCAGTTTATTTTACTGGCATTTATCCTGGTTGTATTTCCTCTACAG
ATTCTCAGAGTTGTTATTGTACTTATGATATACAGCACTTTTGGCTTAGTTTAGTTACTGT
TTTTTTTTTTTTT

>CLEC4E-C_TRINITY_DN46240_c0_g2_i1
GAATGGAAGATGAGGTTTACAGGATTTTTATAATCTCTTCCACAATCAATCATACTTTTG
CTTTAAAAGGCAAATGTACTTATTAGGTTAACAGTTCAAGAGCTAATATTGTTGAAGTT
GTGTTGCAAAATAATGATATGACAGCTTCTAAGTAACTGCAGTGAATCAAAATAACTTT
TTCAACAAATTGCCATTGTACCATAATAAAATGCATACATTTTTAAATAACAGCAGAGA
TAATGATAAACTCAAACAAAAAAGTTCAGTACATAGTTTTTCCTTCAAAACCTTGTTTCT

GTGACAACCATTTTCTTCATGCTGACATCCACAAAAGTAACTATATAGGAACTATGTGA
ACCACTAACTGAAATAATGAAATAATATGTTACTACTGAAACATTAGGAGGATGGTTCT
GTATTTGTCTTATCAGTCTGACTTTGTAAAAGTTAAACTATTTAAATAAACAGTGTTTTA
GCACATGTTGTTGTTTGCTATATAGTCAGCCAAATCAACAATGAAATGAAGCAGATTTA
TAAAGAGTTGCATTTGATCTGTTTTTCACAAATAAAATTGAAGGACCGTTGGCAGTTTTC
ATCATTCCACTCCAACTGACAAGGAGGCCTAGTCCAGAGGGCTGCACAGTCTTCACCCC
TGTGATTGTCACCCTTTGCATCATTAGGTTCACCTTTGCACCAAAATCCAGTTGAAATGT
TGTGTGGTGTTCCATCTATCCAATGCCATGTGCCTTCCACACCTTCATCAGTTAAGCCAA
TCCAGTAATTGTTTCTACACTTATTTTTTAAGAAATCCTGCTCTTCCTTGTTGTTGATCACT
ACTAAATCGGCACCCATTTGCTGACAGTATTGTCTGCTGCCGCTCCAAGAGTCAGTGGA
AGTGGAGAAGAAATACAGGGACCTCTTGAATAAGTTCCAGTTTTCAAACAAGACTGAG
TCAAGCACACAAAGATGTCCAATTTCCGAGGGTATAAAGTATCCACTTTTTAATTCAGG
CAGCGCTTTTGATATTGGGCTGTAGTTACTAACCATCTCTTGCATTTTCTCTGTTTCTTGTT
TAAGAAGGTGTGACATTTTGTTATAGTTCACGCTTAGAAGAATAGGGGATGCAAGCAG
ACATGTTGTAATGAACAGCAGAACAATTATCAAAGACCATAGTATGGGTTTCTCATTTA
ATTTATTCCATAGTGTCTGCCCTGCATGAGAGTTTAATCCAAATACATTCTGTGCAGTGG
CTTTATCCATCTCAGGTTCAGAAATAGCTGCATGGTCATTTTCAGATTCAAAAAGCAAA
GAATTATATCGAGCATCATATTCCATTGCTGCAAGTTCTGCTGCTTATTTCCTCAAATAA
CTCAGTGTTAGTGA

>CLEC4E-D_TRINITY_DN23661_c0_g1_i1
AAAAATATGATTTGGATGTTATTATTTCAACAGGTTTATTTTGTATTTACACAAAGTTCTT
CTTAATTTTTATATACAGTTTGCTAGGCTACCCAAAACAGTTCTGCATGAAGAACCTAAT
TCCCCATTTTTAATTTCTGTTGCATTCACTTTAATGTTATTCTGCATTATAAAAACTCTAT
AGCAAAGTAATTCCAAAAATAGGAAAAAGAGGAAAATGTGCACAGGGAACCAATGTG
CAGTTGGAGGGGCTCGGTATAATGGTGAAATTGCTTTCTTTTCAAATACCATTGACTTAC
AACAAATAAAATTAAAACAATATTTACAGATATCATCTATTGCCACATTAGCATGAATC
ACGTAGTTTTAGGTATGCCTATGGATTAGAAATTATGTCATAAAGTCATGGTCAGCAAG
AGTTTCTTCCAGTGAGATTAAGTCTTGTTTAGTTTCTGACTGGCAAGTTTTAGCCAACTG
ATGATAGTCATGTCCACATTAGCATGTCTGCAGGTCAACAGCTTGAGCCCCACTCCTGT
CTGAAATGATTCCTTGCACTCAGCAAGGATGGTAACCAGCAGTCATAACTCAAATGGTC
ACACACTGATGATCGTCCCTTTTTTTTCACAGATGTATGAATAATTTAAATGACATGACA
AGTCATATGTGATAAGTCCAGAAATTGAAGAAATGATTATTGCAGCACAATCCTCTCCA
TTAGAATCGTGACGCTGGCTATTATCAGGCTGTCCAACATCCCATGCCACTTTTCTTTTG
CTAGTATCTTTGCCATCGACCCAGACCCATTCACCTTCTTTTTCGATATCTGACAATCCA
ATCCAATACTTTTCACTTTTATTTGCAAACCAGCTAAGAAATGCCTTCTCCAGGTCATTG
TCTATGACAACATAATCAGCACTCTTATTCTTACAGTCCTCCCTGCCTTGCTCCCAGCTT
CGTTTGCTTGCAGTTGAGAAGAAGTAGCAGCTAGTACTTAATGAATCTCTATCACTTGG
ATGGAATGCTGTCCATCCAGGTGGGCAGATCTGACATCTGGAAATTGCACCACGTTGCA
CACAAAATGAATCTTTCAATTCAGAAAGTGCCTTAGAAAGAGATTGCTTGTCAGATCTC
AGCTGGAATATTTCTACATCTTTCTCATTCGCAGGTCTAGAAATTTCACTGCAGTTGAAA
GGAATCTGTTTGACTTTTGATGCTTCATTCAACTCTGCAGAAACAAAGCTGAAATTTTCT
GAAACCTCCCTGTGTTGGGCAGTTAATTTTTCATATTGAGCCGAGAGTAATTCTGCTTTC
TCTGCCTGATGAGAATGTTGCTTTGATGCATTGTTATACATCGTCGCAATGGCTACAATC
CCAATCAGCAGGATGCCGCAGAGAAGGAGAAAGGCTGTGACAATGAATCTGGTTCGCC
AGGATATCTTTGATGTGTCCGAATCAAAACCAAAAATGCCTGTTTTACCTTCCGCTGCTG
GCATTTGTATAGGTGTGTAGATGTCGCTGTGCTTTGGTGGATTCAGTTTGCTGTAGGATT
CTTCATTAACTGGTTGCAGTCCCTCGTATATGTGCTCATTTGCCATTGTTGCTTTCTCTGG
CTCTTTCTTTCTATCTCCTTCTTCCATCGGGACTTCCATCCCCTCAGGTCTCCCAAACCCA
TCCTACCAACCTTGCCTGGGGCTGTATGAGACATGGGGATCCCGTGGTCAGGAGTCTGT
GCATCAGCCTGGTGGGTGCATGTGTGTGAGCTCCATCCCATCATGG

>CLEC4E-E_TRINITY_DN383_c0_g1_i7
CCTTATTCCCTAACTACTTAGTCGCAGATATAAAACTTAAAAGACATTAGCAAAAAGTA
GCTTCAAAGGCAGGTGACAGAACTTACAGTAAGATTTATTCATCAAATTTATTTTTAAGT
ATTAGCTAATTTTTGCATTTGATGAACATTATGGAACAGGAGAAGACATACCAGGAGCT
TCAGTTTGCAAATGAAAATATTTACTCGGAACTTGATCAACAACAAACTAAGAAACTA
GGAGCAGAAAAGACAGCTCCAGAAACACAAAAATATGTGGCTAAGTATTGCAAACAA
CCAAGAAAATTGTTAATCACCTCCATTATCACAGTGTTCATCCTGCTGGCAGTAATCAT
AGCTGTGATTATTTGTATTTACCAACTTTTTCCGGTGCAGCCCCAGCCCAGCACATCTGT
CGCCTATGGCCCCATGACTGGAAGTCCCCTTGATGAACATTTTAAATGGCATACAGCAA
ATACTGGCAGAGTTAGAGCACACACCAGCCAGCAACAGCCAGCTACAGGCATTTCTTC
AACTTGTCTTTTAGAGGATGCAAAGATGTTTCAAAAGAGCTGTTATTACTTCTCAAACA
CAAGTTTAGAATGGAATGGCAGTAAGAAATGGTGTGAAGACAAGAAAGCTCATTTAGT
AATAATCAAAAGTGAAGAAGAGCAGAAATTCCTAAAAGACGAAGTTACTAATTTTAGA
AACACCTTTATACAAAAACCTACAGGTCATTTTTTCTGGATTGGACTTACAGATAAATTA
AAGGAAGGAGAGTGGCGCTGGGTAGATGGCACTATCTGTGACTGCAACAATACTAACA
AAAATAAAAGATACTGGAGGGAAAAGGAGCCAGACAACTGGAATAATTTAGAGCACT
GTGCTGCACTTGCTGAGAATGGTGACTGGATGGATGGGAAATGTAACTGGACTCTCAGG
TGGATTTGTAAGATGGATGCTACGATTTTCTCTAGCTAATCAATTTTCTCAATGACAGTT
GTTTAAATGTGACACTGATGTTTGATCTAAACAACCATTGTAACAGTCTTGAACATGTTA
ATCATTCAATGATTATTTAATTATTATTTGGTTTGTTCATGAAAGACCCAAGTAATATTG
AGCACATGGACAATAATTCTGCTGTTGATGACTTTCTCCATTATTTCATGTATATGTTTAG
TGTTATATGCCTATATTCTAATCTCTAATGGATTCATATTAAAAGA

>CLEC4E-F_TRINITY_DN9366_c0_g1_i9
TAACTTTATTTGCAACTATTGTTTTTCAAGGCAATGATTTTTTTTTTTATTTTGACATCCAC
AAAAATAACCATAAATGAACGATGTGTACCATTAACTGAAATACCAAATGTAGTACAT
TATGTTGAATTAGTAGTAAACCGTTAAGAAAAAAAGATAAAGATGCCTTATTTGTCTTA
CCAAGCTGACTGTGTAGAAGTCAAACACTTTATAAAGGACAGTGCCAACATGTTTTGCA
TTATTATCCCTTACTTAGGAAAATCACAAAGAAATTAAGTGAATTTATGACAAGCTACA
TCTGGTTGGTTTTTCACAAATAAAATGAAACAAGGACTGGCAGTCTTCATCGTTCCATTT
TGACTGACAAGAATCCCACATGATCGCACAGTTTTCACTATTGGGATTGTTACCCCATG
CATTATTAGGTTCATTCTTGCACCAATGTCCAGATGATTTACTTTGAATTGTTCCATCTAC
CCAACGCCACACACCTTCCTCAATTTCATCAGTCAGTCCAATCCAGTAATCATTTTGGTT
GTTTCTTATAAAGAAAGTCTGTTCTTCAGCATTTGTGATCACCACTAATTCTGATGACAG
ATCTTGGCACATTCTCCTGGCAGTAGCCCAAGTTACAGACTTTTTGTAGTAGAAGTAAC
AGCTCAGCCTAAATTGCTTCCAGTCTGAAGGGCAAGGTTCAATCTGCTTTCTTTTAGAGA
GTTCTGTGAATTCAGTATTTTTTTGTCTGATCTGCCTTTGAAGGTTTTCCGTTTGCGTTAAC
ATGCTTTGAAAGCTGTGATTCAATAAGAGAAGGTCCTGGTTTGCTTGTTCTAGTTGTTTT
GACTTCTGTAAATATAGAATAGTCATGAAGATGCCAATTGCCACCAGGGATAAAATGA
GCATCAGGATGAATAACACCTTACAGTTCCACTTTGTTTTCTTCTTTGTGCTGTTAGGTTC
CTGCATGCCTTCTTTGGCACTTCGTAAAATTTCTGCTTGGGGATTGTTAGTTTGTTGCATA
TTAATTCTAGAATAAATGTTTTCATTTCTACATTGAAGCTCCTCATATGTTGTATTTTTTTC
CATGATCTTCAAAAATTTTAGAAACACCTGAAACTGACAGAGGAGCAGCGAAAGGCTG
TGTACCAGCATTTACAATGAAGAGGGAACCTGTGAGGCACGTTCCCCAGTAGAACAAA
TAAGTAGTGTTAAATCCAGAAGAATACGTAAAACAAACTAGTTCCTTAAAGAGTTCGTC
TTTTATTAAACAAATGCTGATTAAAAAAAGGTAGTTTAGCAC

>CLEC4E-G_TRINITY_DN383_c0_g1_i3
TCAACGAAAGGTGACAGCACTAATAACAAGACTTATTTATCAAATTTATTTTCAAGTGG
TGGTAGTTTGTGTCATCACTGAGCATCATGGAATACGAGAAGACATACCAGGAGCTTAA

GGTTGCAACTGAAGACACTTACTGTGAACTCAATCAACAACAAACCAAGAAACTCAGA
GCACAAAAGACAGCTCCAGAAACACAAAAATATGTGGCTAAGTATTGCAAACAACCA
AGAAAATTGTTAATCACCTCCATTATCACAGTGTTCATCCTGCTGGCAGTAATCATAGCT
GTGATTATTTGCAGTAAGATAATTCCTGAACCTGCTGTTTTAAAAGGCAATTCTTCAACT
TGTCTTTTAGATCATGCAAAGACGTTTCAAAACAGCTGTTATTACTTCTCAAACAGAAAT
TTAGAATGGAACGACAGTAAGAAATGGTGTGAAAACAGAAAAGCTCATTTAGTAATAA
TCAAAAGTGAAGAAGAGCAGAAATTCCTAAAAGACGAAGTTACTAATTATGGAAACA
CCTTTATACGAAAACCTAAAGGTCATTTTTTCTGGATTGGACTTACAGATGAAGTAAAG
GAAGGAGAGTGGCGCTGGATAGATGGCACTAACTGTGACTGCAATAGTAATAACAAAA
ATAAGGGATACTGGAGGGAAAATGAGCCAGACAACTGGAATAATTTAGAGCACTGTGC
TGCACTTGCTGAGAATGGTGACTGGATGGATGGGAAATGTAACTGGACTCTCAGGTGG
ATTTGTAAGATGGATGCTACGATTTTCTCTAGCTAATCAATTTTCTCAATGACAGTTGTTT
AAATGTGACACTGATGTTTGATCTAAACAACCATTGTAACAGTCTTGAACATGTTAATC
ATTCAATGATTATTTAATTATTATTTGGTTTGTTCATGAAAGACCCAAGTAATATTGAGC
ACATGGACAATAATTCTGCTGTTGATGACTTTCTCCATTATTTCATGTATATGTTTAGTGT
TATATGCCTATATTCTAATCTCTAATGGATTCATATTAAAAGA

>CLEC4M-A_TRINITY_DN1982_c0_g1_i1
ATGCATGATAGCCATGCTTGGATATAGTTTCAGTTCCAGTGAGGAAAAGGTTTCATTCA
TATTTACAAATACCTGGTTTACAAAATCTTCTTACATTTCTGCTTATACCAATGGTAATA
ATAATAATACTAGCCAAATCAGGGACATGAAAGCATTTCTGTAGGTCTTTGGTCTTCCC
AATGAATGCAAACCATTAAATAGAGTTATACAGGACAAAATGAAAATAGCACGTGAA
AGAAATGTTTATTTTCCCACTCAGCCTTTCTTTTCAAAATATAAAAGGAATTAAAGTTTT
TGTATTACTCTTAGTGAAAAAATAGTTTACTGTTTGACATTTAACATAATGCTCCATGGC
AATGCCTAGCCTTCACAGTCGGGGTCTGAGACTGAAGTCTTTGCAGCTCGCATTTATAA
ATAGCCTTTACAAAGAGCAAACAGAAGTCGGTGCCTCACAAATAAAGCGTAATACGCT
GGAACAGCGAGAATCACTCCATCTACCTTCAGATGTCATTAAGGCACAGTCTTCGTTAT
ACCAGTCATCAGGCTCACCGCTAACCCAAAATTGTTTTCTGTTGTAGTTGCATTCACAAA
AAGTGCCATCTACCCATTTCCACGTATCCTCTTTTTCGTTATCTGTAAGCCCAATCCAGT
ACCACAGTTCACGTTCATATCTGGGTTGCTTATTTAACTCTTTTGCTCTCTTATACAAAAA
GTCCTGCTCCTCAGTACTTTTGATCACCACTAAATCCGATGACCAGTCTTTACATGTACT
CCTTGAATTTGTCCAGCTTTCCCTAGACGCTGAGAAGTAGTAACAACTTTGTTTGAATAT
CTTCCAGCTTGCAGGGCAACATTCAACCTCCCTTCCTTGGTTTGATGATGCAGCAATTTT
GTTCAACATATTCAGTTTATTTTCTATTTCCTTATATTTCTCTTCTGTCCTGTTTTCTTGCTT
ATCCATTTGGTGTTGGATTGTGATGTTAATTTTTTTTTCAAGCTGAAACAGAAGGGCTTC
CTTACTTCTTTGGAGTTCTGTAATATTATCTTCTGTCTCATGGACCTTCATCTGTAGGTCT
GTCTGATTAAATGCCAAGATGGTGATTTTCTCTTCTGCTTCCTTAAATTTACTGTAAACA
CTGAAATTCAATGACTGCAACTCGTGATTTGCTGGCCCATATTGTTTTATTGGCTGTAAA
TGTAAAACAGCCACAGTTATGACCACTGTGAGTAGGACGATAATGACAAATACCATAA
AGAACGGCATCACTCTGCTTGTCAGTGTTTTCTTGTTTACATCCTTAAATTTCTGCGATCC
TTGCTTGTTTGTTTTCTCTAGCTGTGTGTTCACAACTGGAGTGCCATGTCGATTGAAGCTC
ACTTCAGCATATGTTATTTCGTCTTGCATGTTGGTTATTCCAAGTTCTTTTTGTTTTACTGC
TTTAATTTTATTTTGCCTTTCCTTTCAAAGCAAGTTCTATGTTTCCATATACTGACAGGTTT
TAGATGACTCTCTGTGTTTGCTGTGATCTCTTCTTTTGTCTTATTTCCCTTTTGCTGTCTCCT
GCTTACTGTCTATATTTTTCTCAAGTTCTTACTCATGTTTCCTGAACTTTTTTTTTAATCTAT
TTGCTAAATGTTATCTCACCCCACCTTCCTTCCATCCGTCTCTCTCTCTCTTGCTTC

>CLEC4M-B_TRINITY_DN1982_c0_g1_i4
ATGCATGATAGCCATGCTTGGATATAGTTTCAGTTCCAGTGAGGAAAAGGTTTCATTCA
TATTTACAAATACCTGGTTTACAAAATCTTCTTACATTTCTGCTTATACCAATGGTAATA
ATAATACTAGCCAAATCAGGGACATGAAAGCATTTCTGTAGGTCTTTGGTCTTCCCAAT

GAATGCAAACCATTAAATAGAGTTATACAGGACAAAATGAAAATAGCACGTGAAAGA
AATGTTTATTTTCCCACTCAGCCTTTCTTTTCAAAATATAAAAGGAATTAAAGTTTTTGTA
TTACTCTTAGTGAAAAAATAGTTTACTGTTTGACATTTAACATAATGCTCCATGGCAATG
CCTAGCCTTCACAGTCGGGGTCTGAGACTGAAGTCTTTGCAGCTCGCATTTATAAATAG
CCTTTACAAAGAGCAAACAGAAGTCGGTGCCTCACAAATAAAGCGTAATACGCTGGAA
CAGCGAGAATCACTCCATCTACCTTCAGATGTCATTAAGGCACAGTCTTCGTTATACCA
GTTATCAGGCTCACCACTGACCCAATATTGTTTTCTGTTGTAGTTGCATTCACAAAAAGT
GCCATCTACCCATTTCCACGTATCCTCTTTTTCGTTATCTGTAAGCCCAATCCAGTACCA
CAGTTCACGTTCATATCTGGGTTGCTTATTTAACTCTTTTGCTCTCTTATACAAAAAGTCC
TGCTCCTCAGTACTTTTGATCACCACTAAATCCGATGACCAGTCTTTACATGTACTCCTT
GAATTTGTCCAGCTTTCCCTAGACGCTGAGAAGTAGTAACAACTTTGTTTGAATATCTTC
CAGCTTGCAGGGCAACATTCAACCTCCCTTCGTAAAACAGCCACAGTTATGACCACTGT
GAGTAGGACGATAATGACAAATACCATAAAGAACGGCATCACTCTGCTTGTCAGTGTTT
TCTTGTTTACATCCTTAAATTTCTGCGATCCTTGCTTGTTTGTTTTCTCTAGCTGTGTGTTC
ACAACTGGAGTGCCATGTCGATTGAAGCTCACTTCAGCATATGTTATTTCGTCTTGCATG
TTGGTTATTCCAAGTTCTTTTTGTTTTACTGCTTTAATTTTATTTTGCCTTTCCTTTCAAAGC
AAGTTCTATGTTTCCATATACTGACAGGTTTTAGATGACTCTCTGTGTTTGCTGTGATCTC
TTCTTTTGTCTTATTTCCCTTTTGCTGTCTCCTGCTTACTGTCTATATTTTTCTCAAGTTCTT
ACTCATGTTTCCTGAACTTTTTTTTTAATCTATTTGCTAAATGTTATCTCACCCCACCTTCC
TTCCATCCGTCTCTCTCTCTCTTGCTTC

>CLEC19A-A_TRINITY_DN1706_c0_g2_i2
TATTAAATATCTTATTATCAAAAATTACAAAGTATATTTAGCACTTCAAATCATCAAATA
CAGAATGTAGGAATAAACATTTAAGAAAGAGAAATGCAGTACTAATAAAACAAGTAA
AATCCAACAGTGAAGCCAATAAGAAACAACAGGTATATTTGATTTTGTATAAAACTGT
AAATTTTTTAAAATCATCTGCTGAAAACATTTCTTGCGATGTCCATGTAAGACTTCTCCT
TCATATTAGAATATTGGTGCGTATACAGAGCATCTTCGAAGGTCATATTTGCAGACAAA
AGGGAAAGACCATCTGCTACTGTAGTCATTCCAGGTAATAGTACCATTCTGGAAATACC
ATGACCCAACATAATCTTCATTGCCAAGATTGTCTGGTTGGGATTTGCCAAAACGTAGG
AAGTTCACTGGACTTCCATCAGTCCACATAAAAACACCCTCTACAAAAGTGTCATGTAA
TCCAATCCAGTAAGCCTGTCCTTTTATGTAATCATTGGGTCTACCCATTAGTTCATAAAT
GTAGTTATTTTCTTCAAGACTGTGGATGGATGCCAAATGAGCTCCGTAGTAGTTTCTTTG
GCAGTAGGCCTCAGCATTAACCCATGTCATTTTTATTTGTATGGGACGAAAGCATGAAT
TCTTGTACTGGAACCATCCAAATGAGCAGTCCCCCTGATAACAGAGACAGCCATCTGCA
GTGTCACCACCAGGAATAAAGGCAGCCAGGAGTACAGACATCAGTATCAGGTAATTCA
TCTTTACAATGAATACGTCAAGTATGATGAACTTTTTTGGCAA

>CLEC19A-B_TRINITY_DN1706_c0_g1_i1
TTATAACTTTGCCAAAAAAGTTCATCATACTTGACGTATTCATTGTAAAGATGAATTACC
TGATACTGATGTCTGTACTCCTGGCTGCCTTTATTCCTGGTGGTGACACTGCAGATGGCT
GTCTCTGTTATCAGGGGGACTGCTCATTTGGATGGTTCCAGTACAAGAATTCATGCTTTC
GTCCCATACAAATAAAAATGACATGGGTTAATGCTGAGGCCTACTGCCAAAGAAACTA
CTACGGAGCTCATTTGGCATCCATCCACAGTCTTGAAGAAAATAACTACATTTATGAAC
TAATGGGTAGACCCAATGATTACATAAAAGGACAGGCTTACTGGATTGGATTACATGA
CACTTTTGTAGAGGGTGTTTTTATGTGGACTGATGGAAGTCCAGTGAACTTCCTACGTTT
TGGCAAATCCCAACCAGACAATCTTGGCAATGAAGATTATGTTGGGTCATGGTATTTCC
AGAATGGTACTATTACCTGGAATGACTACAATAGCATATGGTCTTTCCCTTTTGTCTGCA
AATATGACCTTCGAAGATGCTATGTCTACAGACCAATATTCTAATATGAAAGAGAAGTC
TTACATGGACATCGCAAGAAATGTTTTCAGTAGATGATTTGAAAAAAATTACAGCTTTA
TACAAAATCAAGTATACCTGTTGTTTCTTATTACATGTGATTTTATGGTTGGGTACAACC

TTTTTATTATTACTGCATTTCTCTTTCTTATATGTTTATCCCTACATTCTGCATTTGATGATT
TGAAGCTTTACATATGTACTGTAATTTTTGATGAAAG

>CLEC19A-C_TRINITY_DN14306_c0_g2_i2
AAAATATTGCACACCATTGTGTTCATTAGAAAGATGAATTACCTGATACTGATGTCAGC
TATCCTGGTCATCTTTATTCCTCAGGGTGACATGCAAATCAACCATTGCCTCTGTGTTCG
TGGGATCTGCCCATATGGATGGTACCAGTACACAGATAATTGCTACAAAGTTATAACTA
CAAAAATGAGTTGGGTGAATGCTGAGGCCTTCTGCCAGAGTAGCTACCTTGGGGCTCAT
TTGGCATCCATCCACAGTGCTGAAGAAAATGATTACATTTTCACACTGACTGGGAACCT
ATCTAATTACCAAACAGGAAATGCATACTGGATTGGAGCCCATGACACTATTGTAGAA
GGCACCTACATGTGGACAGATGGAAGTGTGACAGACTACTTGCATTTTGGAACTGGTCA
GCCAGATAACCTTGGCAATGAAGATTATATTGGATCGTGGCATTTTCAAAATAATGCTA
TTACCTGGAATGACTACCCAGAATCTTTTTCCTTCCCTTTTGTCTGCAGGATCAGCCTAA
ATAGATGCTGTCGTTCTTGAATGAAGAGTTGCAGACTGGCAGCCACGTATGACAAGTCT
CCTGAGAACATCACAAATGTTATGGCTCCCATCCAAGGAATTATATAATTTCTTTTCAA
ATTATTTGCATCTATTGTTTGTACGATTATCCTATTCTTTTATTAAAGGTGTTTTTTTGGT
ACTATTCAATTTTATGACAATGGCCTGCTGAAATGAAATCCACTAGCTGTATCACTTCA
ACAGACATTTATTCAGGTATATGAGTCAATGAACTCCTCTGGCCATCTTTCACTTGGTAT
GCATTTTGTCAGTCCTCAAAATAATAAAAAGGAATTTGAAGCTCAACCCTCAATAATAC
CCTCTAAATACAGATTTTTTTTTAACTCAGAAAGTATAGTCAGTGTGAGTGAGCAAAGG
AGTTAACAGGCTTGTGCATTTGATTAAATGTCTACCAAAATCAAACTGTCAGTAGATGT
AAATGTAACATCCGCTTAAGAATTGCAGAACCATATTTTCTTTTTCTGCTCATGTTGTATT
GCTATTTTCTTGCAGGTATGTATGATCTGCTCTGATTGCATTGTGGAGGTTCTAGGCATTC
CATTCTATACTGTCATTATTGATATAACGTTGTCATTTGGGCTAAGTTGCTGAACAAATG
GTGTATGCTACAGGTCTTAATAATAATACAATTCA

>CLEC16A-A_TRINITY_DN2288_c0_g1_i1
GATTAGCGATGGCTGAAATTTTGGATATGTGGGAATGGTAATGTCACTTATTAAAGGAA
GATTTGGGTACTTCGAACGTGGTTGTTGGTTTTCAAAATGGGTAACGGAGACTAAAATC
CATTCAGGATGTTTGGCCGTTCTAGAAGCTGGATGGTTGGCGGTCCAGGAAGGTCTTCA
AAGAGCATTCACTCTTTGGACCACTTAAAAAATATGCATCACCTCTTAACTAAAAACAC
TACTGTCACTGATCACAACCGTGACCTGCTAGTAGAAACCATCCGTTCTATCACTGAAA
TTCTCATTTGGGGTGACCAGAATGACAGCTCAGTGTTTGATTTCTTTTTGGAGAAAAATA
TGTTTGCCTTCTTCTTGAATATTCTTCGGCAGAAATCTGGGCGCTATGTCTGTGTGCAGCT
GCTTCAGACCCTGAATATTTTGTTTGAGAACATCAGCCACGAAACATCCCTTTATTACTT
ACTGTCCAACAACCATGTGAATTCAATTATCGTTCACAAGTTTGACTTCTCTGATGAGGA
GATTATGGCATACTACATATCATTCCTGAAGACACTGTCATTGAAGCTCAACAACCATA
CAGTACACTTTTTTTACAATGAGCACACAAATGATTTTGCATTGTATACAGAGGCAATC
AAATTTTTTAATCATCCAGAAAGCATGGTCAGAATTGCTGTCAGGACCATCACTCTTAA
TGTTTATAAAGTGAACAACCAGCATATGCTACATTATATTAGAGACAAAACTGCTGCCC
CCTATTTCTCCAATCTGGTCTGGTTCATTGGGAGCCATGTGATAGAACTGGACAACTGTG
TCCAGACTGATGAAGAACACGCAAACAAGAGCAAGCTGAGTGACCTTGTTGCAGAGCA
CCTCGACCACTTGCATTACCTAAATGACATTCTGATTATCAACTGTGAATTTCTAAATGA
TGTGCTTACAGATCACCTGCTGAACAGACTGTTCCTGCCTTTGTATGTCTACTCCTTAGT
AACTCATGAAAAGTGTGGAGAGCTTCCAAAAATCAGCGCGCAGGTGTCACTCTATCTC
CTTTCACAGGTCTTTCTGATCATACACTACTCACCTTTGGTGAACTCCTTGGCTGATGTTA
TTTTGAACGGTGACCTTTCTGTGTTTTCTGCACAGACAGAGCAAGATGTACAGAAGAGC
ATGGCAAAGTCCAATATACGATGCTTCACAAAGCCTGCAGAGAGTCTTGAGAAATCAC
TTGAGATCAGTAAGCAGAGGGGGAGAAAAAAAGCACAGAAAAGGCCAAATTATAAG
AATGTGGGTGAGGAGGAGGAAGAGGAAAAACCTCCAGATGAAACTCTGGAAGATCCT
GACAAGGCAAAAGGTACAGACGGAGCTTCAAAAAGCAACAGGACTACTTTGGAGACA

GAAGAAATAGAGATGGTAATTATGGAGAGGTGTAAAATATCAAAAGCTGTGGTAATGG
AACAGAATACTACAGATGAAGAGAAGAGTGCTGCTGCAGCAGCTGGAATAGAGACTT
CACAAAACAGGCCATTTTTGGATGTGGTATATAATGCTCTGGACTGCTCGGGAGATGAT
TATTATGCATTGTTTGTCCTTTGTCTGCTCTATGCAGTGTCCCACAACAGAGGTATTGATT
CTACTCTGCTAGAAAAAATCCAGCTTCCATTTCGGCAAACAGCTGAGAAGACAGTATAT
AGTCATGTTCTTGCTGAGAGGCTAATCAGGATAACGATCAATGCTGCCCAGCCAGACA
GCAAGATTCGACTTGCAACTTTGGAACTTAGTTGTTTGCTTCTGAAACAGCTTGTAATTT
CAGACAGCGGCTGTGTTATAAAGGACATACACCTGGCTTGCCTGGAGGGGGAAGAAAT
TTTTCTTGATGTGTTTGAAGATGAATACAGAAGCATGATGAACAAACCACTGAATGTAG
AGTACCTGATGATGGATGCTTCCATTCTGCTGCCCCCCACTGGGACTCCGCTGACTGGTA
TTGATTTTGTTAAGAGACTTCCCTGTGGAGATGTCGAGCGAACACGAAGAGCAATAAG
AGTTTTTTTCATGTTGCGAGCTTTATCACTGGAACTGAGAGGAGAACTGGAGACTCAGC
TGCCCTTGACAAGGGAGGAAGATCTGATTAAAACTGAAGATGTTTTGGATCTTAATAAC
AGTGACTTGATTGCCTGCACAGTGATTGCAAAGGATGGAGGACAAGCTCAGCGATTTCT
GGCTGTTGATATTTACCAGATGAGTTTAGTTGAACCCGAGACCAAAAGACTTGGCTGGG
GTGTGGTGAAGTTTGCTGGACTTCTGCAGGATATGCAGGTCACTGGGGTGGAAGATGAC
AGCCGTGCGCTAAATATCACAATTCACAAGCCCACATCAAATCCTCATGCCAAACCATT
TCCCATTTTACAGGCTACATTTATCTTTTCTGATCATATCCGCTGCATAATTGCAAAGCA
GAGACTGGCAAAGGGCCGCATCCAGGCACGGCGTATGAAAATGCAGAGAATAGCAGC
ATTGCTAGATCTTCCTGTTCAGCCATCTCCTGAGGTAATGGGCTTTGGACATGTTACAAG
TGCAGCTACCCAGCATCTGCCTTTTCGATTTTATGACCAGTCAAGACGTGGATTAAGTG
ACAGTGCAGTTCAGCGTTCTGTGTTTGCTTCTGTTGACAAAGTTCCAGGTTTTGCTGTAG
CCCAGTGTGTAAACCAGCATACTTCTGCACCAGCATCCTCACCATCCCCTCCTTCCAGC
AGTAGCCCCAATGGCAGTGGGAGCACTGGTCATTGTGATTCAGTCACAGCTAGCACAA
CATCCACTCCTGCAGCCCAAAGTCCATCAGACGATCCAGCATCTTTGGAAGAGTCATCG
CTGAATCTGCCTGCGCAGGTGGAGTTTGTTGATGAGACTAGCTGTAGCACTCCAGGCCC
CAGCAAGAACTTCCAGAGGAACTCTGAGTCTGAACCAGCTAACCTGGCCCCAAGCCTT
ACCCCTTCTCAACAGCCAACCATATCATTACTGTCAGATGACAATACAGATGCACGCAG
TGTGGAGTCCTTGACACTTGTCCCACCTGCTGATCCACATTGCATCTTAAGTTTTAGCAA
CACTGTACAGTCCTCAGTGCTGTCTGAGTCAGCATTAACCAATGAGGGGTATCATGCTG
AGACTTTTGAACCAGAAACAAGTACAGAAGTGTGAACAGAACCCATTAGACTTTTTTCT
CTTCTTGGAATGGGAGACTCTGTGAACTTTCAATGACTTTTGATGCAAAACACTGCAAT
ATACCACGTTGTTCATGGTAGAAGTGAAATGCAAGCAACTGTCTTTTAATGTCTATGGG
AGAGATGTTGAAGGAAGACTGATGAAGCTGAATTTCTTCACTGATTAGTCCATATAATA
AAAGGAAAGTGCAGTCTCCCTGCTGATTGAACAAAATTTCCAGGTGTTATTTATTCACA
CAGCTTAGTTCTGCTATTTTCACAGTGTTGAACAATGACTTGAAGGTGGACAGCTGAAG
GAGGTCTTCTTTTCTTCCCCATTTTTATTGTCTGCCGCCAAGTTTTGTTAGACTAGGCTTTT
GTATCTGCAGGTTGTCAGTCACTACAAGTCCATGTGATGTGTGAAAATACCATTTGTCAT
CTGATCTAATGGAGTCAGGATCGTAGTTTTGAAATAATGGCCTGCCTAAATAAAATCAT
TTACCATGATCTTCCAAACAAGTTGTATCTTGTACACTAAAATTGCACTGGATGTTTAGG
GACCAGTATAATTTTGTGAAACATACGTTCTTATTTATTCCTTTTGTTGTAGTTATAAATA
TGGGTTATGTTAAAAATGTTAAAGTGGCCTACAGTAATATAGCAGATGCATTATGCCAT
GTGTTTGCATTCAGTTAGATGCATTTTCCCTGTATTTAGTGCAGATTTAG

>CLEC16A-B_TRINITY_DN2288_c0_g2_i2
CTAAATCTGCACTAAATACAGGGAAAATGCATCTAACTGAATGCAAACACATGGCATA
ATGCATCTGCTATATTACTGTAGGCCACTTTAACATTTTTAACATAACCCATATTTATAA
CTACAACAAAAGGAATAAATAAGAACGTATGTTTCACAAAATTATACTGGTCCCTAAA
CATCCAGTGCAATTTTAGTGTACAAGATACAACTTGTTTGGAAGATCATGGTAAATGAT
TTTATTTAGGCAGGCCATTATTTCAAAACTACGATCCTGACTCCATTAGATCAGATGACA
AATGGTATTTTCACACATCACATGGACTTGTAGTGACTGACAACCTGCAGATACAAAAG

CCTAGTCTAACAAAACTTGGCGGCAGACAATAAAAATGGGGAAGAAAAGAAGACCTC
CTTCAGCTGTCCACCTTCAAGTCATTGTTCAACACTGTGAAAATAGCAGAACTAAGCTG
TGTGAATAAATAACACCTGGAAATTTTGTTCAATCAGCAGGGAGACTGCACTTTCCTTTT
ATTATATGGACTAATCAGTGAAGAAATTCAGCTTCATCAGTCTTCCTTCAACATCTCTCC
CATAGACATTAAAAGACAGTTGCTTGCATTTCACTTCTACCATGAACAACGTGGTATAT
TGCAGTGTTTTGCATCAAAAGTCATTGAAAGTTCACAGAGTCTCCCATTCCAAGAAGAG
AAAAAAGTCTAATGGGTTCTGTTCACACTTCTGTACTTGTTTCTGGTTCAAAAGTCTCAG
CATGATACCCCTCATTGGTTAATGCTGACTCAGACAGCACTGAGGACTGTACAGTGTTG
CTAAAACTTAAGATGCAATGTGGATCAGCAGGTGGGACAAGTGTCAAGGACTCCACAC
TGCGTGCATCTGTATTGTCATCTGACAGTAATGATATGGTTGGCTGTTGAGAAGGGGTA
AGGCTTGGGGCCAGGTTAGCTGGTTCAGACTCAGAGTTCCTCTGGAAGTTCTTGCTGGG
GCCTGGAGTGCTACAGCTAGTCTCATCAACAAACTCCACCTGCGCAGGCAGATTCAGC
GATGACTCTTCCAAAGATGCTGGATCGTCTGTTTCCTGCTTTGTAAGAGCTGCTGCTGCT
TGAAGACCTAAACCCAGTGTCCACCATGACTGTCAGCTTCCGACAGACTAAAAACTCG
CCGCCGTCCTTTCCTGCCAGACCTTACTGATGGACTTTGGGCTGCAGGAGTGGATGTTGT
GCTAGCTGTGACTGAATCACAATGACCAGTGCTCCCACTGCCATTGGGGCTACTGCTGG
AAGGAGGGGATGGTGAGGATGCTGGTGCAGAAGTATGCTGGTTTACACACTGGGCTAC
AGCAAAACCTGGAACTTTGTCAACAGAAGCAAACACAGAACGCTGAACTGCACTGTCA
CTTAATCCACGTCTTGACTGGTCATAAAATCGAAAAGGCAGATGCTGGGTAGCTGCACT
TGTAACATGTCCAAAGCCCATTACCTCAGGAGATGGCTGAACAGGAAGATCTAGCAAT
GCTGCTATTCTCTGCATTTTCATACGCCGTGCCTGGATGCGGCCCTTTGCCAGTCTCTGCT
TTGCAATTATGCAGCGGATATGATCAGAAAAGATAAATGTAGCCTGTAAAATGGGAAA
TGGTTTGGCATGAGGATTTGATGTGGGCTTGTGAATTGTGATATTTAGCGCACGGCTGTC
ATCTTCCACCCCAGTGACCTGCATATCCTGCAGAAGTCCAGCAAACTTCACCACACCCC
AGCCAAGTCTTTTGGTCTCGGGTTCAACTAAACTCATCTGGTAAATATCAACAGCCAGA
AATCGCTGAGCTTGTCCTCCATCCTTTGCAATCACTGTGCAGGCAATCAAGTCACTGTTA
TTAAGATCCAAAACATCTTCAGTTTTAATCAGATCTTCCTCCCTTGTCAAGGGCAGCTGA
GTCTCCAGTTCTCCTCTCAGTTCCAGTGATAAAGCTCGCAACATGAAAAAAACTCTTATT
GCTCTTCGTGTTCGCTCGACATCTCCACAGGGAAGTCTCTTAACAAAATCAATACCAGT
CAGCGGAGTCCCAGTGGGGGGCAGCAGAATGGAAGCATCCATCATCAGGTACTCTACA
TTCAGTGGTTTGTTCATCATGCTTCTGTATTCATCTTCAAACACATCAAGAAAAATTTCTT
CCCCCTTATAAAAGCGTCGTAAGAGATGGACACTTTCTTCCCTTGCACCCTCCAGGCAA
GCCAGGTGTATGTCCTTTATAACACAGCCGCTGTCTGAAATTACAAGCTGTTTCAGAAG
CAAACAACTAAGTTCCAAAGTTGCAAGTCGAATCTTGCTGTCTGGCTGGGCAGCATTGA
TCGTTATCCTGATTAGCCTCTCAGCAAGAACATGACTATATACTGTCTTCTCAGCTGTTT
GCCGAAATGGAAGCTGGATTTTTTCTAGCAGAGTAGAATCAATACCTCTGTTGTGGGAC
ACTGCATAGAGCAGACAAAGGACAAACAATGCATAATAATCATCTCCCGAGCAGTCCA
GAGCATTATATACCACATCCAAAAATGGCCTGTTTTGTGAAGTCTCTATTCCAGCTGCTG
CAGCAGCACTCTTCTCTTCATCTGTAGTATTCTGTTCCATTACCACAGCTTTTGATATTTT
ACACCTCTCCATAATTACCATCTCTATTTCTTCTGTCTCCAAAGTAGTCCTGTTGCTTTTT
GAAGCTCCGTCTGTACCTTTTGCCTTGTCAGGATCTTCCAGAGTTTCATCTGGAGGTTTTT
CCTCTTCCTCCTCCTCACCCACATTCTTATAATTTGGCCTTTTCTGTGCTTTTTTTCTCCCC
CTCTGCTTACTGATCTCAAGTGATTTCTCAAGACTCTCTGCAGGCTTTGTGAAGCATCGT
ATATTGGACTTTGCCATGCTCTTCTGTACATCTTGCTCTGTCTGTGCAGAAAACACAGAA
AGGTCACCGTTCAAAATAACATCAGCCAAGGAGTTCACCAAAGGTGAGTAGTGTATGA
TCAGAAAGACCTGTGAAAGGAGATAGAGTGACACCTGCGCGCTGATTTTTGGAAGCTC
TCCACACTTTTCATGAGTTACTAAGGAGTAGACATACAAAGGCAGGAACAGTCTGTTCA
GCAGGTGATCTGTAAGCACATCATTTAGAAATTCACAGTTGATAATCAGAATGTCATTT
AGGTAATGCAAGTGGTCGAGGTGCTCTGCAACAAGGTCACTCAGCTTGCTCTTGTTTGC
GTGTTCTTCATCAGTCTGGACACAGTTGTCCAGTTCTATCACATGGCTCCCAATGAACCA
GACCAGATTGGAGAAATAGGGGGCAGCAGTTTTGTCTCTAATATAATGTAGCATATGCT

GGTTGTTCACTTTATAAACATTAAGAGTGATGGTCCTGACAGCAATTCTGACCATGCTTT
CTGGATGATTAAAAAATTTGATTGCCTCTGTATACAATGCAAAATCATTTGTGTGCTCAT
TGTAAAAAAAGTGTACTGTATGGTTGTTGAGCTTCAATGACAGTGTCTTCAGGAATGAT
ATGTAGTATGCCATAATCTCCTCATCAGAGAAGTCAAACTTGTGAACGATAATTGAATT
CACATGGTTGTTGGACAGTAAGTAATAAAGGGATGTTTCGTGGCTGATGTTCTCAAACA
AAATATTCAGGGTCTGAAGCAGCTGCACACAGACATAGCGCCCAGATTTCTGCCGAAG
AATATTCAAGAAGAAGGCAAACATATTTTTCTCCAAAAAGAAATCAAACACTGAGCTG
TCATTCTGGTCACCCCAAATGAGAATTTCAGTGATAGAACGGATGGTTTCTACTAGCAG
GTCACGGTTGTGATCAGTGACAGTAGTGTTTTTAGTTAAGAGGTGATGCATATTTTTTAA
GTGGTCCAAAGAGTGAATGCTCTTTGAAGACCTTCCTGGACCGCCAACCATCCAGCTTC
TAGAACGGCCAAACATCCTGAATGGATTTTAGTCTCCGTTACCCATTTTGAAAACCAAC
AACCACGTTCGAAGTACCCAAATCTTCCTTTAATAAGTGACATTACCATTCCCACATAT
CCAAAATTTCAGCCATCGCTAATC

>CLEC16A-C_TRINITY_DN2288_c0_g1_i3
GATTAGCGATGGCTGAAATTTTGGATATGTGGGAATGGTAATGTCACTTATTAAAGGAA
GATTTGGGTACTTCGAACGTGGTTGTTGGTTTTCAAAATGGGTAACGGAGACTAAAATC
CATTCAGGATGTTTGGCCGTTCTAGAAGCTGGATGGTTGGCGGTCCAGGAAGGTCTTCA
AAGAGCATTCACTCTTTGGACCACTTAAAAAATATGCATCACCTCTTAACTAAAAACAC
TACTGTCACTGATCACAACCGTGACCTGCTAGTAGAAACCATCCGTTCTATCACTGAAA
TTCTCATTTGGGGTGACCAGAATGACAGCTCAGTGTTTGATTTCTTTTTGGAGAAAAATA
TGTTTGCCTTCTTCTTGAATATTCTTCGGCAGAAATCTGGGCGCTATGTCTGTGTGCAGCT
GCTTCAGACCCTGAATATTTTGTTTGAGAACATCAGCCACGAAACATCCCTTTATTACTT
ACTGTCCAACAACCATGTGAATTCAATTATCGTTCACAAGTTTGACTTCTCTGATGAGGA
GATTATGGCATACTACATATCATTCCTGAAGACACTGTCATTGAAGCTCAACAACCATA
CAGTACACTTTTTTTACAATGAGCACACAAATGATTTTGCATTGTATACAGAGGCAATC
AAATTTTTTAATCATCCAGAAAGCATGGTCAGAATTGCTGTCAGGACCATCACTCTTAA
TGTTTATAAAGTGAACAACCAGCATATGCTACATTATATTAGAGACAAAACTGCTGCCC
CCTATTTCTCCAATCTGGTCTGGTTCATTGGGAGCCATGTGATAGAACTGGACAACTGTG
TCCAGACTGATGAAGAACACGCAAACAAGAGCAAGCTGAGTGACCTTGTTGCAGAGCA
CCTCGACCACTTGCATTACCTAAATGACATTCTGATTATCAACTGTGAATTTCTAAATGA
TGTGCTTACAGATCACCTGCTGAACAGACTGTTCCTGCCTTTGTATGTCTACTCCTTAGT
AACTCATGAAAAGTGTGGAGAGCTTCCAAAAATCAGCGCGCAGGTGTCACTCTATCTC
CTTTCACAGGTCTTTCTGATCATACACTACTCACCTTTGGTGAACTCCTTGGCTGATGTTA
TTTTGAACGGTGACCTTTCTGTGTTTCTGCACAGACAGAGCAAGATGTACAGAAGAGC
ATGGCAAAGTCCAATATACGATGCTTCACAAAGCCTGCAGAGAGTCTTGAGAAATCAC
TTGAGATCAGTAAGCAGAGGGGGAGAAAAAAAGCACAGAAAAGGCCAAATTATAAG
AATGTGGGTGAGGAGGAGGAAGAGGAAAAACCTCCAGATGAAACTCTGGAAGATCCT
GACAAGGCAAAAGGTACAGACGGAGCTTCAAAAAGCAACAGGACTACTTTGGAGACA
GAAGAAATAGAGATGGTAATTATGGAGAGGTGTAAAATATCAAAAGCTGTGGTAATGG
AACAGAATACTACAGATGAAGAGAAGAGTGCTGCTGCAGCAGCTGGAATAGAGACTT
CACAAAACAGGCCATTTTTGGATGTGGTATATAATGCTCTGGACTGCTCGGGAGATGAT
TATTATGCATTGTTTGTCCTTTGTCTGCTCTATGCAGTGTCCCACAACAGAGGTATTGATT
CTACTCTGCTAGAAAAAATCCAGCTTCCATTTCGGCAAACAGCTGAGAAGACAGTATAT
AGTCATGTTCTTGCTGAGAGGCTAATCAGGATAACGATCAATGCTGCCCAGCCAGACA
GCAAGATTCGACTTGCAACTTTGGAACTTAGTTGTTTGCTTCTGAAACAGCTTGTAATTT
CAGACAGCGGCTGTGTTATAAAGGACATACACCTGGCTTGCCTGGAGGGGGAAGAAAT
TTTTCTTGATGTGTTTGAAGATGAATACAGAAGCATGATGAACAAACCACTGAATGTAG
AGTACCTGATGATGGATGCTTCCATTCTGCTGCCCCCCACTGGGACTCCGCTGACTGGTA
TTGATTTTGTTAAGAGACTTCCCTGTGGAGATGTCGAGCGAACACGAAGAGCAATAAG
AGTTTTTTTCATGTTGCGAGCTTTATCACTGGAACTGAGAGGAGAACTGGAGACTCAG

CTGCCCTTGACAAGGGAGGAAGATCTGATTAAAACTGAAGATGTTTTGGATCTTAATAA
CAGTGACTTGATTGCCTGCACAGTGATTGCAAAGGATGGAGGACAAGCTCAGCGATTTC
TGGCTGTTGATATTTACCAGATGAGTTTAGTTGAACCCGAGACCAAAAGACTTGGCTGG
GGTGTGGTGAAGTTTGCTGGACTTCTGCAGGATATGCAGGTCACTGGGGTGGAAGATGA
CAGCCGTGCGCTAAATATCACAATTCACAAGCCCACATCAAATCCTCATGCCAAACCA
TTTCCCATTTTACAGGCTACATTTATCTTTTCTGATCATATCCGCTGCATAATTGCAAAGC
AGAGACTGGCAAAGGGCCGCATCCAGGCACGGCGTATGAAAATGCAGAGAATAGCAG
CATTGCTAGATCTTCCTGTTCAGCCATCTCCTGAGGTAATGGGCTTTGGACATGTTACAA
GTGCAGCTACCCAGCATCTGCCTTTTCGATTTTATGACCAGTCAAGACGTGGATTAAGT
GACAGTGCAGTTCAGCGTTCTGTGTTTGCTTCTGTTGACAAAGTTCCAGGTTTTGCTGTA
GCCCAGTGTGTAAACCAGCATACTTCTGCACCAGCATCCTCACCATCCCCTCCTTCCAG
CAGTAGCCCCAATGGCAGTGGGAGCACTGGTCATTGTGATTCAGTCACAGCTAGCACA
ACATCCACTCCTGCAGCCCAAAGTCCATCAGTAAGGTCTGGCAGGAAAGGACGGCGGC
GAGTTTTTAGTCTGTCGGAAGCTGACAGTCATGGTGGACACTGGGTTTAGGTCTTCAAG
CAGCAGCAGCTCTTACAAAGCAGGAAACAGACGATCCAGCATCTTTGGAAGAGTCATC
GCTGAATCTGCCTGCGCAGGTGGAGTTTGTTGATGAGACTAGCTGTAGCACTCCAGGCC
CCAGCAAGAACTTCCAGAGGAACTCTGAGTCTGAACCAGCTAACCTGGCCCCAAGCCT
TACCCCTTCTCAACAGCCAACCATATCATTACTGTCAGATGACAATACAGATGCACGCA
GTGTGGAGTCCTTGACACTTGTCCCACCTGCTGATCCACATTGCATCTTAAGTTTTAGCA
ACACTGTACAGTCCTCAGTGCTGTCTGAGTCAGCATTAACCAATGAGGGGTATCATGCT
GAGACTTTTGAACCAGAAACAAGTACAGAAGTGTGAACAGAACCCATTAGACTTTTTTC
TCTTCTTGGAATGGGAGACTCTGTGAACTTTCAATGACTTTTGATGCAAAACACTGCAAT
ATACCACGTTGTTCATGGTAGAAGTGAAATGCAAGCAACTGTCTTTTAATGTCTATGGG
AGAGATGTTGAAGGAAGACTGATGAAGCTGAATTTCTTCACTGATTAGTCCATATAATA
AAAGGAAAGTGCAGTCTCCCTGCTGATTGAACAAAATTTCCAGGTGTTATTTATTCACA
CAGCTTAGTTCTGCTATTTTCACAGTGTTGAACAATGACTTGAAGGTGGACAGCTGAAG
GAGGTCTTCTTTTCTTCCCCATTTTTATTGTCTGCCGCCAAGTTTTGTTAGACTAGGCTTTT
GTATCTGCAGGTTGTCAGTCACTACAAGTCCATGTGATGTGTGAAAATACCATTTGTCAT
CTGATCTAATGGAGTCAGGATCGTAGTTTTGAAATAATGGCCTGCCTAAATAAAATCAT
TTACCATGATCTTCCAAACAAGTTGTATCTTGTACACTAAAATTGCACTGGATGTTTAGG
GACCAGTATAATTTTGTGAAACATACGTTCTTATTTATTCCTTTTGTTGTAGTTATAAATA
TGGGTTATGTTAAAAATGTTAAAGTGGCCTACAGTAATATAGCAGATGCATTATGCCAT
GTGTTTGCATTCAGTTAGATGCATTTTCCCTGTATTTAGTGCAGATTTAG

>KLRG1_TRINITY_DN6253_c0_g1_i1
TTTTTCAGAGTAAGAATTGTGCTTTTTTTGTAACATTTATTTTTTTAGACAGTCCTACAATT
GTAACTGTAGTCCACTTTTAATGCAAGCTGCTAAATGCATCCTTTGTACATATGAAGTTG
AAGTCTTCTTCACACGATGAGGCTGAAAGTCCATGGGAGTTAATGACTAGACATCTCTG
TCCCACATAATTACTTCTCACAGGAAAAATGGTGGTATTCAGAGGAATTCCATCTTCCC
AAATCCACATGTCATTTTGTTTTGTGATCCCAATCCAGTGATATGTTTCTTTTGCTACTTT
GTTTGCAAAATTCATTGCTTCCTTTTGTTGTTCCTTAAATATCGATGAATGATGTGCTGAA
CAAAATTCTTTGGAAGAAGTCCAGTTCTTCTTTTTATCAGAAAATAGATAACATTTACTA
TGGTATCCAATCCATTTTTCTTGGCATACGAAGCACTTTCCTGTACCATTGCTTTCTGAAT
CTTTGCACTTCTGAGTTATCATCTTCCATTCAACTAACAGAAGAGTGAGGATTGACACTG
CCAACAAAAGGCACAATGTGGAAATGAGGAACATAATGCAACAACAATTGAAATTAG
ATGATTTAGGATCTTTCTCATTTCCAGAGTGCTGTTTTTGGTGTTCCAGTGTGCTGGGCTC
CTTGACTGTACCTCTGGCTGTTCTTGTGGTGGCTTTCTCATTTCCAGAGTGCTGTTTTTGGT
GTTCCAGTGTGCTGGGCTCCTTGACTGTACCTCTGGCTGTTCTTGTGGTGGCTCTTTCTTT
AGCTGTTGGCTCAATTTTTTTGAGTAAAACTGAATTAATGTATATATTTGCATCTTTCTTC
CTCACATCTTGTACAGCTTGATTATTCAGATTTTCAGCTTTTGTTTGTTGTTTAAATTCAG
ATATGTTGCAATAGATAACTCTGTTTTGTCCTTTCATGTTTTGTGAGCTTTCTTCTTCCAA

AGGTCCTGCTGTAAACTATGTTACTTTTTAGCAGATAAGATGTATTTGTTTGTCGCTTTG
ATTTTCTTCCAGGTTGCTGAAGGTTCTACTTTCTGTTCTGCATTATTAGCATTAACTTTAC
ATTGCTAGTGACAGATGGTCAGTTCTATCCCCATA

>SELE-A_TRINITY_DN68820_c1_g1_i11 len=2308 path=[3:0-165 8:166-231 10:232-232 12:233-912 13:913-942 15:943-2307]
CTGGGATGAAACTGCAGTCGGCGGTTTCAGGGACATTTTGTAGCTGTTCGTTTCCAAAC
AGCTACTCGTTCAGCTGATCGAGGGGAACGCAGAGGGACTGATCAGCTGATTTTCCATG
CCACTTCCACCAGGGAACTTTCTTTACTACGTTGTTAGTCAGCAGCTCGACCTGAAGAG
GGAGCTGCTGACTTAAGTCCTGGGAGAATGCCACATTTGTCTCTGTGGCTTTTTTTAAAT
CATGCAGTTCTCCCAGGCAAGTGAAGATATTTAACCTCTCCACCAGGAAGAAACTGCA
GTCGGCAGTTTCTGGGTGATACTAGCTGTTTTTCAAAGAGCTACGCATTTCAAGCTGATT
TAGGGGAATGTGAACAGACTGATCAGCTGTTTCTTGGTGCCACTTCCACCAGGGAAACT
TTATTCACCTAGTAATAGTTACCACGCAAAAAAGACAAAGCAATGCAGAACCCTGTGC
GAATGAAGGCTTTTATTCGCAACGGATACTTAATGACATTTGATGAAGCTAAAGAATAC
TGTCAAACATACTACGATGGCCTGGCAGAGATCTATCAAGAATCAGAGAATGCTCAAA
TACCAGAATTTATTCATGAGAGACAGTGCTGGATAGGTACTGAAAACTGTCAGAGGGT
CTTTGCCACAAATTATACTGAATGGTGCTACAGCAGAAGTAAACGCAATGTCAGCTTTT
TACCCTGGAAGAAAACAAAACCCGAATACAACCATGTGCAAGGGACATATGCCTGTGT
CTTTATTAAAGATGATGCTATGTGGCATCTTGAACCATGCAAAACAAAGAAAACATCA
CTCTGTCAAAAATTGCACTGTGGTACCCAAAGCTGTAGCGGACGAGGCAGATGCATGG
AGGAGCTGAACCTCTACCAGTGCAAGTGCTTTCCAGGGTTCAGTGGTCCTAACTGTGAG
ACTGTGACACACTGCCCAAAGCTTGCTCCTCCAGATGATGCAGAAGTGAGGTTTGTAGT
GGGAAGCCATGGACCTCTGGCTTTTTCTGCGGTGTGGAATTTTAGCTGTATAGAAGGATT
TATGCTGAATGGAACCTCACAGACTACTTGTCTACCTTCTGGATTGTGGAGTCACTACTC
ATTTCAGTGTGTGGCTAAGACAGATTATCCTTCCAACCTTACGGAAGCTTGTGTTGCTGC
TGCCACTGCTGGGAGTTCCATTTTGATATCTATCACTGTGTTCTTCATTTGGAAGCATTAC
TCTGCAGGAGGTGCGTGTAAAAACTTTAGAAACACTGAGGCTGACTACCAGTGTCTGCT
GCTAGAAGAAGTAGAGAGAGATTTGGAAAATACAGTCTTGTGAGCACTGTAGAATTAA
ATGCATGTTAAACAATGCTGAACTTGTCACTACCTTGTCAGACTTAAAAATGAATCATT
ACACATTCCTGTCTCTTGAAAAGTAATGTATTTATCCCATATTCATTAATTTCTGTTCTAC
CCTGACAGAGCAAGTGAGGCACACTTGGTGTAGTTGCAGGCAGCACTAATAGTTATGA
TTTGCCTTTACCAGAAAACAGATTCCAAGCATATATATGGCATGCTGTTGGGCATAATG
AATCCTCAAACAAGCATCCACGATTCCATATTTTGCATAGTTAGATACCCGTGAACAAA
AATGCAATGTACTTTGTCATTAGTTTAAGATTGCTGTAAGTGGCATGTAGACACACTGCT
CAAGAAGTAGTAATCCCAGTAAATGCATGTACTTGCTGCAGAGAGAATGAAACTACTT
TTGAAGATCTGTAATACATTACATTTCTCTACAGGCTTTCCCCTGATGTGCCCCTGAATT
CAATATCTGTATCTGTTCTAAACATGTCAGTGTTTGTACTGATTATTGCAATTGTTATTCA
CTGTGCTTTTTATTTTGAGAGTGAGTTAAACTGCTAGAATTGCTTGGAATATCAGTTGTA
TATCCATGCACTGCTTATAGTTTCTTGTAGATTTAAATATGTGAAAAGTTTAATGGGAGT
TAAGAATCACCTGCCTGGACTGTCTGACCGTTATGTTCATGTTTGTCCATAATCACTCTG
ATACTTTATACAATAGTGTAGCTATTATTTTGTAGTATACTAAAACAGTGCAACATCAGC
AGCACTTTTGAATGCTTAACAAAGTTGTTTTGTAACCCTAATAAATCAGATGGTGTGATG
GACTGTCTTTGTATCAGGGGTAAAAGCAGTGCTGATAGCAAATGTTTATGTCAATCATC
ATATATGCCTGCTTTGTGGCACAAAATAAAAGTTTAAGAAGTATATTTTCTTCTGAAT

>CD209_TRINITY_DN7436_c0_g4_i1
AAGTTGCTCATTAACACTTCCACATGAGAATTGAAAAAGAATATTTAAGACATTTGAAC
AACTTCAGACAAACTGAAAAACAACTGAAATGACAGCAAGACTACAGAAAGCAGCAA
AAGAAGAAATGATGAAGCGTGGAAAAGGATATTCAGTGCTGTTGATTATTTCCACTATC
ATCCTTGTCGCTCTGCTGGTCATAAATGTAATCTTATTGCTTTTAACTATACGGATGTCAG

AAGAGTTAAGTAAGTGCATGAATAGCACACATCCAACTGAGCACCCAAAAACTACAGC
AATGACAACAACAACAACAACAACAACAACAACAACAGCAGCAACAACTGCACCTTT
TACTAGCTGTAACCCAGATGAAATACGTTTTAACAACAGCTGTTATTTCTTCTCCTCCAA
CCTACATAATACCTGGAGTGAAAGCCAGGAATATTGTAAAAAGAAGAATTCTGATTTA
GTTGTAATCAAAAGTGCTGAAAAACAGCAATTCATGAAACTGAACACTGCAAAGGATG
GTGGATTCTATTGGCTTGGACTACAATATGCTGGTGCACAAACGTGGAAATGGGTGGAT
GGGACTATTTGCGATGTCCATGACAAGAAAACAACATATTTTGTTGATGGAAAACCTAG
CCCCAGTGAACCAGATAAAGACTGTGTCTACATGACATATCCCTCAGGACTATGGAAT
ACTGAGAATTGTGGTTTCAATTACCGATGGGCATGTGAGAGAAAGATAACTCACTGATG
AACAACATTATCTAGGAAGTCAATAGCATTATCTGCAACTAATCATGATGGCAAGGGTT
TTGAAGTTCAGAAGAAAATGCTGCCAGACAAAAATGTGAAGCTTGGTGATTGTGAATA
AGAGCTCTGTGATCGAATGCTATAACATTTAATTGCCCATTGATTTTAACATGGCAATTC
CTGATAATCATATGGAAGCATGAATTATTCTAATGATGTCTGGTTCTGTTTTCTCACTCA
GATATTTGAAATATAAAACAGCCTTGTTTGATTTCTTCTCAATGCATTTTAAGAATAAAC
TAAGCTTTCATGACTCTAAATATAATGTTTTCATGCTCTCTTGTTTTTCTCTTTGTATAATA
GGAACCAACAGACCAGAGCCTCCCATGTGGATGCAGTGGGCGGGATGGTTGGGCAGCA
CATGTGGACATTGATCACACACTAGGAATGGAGGTGCTGCAGTGGCCACAAATCTTCTT
AG

>CD302_TRINITY_DN8442_c0_g1_i6 len=1547 path=[0:0-1027 2:1028-1028 4:1029-1546]
ATATGGGACGTGAGTTGGCCCTCTTTTTTTTTTTTTTTTTTTTTTTTTTTTTTTTTTTGCTAT
GAATGGATTTTCTTTCATTTCTTGGATACCTTATAGACCAAGACTGAGAAGAAGTTTGCA
AAGCCTACCCACTTGCCTTAAGTCATTCAGAAGACAGTATTACATGGAACTAACAATCC
TTCCTTCATGTTGTGGAATACAGGAACATGAACACTTTCTTCTTTCAGGGTGGCCTGCAC
AGGGAGGTTCATTTCTGAACCTGCTATCAGAAGTGTTAAGGGTACCAAAAACTCTCAGA
CAACTGTACCACAAACACCTCTGAGAAATTAATCAGCTCAGAGACAACTGTAACACAA
CACCTCTAGAAACACATTTTCAGACGTCATTTACCTTACAGAGGTGATGCACAAATTAC
CTTTTTCTGATCCACTGAAAGAAGATATGTTGTGACGAACAACTTTTTTTTCTTCTTTTGG
TGCTGGTTCAGAACATGCATTTTCTTAATTATGTTGCAACAAACATCTGCAGCATATTTC
TGTGTGAGAAAATATTTAATTCTGTACTTTAATACCTTTAAGCTGCTACACAGCTAAAAC
CCATGAGTCCTGAAAATAACAGTGGAAAGTCTGACAAAAGACTCTACAGGTGAACTTT
TCACAAATGCATTCAGACTTTTGAAAGAAGGTAATAAACAGATCACTTTTTAGCACATT
CTACATCCTGATAAGTAACATTTCTATGGCTTTATATAGTTAGTACAAATACTGAAAGG
ATAAATATTTTCTTGTGGTTTTCAGATACAGACTTTTAAACTGTTATATATCCTGTGTTAC
ATGTTCAACTTCCTCAGAGTCTAATAAAATGTGTTCATCATGTGGAGTTCGGGCTGCTGG
TTGATAATCAAGTGATGACGATCCCAATGAGGTCTGTTTTCGTTCACACAGGAACCAAA
ACACTACTGATGAGATACTCACGAATGTGACAGACATGATAACCAGGACAGTAATCAA
AGCCTTCTTGTCACCTTGCTCCAGTGTCCAGATTTGATATGAATGACGGCACATATCTCG
GCAAGAGGCAAATCTTCACTGTATTGTTTAGTCCAGTTCGAATATGACACTTCTGAGTTG
TCAAACCATTTCAAACTATCCGTGTCTGTATCATAAAACATGCCAAGCCAAACTTCTTC
AGGTCCCTGCCAATTTGTCCTGTAAGTGTTTACTAAAAACACATTTTCTTCCTCACTGTTC
ACACTCACAAGGTCAGCACTGGAAGCTGAAGCTCTGCAGAGTTCCCTTGCATCTTCTAT
ACCAAGGGAATGTTTTGAAAATTCATCCAAAACAGTATAACATCTGTTCTTATACGATA
TCCATGAAATCTTAGTCCCAGATGGACATTCTAAACACATAACGGAAGGGCAATGGAA
TACAAAATAAAAACAGCCCCAACAAAACAGCAGTGCAGATGCCTTTTTCACTTTCTGC
ATGTTTATATTTCATAGTCTAAAACATTACTCGTACTCCACAACGTTTTGGTCTGCAAC

>CD22_TRINITY_DN22556_c1_g1_i5
TATATATATATATATATATATATATATATATATATACATACATATATAAAATCCAGTTTTCA
CAGGGTGAATCAAATGAACCTTATGATGTAATTCAGAGCTGCTAGGACACCTGTATTTC
ACAGCTGTAGTTATTTTTGAGGTCACCTTGTTGTGTCAGCAACTGTTTAAAAGAAATAAT

GAACAGATAATTTCCACCCACTGAGAAACATATGATAAATGATAAGATTCTCTGACTG
AAATTGCATTTACATTGAAATATGAGGAATGCAAGTCATATCTGAAACCAGATTTCATG
CCATTTTGTTGTCACAGAATCTTTGTTCTCTAAAATATGTTACATCATTAGCTTGAATTCA
AAAGTTCCTTTGCAAATATTTTGTTGTAATGACTGAGGAAGTATCAACTGAACTTGGGA
TATCAACTTCTGGTTTTCTTTTCTGTTTAAATATGTTATTGCTCATACTGGAATAAAAATC
TTCTTTTGACTGCATTATTGGATAATCCCTTTGGTATTTTCCTCTAATGATGCAGAGAAGG
AAAAAAGGAATAGCAATAACCACTGATATTCCAATTGCTATTAATGCTTCTGTTGCAGC
TAATAAAGTAGTTGTATCATGTAATTTTAAAGATAATGTCAATGATTTTGTTGACCCAAT
ATCATTGTGTGCTTCACAGCTGAAATTCCCAGTATCTTCAGAACTGATGTTTGTGAACTT
TAGCAGCTTCCCGGGCTGAACTGGAAGGTATTCATTTTCTTTATACCACGTGTAGTTTAT
AACAGGAGGATTGCTGCTTTTATAGTTGCAGGTTAGATGCATGTCTTTTCCTTCTCTTAA
ATTTATGGGATGGTGTGTTATTTTAACTTCTTTAGGTGGATACATAACATCTATCTCGAAT
TTCGGGGAATATGCCGATCCAACTGTGTTATCAGCTTTACACCTGTAAGTACCACTGTG
ACTGGTCTTAATTTCTTTAATGAATAAATACTTTTCAGAATTGTCACTCTTCTTTTTGTTA
ATGTACCAGGCATATGTTGTCACTTCTGGGTTACTCCTGTTAACTTTGCAGGATAAGTTC
ATAGAGTCACCTTCCATAATATCCATGGGAGTAATATGAGTAATTTTTACATCTTTTGGT
GCAAATAAAACATTCAGGATAACTGCAGGAGCACTTTCTATGACAGAATCAACTACAG
CAAAACATGCATACCGCCCAGCATGATTCTCTTTAATGCGGTCAAAGCGCAACTCTCTG
TCTTCACCAACTATTTCCCAAGACTGTCTACTACGTTTACGCCATTGATATTCTGTGATTT
TAGCATCACTGTTATTTGTGGAACAAATTAGTTTTACTTTGTCTCCTTCTTTAATAGTCCC
TTCTGGTTGTACGGTTATGGTTATTACATTTGTGGCACCTATGTGGAAGATCAGAGGAAG
TAGCAGAATGAATTCCATGTTAGTATGTTTCATTTTTTAAATTACAGCAGAAAAGCCTTT
ATCTTTGTAAGCAATTTCTTTCCAAACCAAATCAGCAACTCTGCATTCCAAAAGGAAAT
ACTTATTGCAACTGAAACAATACAGCACTTCCACTTTTTCCTTTGGCACTCCAGGAGCTC
TGTGTCCATGCCTTTAAATGTGCTTGAATACATTCTCATTGGGCACACACAAAAGGAAG
TCA

>ITLN2-A_TRINITY_DN388_c0_g2_i4
GGAACATCAACAACTCCGAAAGTGCTTTTCATCTCTGTAAATTCAAACGTGGTAGTACT
TCTAATCTGTAGGTACAAATTATATATGTGATTATCTGAAGGATGGCGATATCCAGTTTG
CTTATGTTCAGTGCTGTGTTGATGGTGGCAGAGACAGATTCAAACAACAAACAGTGTAA
CCTGAGAATTCAGAAGAGTGTTTTACTTGAGAGTATTGCTGGCTGCTCTGATCACACATC
AAATGATCAGGTTACAAACAACACACAACACCACACCCAGCAGAAGTATATGGCAAA
AAGCTGCAAAGAGATCAAGGAAAAGTATCAAGTCAAGCAAGATGGCCTGTACTACCTA
ACTACAGAAGATGGTGAAGTCTACCAGACATTCTGTGACATGACATCGAATGGTGGGG
GCTGGACTCTCGTGGCCAGTGTTCATGAAAACAACATACATGGGAAATGCACATATGG
GGATCGCTGGACCAGTACTCAAGGAAACAGTGAGAAATATCCAGCAGGAGATCAAAA
CTGGGTCAATCTGGCCACATTTGGTTCAGCAAGTGGTGCAACCAGTGATGACTATAAGA
ACCCTGGATACTTTGACATTGAAGCTGAGGACATTTCTGTCTGGCATGTCCCTAATAAC
ACACCTCTCAAGAAATGGAAACTGGATGCCATTCTGCAATACCACACTGAAAACAAGT
TCTTGCCCAAGTATGGAGGCAACCTACAAGGGCTCTTTAAGAAACTGCCTCTTATGTTT
AATATTGGAAGCTGCCCTAATAGTAATGGTCCAGCCATACCAATTGTCTATGATTTTGG
AAATGCTGAGAAAACAATCAACTTATATTCACCCCTCTCCAAAAATGAGTGCATTCCTG
GTTTTATTCATTTTCGTGTTTTCAACTATGAGAAGGCTGCCATGGCAATTTGTTCAGGGGT
CAAAGTAAATCTGTGTAACACAGAGCATCACTGTATCGGAGGTGGTGGTTTTTTCCCAG
AAGGTAATCCAGCACAGTGCGGAGATTTCACAGGATTTGCATGGGATGGTTATGGAAC
TGGCCAAGGTGCTAGTGTAACCAAAGAGATGCTGGAATCAGCTGTTCTCATATTTTACC
GTTAAAGGACTTTGACTTTGGTGAATAACAGTTTCACTCAGCTCTTTTAATCTGCATTGC
ATTATTCCTCACCAACATTTATTTTGTATTTTCATGAATACTCATTAGTGCTGTATACTTTT
TAATTACACAATCAAAAACCAGAAAGCTGAATTTCTGTTACTTGCACTGCTGTAACAGA
AGTTCCATCTTCCACCAATACTCCCAAAAGGAGGCACCATGGCAGTGAAGGAAATTCA

TAGACCCTTATCAGGATAGTTATAGCATGAGAACAGGCTGCAGCATAATCAACAACAG
GCAACAGGAAACAGCTGTTAGAACATTAGAAGGCCACAAAAGAAACAATTTATTTTAA
CTGTAGACCTTCCTGTCGATTGTGCATCTGATTTTTTAACAGATTCAGAATTCATCGGTG
CTGCAGTAAGGTAGTAGAGTAAAATCTAATAGCATAGGTGGGATTAGCTGCATTCATTA
ATTTAAATGTTAGTTAAGTATGACTCAACAAATATTGTGTGTTACACAACTGTTTCTAAC
TTCCCACACTGTTAGATCAGGTTGTGGCTTGCACTGCTGTGTTATCTTGATGAAAATTAG
AATTTTAACAAAGAGCTTTAATACCAGAACTTCTATTCACCTCTTTTTTACTAAATTTAT
TTTTCAGATCTGCTTACCTGGCTTGCTTCAGTTGTTTGAAGATTCATTTTGCCCTAAAAAT
GGTAGCATTAAACAATTCTGTTTCAGCAATAAGCTCTGTTATGTGCTTGCTTGTTTGTAA
AACTACTGTGACTTGAAAGTGCAGAGTATGGCTGTTAAAAACACTGACATCAAATTATC
TCAATTTTTCCCATTCTAAGTTCTTTTATGCTACAAGCAAATTCTTTGACATTTGTTATTTA
ACCATGTTTCTTCTATAGGATATGATAAAAGCATATTACAATTATGGAATGTATATGAAT
TCATGATTAGGACAAAATATTATGCAGATTATAAAACTGAACATACAGAAATATAACTT
TAATGGCATTATGTATTGAAATAGCAGCATGTTAGTGAAATAAAAATGAATGAGTTTAA
CAGTGGGTAGAGCAATAAACTA

>ITLN2-B_TRINITY_DN388_c0_g2_i3
GGAACATCAACAACTCCGAAAGTGCTTTTCATCTCTGTAAATTCAAACGTGGTAGTACT
TCTAATCTGTAGGTACAAATTATATATGTGATTATCTGAAGGATGGCGATATCCAGTTTG
CTTATGTTCAGTGCTGTGTTGATGGTGGCAGAGACAGATTCAAACAACAAACAGTGTAA
CCTGAGAATTCAGAAGAGTGTTTTACTTGAGAGTATTGCTGGCTGCTCTGATCACACATC
AAATGATCAGGTTACAAACAACACACAACACCACACCCAGCAGAAGTATATGGCAAA
AAGCTGCAAAGAGATCAAGGAAAAGTATCAAGTCAAGCAAGATGGCCTGTACTACCTA
ACTACAGAAGATGGTGAAGTCTACCAGACATTCTGTGACATGACATCGAATGGTGGGG
GCTGGACTCTCGTGGCCAGTGTTCATGAAAACAACATACATGGGAAATGCACATATGG
GGATCGCTGGACCAGTACTCAAGGAAACAGTGAGAAATATCCAGCAGGAGATCAAAA
CTGGGTCAATCTGGCCACATTTGGTTCAGCAAGTGGTGCAACCAGTGATGACTATAAGA
ACCCTGGATACTTTGACATTGAAGCTGAGGACATTTCTGTCTGGCATGTCCCTAATAAC
ACACCTCTCAAGAAATGGAAACTGGATGCCATTCTGCAATACCACACTGAAAACAAGT
TCTTGCCCAAGTATGGAGGCAACCTACAAGGGCTCTTTAAGAAATTACCTCTTGTGTGG
AATACTGGAAGCTGCCCTGATAATAATGGCCCAGCCATACCGATTGTCTATGATTTTGG
AAATGTCGAGAAAACATCCTTCTTATATGCACCTAATAGCAGAAATGAATTCACTCCTG
GTTTTATCCAGTTTCGTGTTTTTAACTACGAGAAGGCTGCTTATGCAATTTGTTCAGGGGT
CAAAGTAAATCTGTGTAACACAGAGCATTACTGTATAGGAGGTGGTGGCTTCTTCCCAC
AGGGAGATCCAGTAACATGTGGAGATTTCAGTGGATTTGCATGGGATGGTTATGGAAC
AGGATCAGAGTGGAGTGTGACCAAAGAGATGTTGGAATCAGCTGTTCTGGTGTTTTACC
GCTAAAAATCTTTCAGTGGATAACAGAGACTGGGCACTTCTACTCAGATTTATAAGCAC
ATATCACATCTTTCTATCTCACATTCATATAATTTTTCATTGATACCTAGCAGTGCTGCAA
CTTTTTCATTCGATTACATTGCAAAAGCCAAACAGTTTAGCTTGTGTTATTTACAGCGCT
ATAGCAAAAAAACTAACTTCTTTCCAGGTCAGTGTAGACTTTTTTGGGGATTTAGTCAGT
TAAGTTATACTATGTTATAGAATGTGAAGATGTTGGAATGTATCCATTAACCTGTTGTTA
TGCTAAGATATGGCTTTGCCTGAAACAATTTCCTTCAGTTGAAATTATGAGACTTTTTTT
TCTACAGCATCTTTACAGGTTCAGAGAATTTGTTGCTATTCTTTATATAATTTAAGAGATT
AAAACTATGTGTGAACAACAGCAATAAGTACAGCAGCTAGCTTTGTCAGGGAAGGGTC
TGATTGTCCATAAGTAAGAACTGGAAGTTAACATATGTATTATGTATAGTTCACAATCA
TCTATTCCCAAATGGTACGTCTAGTTAAAAATCTTTGACACCTATAAATAGTTGTGATTA
TTAAAAAAAAATAAAACGTATGATTATTAAAAAAATCTGTGACACTGTTGTGTTGTCAT
GGTGAAAATGACCAGTCCATCTATTTAGCACTGACCAAATAAATTGTTATTTT

>ITLN2-C_TRINITY_DN388_c0_g3_i1

TAGTGTTGCAAGCAATACACATTGAACTGTCCGGATTTATTGTACAATTGAAAAATAGT
AAAGCAGTTGTGGGTACCAGTGAAAGCACAAGGTAACACTTGGCAAATGATTATTTATT
GAAAATTAGTAAAATTGTCATTAACGGAAGCCAAAGTTCCTTAACGGTACATTATCAGA
ACAGCTGATTCCAGCATATCTTTGGTTACACTCCAGTCTCGGCCAGTTCCATAACCATTC
CAGGCAAAACCAGCGAAATCTCCACACTGTTTGGATCACCTTCTGGGAAAAAACCAC
CACCTCCAATACAGTGATGTTCTGTATTGCATTTTGTTACTTTGATTCCTGAACATATGGC
CAAGGCAGCCTTCTCATTGTTCAAAACACGGAATTGGATATAACCAGCAGAAAATTCA
TTTCTGCCATTAGGTGAATATAGATTAGCAGTGGTTTCTGCATTTCCAGTATCATAGACA
ATCGGTATTGCAGGGCCATTATTTTCAGGACAAGTCCCAATATTCCAGACAAGAGGAA
GTTTCTTGAAGAGCCCTTGCAGATTATTTCCATATTTGGGAAGAACTTGCTTTCAGTAT
GATACTGGAGAATAGCATCGGATTTCCACTTGCTGAGAGGTGTGTTGTTGGGTACATGC
AAGACGGATATATCCTCAGCAAGGATGTCAAAGAATCCAGGGTTCTTATAATCATCACT
GGTTGCACCACTTGCTGTGCCAAATGTGGCCAGGTTGACCCAGTTTCCATCACCTGCTG
GATATCCTTGCTGATTTCCTTGTGTACTTGTCCAGCGATCTCCACTGGTGCATTTCCCATC
TATATTGTTTTCATGAACACTAGCCACAAGAGTCCAGCCCCCTCCATTGGTAGTCATGTC
ACAGAACGTTTGGTAGACTTCACCATTTCTGTAGTTAGATAGTACAAACCATCTTTCTT
GACCTTGTACTTTTCCAAGATTTCTTTGCAGCTTCTTGCTACATATTTCTGCTGGCCTTGC
TGTACATTGCCATTTGTAAATGGATCATCTGAGCCATCATCATCACAGCAACCCATAAT
GCTCTCAAGCAAGTCACGTTTCTGAATTTTCAGGTTACAATGCGGATTTGAATCAGCCTC
TGCCAGCATCAGCACAGCTGTGAACACAAGAATACCTGGTATCTTCATACCTCAGGTGA
TCTGGATGAAATAACGCAGGTGAAATTCAAGAAAAACGCTTCAGTGCTATCTCGG

>ITLN2-D_TRINITY_DN388_c0_g1_i4
ATTGAAATGTTTTGAGGTTTACAGGGTGGGCTAGTGTTTTTAAAAGGCAGATGCAGATT
CCTGTGAGAAAAGCAGTATCAACAGCAGCATTAAAGTGTTTACTTTATGTATTCTCTTTA
ACAGGTCTTCTAAAGGATGACGATATCAGGACTCTTCTTCACTACTCTACTGATGCTGGC
ACAAGCAGGTCAAAATGATATGGAATGCAATCTGAAACTCCCCAAAATGTTCCTGATG
GCGAGTTTAGCGAACTGCAATGGTAATTTTACTAGTGAACCATCCTCAAACAACTTCTA
CTCATGGGATGGGAAGAGAAAATACACTGGCCACAGCTGCAAAGAGATCAAGGAAAA
GTACAACGTCAGACAAGATGGCCTGTACTATCTTACTACTGAAGATGGCGAAGTATATC
AGACATACTGTGACATGACGACCAATGGAGGGGGCTGGACTCTTGTGCTCAGTGTTCAT
GAAAACAACATATATGGGAAGTGCACATATGGTGATCGCTGGACAAGCACACAAGGA
AACAAGCAAAATTATCCAGCAGGAGATGGAAACTGGGTGAATCTGGCCACCTTTGGCA
CTGCAAGTGCTGCAACCAGTGATGACTATAAGAATCCTGGATACTATGATATTGAAGCT
GATGATGTTTCTGTCTGGCATGTTCCAAACAACACACCTCTCAAGAAGTGGAGAAACGA
TGCCATTTTGCAGTATCACACAGAAAGCAAGTTTCTTCCCCGATATGGAGGAAATCTGT
ATGGGCTCTTCAAGAAAATGCCACTTGTCTGGAATACTGGAATATGCCTAACTGACAAT
GGTCCAGCCATACCTATCATCTATGATTTTGGAGATGCAGAGAAAACTGCTAACCTGTA
TTCACCAAATGGTCGGAGGGAATTTAATCCTGGTTTCATTCAGTTCCGTGTTTTCAACAA
AGAGAAGGCTGCTATGGCAGTGTGCTCTGGAGTCAAAGTGACAGGGTGTAACCCAGAG
GTGCATTGCATTGGAGGTGGTGGCTTCTTCCCTGAAGGAGATCCAAGACAATGTGGAGA
CTTCCCTGCCTTTGAGTGGGATGGGTATGGAACAGGAGTAGGCTGGAGTGTAACTAAGC
AGATGTTGGAATCTACTATTATGTTGTTTTACCGCTAGAAGTCCTCAGCTCACAATATTG
CATGCATCTGTTTTCATGACTACTGTTTTACATCAATTCATACCTCTAGTAACTCAAGTTG
AATCAGTGTTACTTGCACTGTATTTCTAAATCAATGCATTGAAATGCAGGTAGTAATGTG
AATGGAAACAGAAGCTAACATTTATCAGTCCAATTTTTGTTACTATATGTATAAAAATA
ACATAACTATATGTCTTTTAAGAAGTTCATGCCTGCTCCTAGCCATTTTTTATGCTACCG
AGTTTGTGATATCAGTCTTTTATCCTTATGAGCAATATCCTAACAAACACATTTTGCATT
AAGAAAGACAGGAACTATTCATCAGTCTTTTAAAGAGTTTGTGAAGCTTTTGCCTTTGTA
CAATCAAACTTCATAATTAAAGA

>ITLN2-E_TRINITY_DN857_c0_g2_i1
TATCATCCTTGAAAGGGATTTCCTTCTGTACAACTTCAAACAGGCAGCACTCAATTTAC
AGATCATCCGAAACAATGAAGATACCCAGGTGCCTTGTGTTCATTGCAGTGCTGATGCT
GACAGAGTCAACCTCAAATAACAAAGAGTGCAACCTGAAAATTCAGAAAAGTGTCCTA
CTTGAGAGTATCAATGGCTGCTCTGACGGTCCAACAAATAATCGGGTTACAAATGTCAC
CCAACAACAAATCCAGCTGAAATATATGGCAAGAAGCTGCAAGGAGATCAAAGAAAA
GTATCAAATACAGCAAGATGGTCTGTACTACCTAACTACAGAAGATGGTGAGGTCTAC
CAGACATTCTGTGACATGACAACCAATGGTGGTGGTTGGACACTCGTGGCCAGTGTTCA
TGAAAACAACATAC

>ITLN2-F_TRINITY_DN857_c0_g3_i6
TATTGCTCTACCCACTGTTAAACTCATTCATTTTTATTTCACTAACATGCTGCTATTTCAA
TACATAATGCCATTAAAGTTATATTTCTGTATGTTCAGTTTTATAATCTGCATAATATTTT
GTCCTAATCATGAATTCATATACATTCCATAATTGTAATATGCTTTTATCATATCCTATAG
AAGAAACATGGTTAAATAACAAATGTCAAAGAATTTGCTTGTAGCATAAAAGAACTTA
GAATGGGAAAAATTGAGATAATTTGATGTCAGTGTTTTTAACAGCCATACTCTGCACTTT
CAAGTCACAGTAGTTTTACAAACAAGCAAGCACATAACAGAGCTTATTGCTGAAACAG
AATTGTTTAATGCTACCATTTTTAGGGCAAAATGAATCTTCAAACAACTGAAGCAAGCC
AGGTAAGCAGATCTGAAAAATAAATTTAGTAAAAAAAGAGGTGAATAGAAGTTCTGGT
ATTAAAGCTCTTTGTTAAAATTCTAATTTTCATCAAGATAACACAGCAGTGCAAGCCAC
AACCTGATCTAACAGTGTGGGAAGTTAGAAACAGTTGTGTAACACACAATATTTGTTGA
GTCATACTTAACTAACATTTAAATTAATGAATGCAGCTAATCCCACCTATGCTATTAGAT
TTTACTCTACTACCTTACTGCAGCACCGATGAATTCTGAATCTGTTAAAAAATCAGATGC
ACAATCGACAGGAAGGTCTACAGTTAAAATAAATTGTTTCTTTTGTGGCCTTCTAATGTT
CTAACAGCTGTTTCCTGTTGCCTGTTGTTGATTATGCTGCAGCCTGTTCTCATGCTATAAC
TATCCTGATAAGGGTCTATGAATTTCCTTCACTGCCATGGTGCCTCCTTTTGGGAGTATT
GGTGGAAGATGGAACTTCTGTTACAGCAGTGCAAGTAACAGAAATTCAGCTTTCTGGTT
TTTGATTGTGTAATTAAAAAGTATACAGCACTAATGAGTATTCATGAAAATACAAAATA
AATGTTGGTGAGGAATAATGCAATGCAGATTAAAAGAGCTGAGTGAAACTGTTATTCA
CCAAAGTCAAAGTCCTTTAACGGTAAAATATGAGAACAGCTGATTCCAGCATCTCTTTG
GTTACACTAGCACCTTGGCCAGTTCCATAACCATCCCATGCAAATCCTGTGAAATCTCC
GCACTGTGCTGGATTACCTTCTGGGAAAAAACCACCACCTCCGATACAGTGTTTTGGAG
AGGGGTGAATATAAGTTGATTGTTTTCTCAGCATTTCCAAAATCATAGACAATTGGTAT
GGCTGGACCATTACTATTAGGGCAGCTTCCAATATTAAACATAAGAGGCAGTTTCTTAA
AGAGCCCTTGTAGGTTGCCTCCATATCTGGTGAAGAACTTGTTTTCAGTGTGGTATTGCA
GAATAGCATCCAGTTTCCATTTCTTGAGAGGTGTGTTATTAGGGATGTGCCATACAGAA
ATGTCGTCAGCTTCAATGTCAAAGTATCCAGGGTTCTTATAGTCATCACTGGTTGCACCA
CTTGCTGAACCAAATGTGGCCAGATTGACCCAGTTTTGATCTCCTGCTGGATATTTCTCA
CTGTTTCCTTGAGTACTGGTCCAGCGATCCCCATATGTGCATTTCCCATGTATGTTGTTTT
CATGAACACTGGCCACGAGAGTCCAGCCCCCACCATTCGATGTCATGTCACAGAATGT
CTGGTAGACTTCACCATCTTCTGTAGTTAGGTAGTACAGGCCATCTTGCTTGACTTGATA
CTTTTCCTTGATCTCTTTGCAGCTTTTTGCCATATACTTCTGCTGGGTGTGGTGTTGTGTGT
TGTTTGTAACCTGATC

>LGALS1_TRINITY_DN18437_c0_g1_i1
ATTTCAGACATGGCAGAGATAAAGCATGCTACCTTAACTGACCTGGATCTGCGTCCAGG
CACAGAGATTGAAGTTGAAGGATTTATAAAGCCTGACGCTCAAAGCTTTTCTGTAGAAC
TGGGAAAAAATGATGACACTGTTCTCCTGAAATTCAATCCTTGTTTTGATTACAAGGGT
GTTAAAAAGACAACTGTGTGCAGCGCTATAATTAATGGAAATTCTGTCGCAGAACAGA
ACATCCAAAACTTTCCCTTTCAGCAAGGAATGTCAACCAAGATGCTGTTCAAATTCAAT

GGTGCCGAAGGCGTCACTATAACATTTCCAAATAAAGATAAAATAACCTTCGCTAACC
ACACAACCATTTGGACAATAACTTATATTACCATCCATGGTAACATCGATGTGAAATGC
ATCAGGATTCAGGACCAGTGATGTACAATATGACATCTGCTGTATACTACATGCTACAC
CACATTTGTCAGCTTTGTTTCTTCATAATTATGCTGATTTTTTAATATACAGATTTGAAT
AAAGCATTTATTAACACTTGTTCTGACGGACAGATTTGTTTATAAAATGAATCTTCATGC
ACATCTCTGATCACAATACTGATTCTGTTTTATTACTAAATTTAGGGTCCAGATTATAGTT
GTAGACTGAAAGGTAAAATTACTCTGTTATCTCAGATCACTGAAAATGATGTGCTGTCC
CTTGCTTATTTTAAATATTTATTTTATCCTTCAGCCATAAGCAGTCATAATTTTTTCCTGGT
GTTAGTTTATACTAGTTTGGGTTATTCTGTGACTATGTGCGCACAATAGAGTAACTATGT
AGCTACTTGCTCCACATGTTTAACTTACGCTATTTCTTCCAAGTGTTCTGAACATAATCGT
TGCTTGTAACATGATGTCTTGTTTACTTGTTTGTTATAATCTGTAACCCAGGCTGTAACAT
GGCTCTAGAGGTGGGTAAAGTTAGAAATGAGCAGAGGTGGATGAGGAGATGGAGACC
TATAGATTGGAGGGCAGAAAAGGGAGGAAGAAACCAGAAGGAAGGGGCTGCAGAAA
TATGGAGGAAACTGAGAATGCCACTTAAGTACTGGGAACAGATGGTAGTACATGAGGA
GAAAAAAGAATGGGGGCAAATAAGAGAAGAGTATGGGCTTGAAAATAGACATATACT
GAGATACCGAGGTTTAAAAAGAGAAGAGAGTAAGACAAGAAAAGAAGAGGGACAAA
TAAAAGCAGAAGAATGCCCAGAGTAGATAAAGGTTCTTATAGAACCCAGAGGAACAG
TGACAAAAATAATACTAAATTGATATCGAGGGCATACCAGGGCATAATAATAGGGTAT
AAGAAAGTGAGTGAACACATGAGACAGACATGGCAAATGAGATGGAATAGGGAGATA
TCAATAAAACAATGGGAGGAAATATGGAAAGCCCCAAGATATGCTACAAAGTCCATG
AAATTTAGAACTTTTGCATGGAAATGCTTTCACAAGTATTTCTATACATTAGTGAGGCAT
AAGAGGTTGTTTCCACAGACAGATGAAAGGTGGTGGAGATGTGAGACAGGACAAGTTG
GAACATGGAAGGATATCTTTTGGGAATGTGAAAAGCTGAAGGGAATTGAGGGGGAAGT
CAAATAAATATGGGCAAGGGCAAGTCTAAACAATAAAACATTTACAGAAATGGGAAT
GGACATAGAGATAGACCTGACCTGGAAGGCAAATAGAGAGGTAAGAGAACTCCTGAC
ACTTATAATAATGGTATACAAAAAAGCAATTACAAAGAAATGGAAAAATAGGGAGGT
CCCAGAGGTCACGGATCTTCATGATATCATGTGGGATATGCAAAAGATAGACTGCTGG
GGCTCCCAAGTCCCTAAAAGGGTTAAGAAAAAACGTGGTATTTTGTCAGCATGGAGAA
AAAGAAGATGAAAAATGACAGTTCCTTTAGTTATTCCTCACAGAAACCACTATGGGAT
GAATGAATGAATGAATAAATGAGTAAGAGTGTACAATAGAGGGGCGAGACATACGCG
CTCAGCCTTGACATGTAAATAGTTGGGTAGCAAGGTTTGAAATTAGCT

>LGALS9_TRINITY_DN2268_c0_g4_i4
AATATGAATGGTACATGCTTTATTGTTTCATTTGGTCCATGAAAATGTAATTGGTGAAGT
GAAGCTTTAGGAGGTAACTGGACAAATGATGATTACTGCTAAAAAAAAAAATACATAT
CTGAAAATTTCAGACATGGTATGACTGGACAGGCATTTACTGTGTTTTCAACAAAAACA
GGTAGATACAGTAAGGCTTTCATTATATAAATGATACAAAACAGATTATACAAACACT
GAAACATGAGAGTGTGACTAAACCTGAAATATTCTGAACAGATTTACATTTTTGTATCT
CTGCTTAGACAACTAAGTTTGAACCTCCAAGTTAACTATTATCAACAATTTATTTATTTA
TAACTTTTGTTTACTGGGAAGACGCCACTGGAATACAAGCCCATTTTCAGTGGGTGCCC
AGGGAGAAAAGCCCCACATATACAACATTAAACAAAGACATAAAAAACAAAACATTT
TAAGTTCCATGGGTACACAATAAGCTTCAACAACTGTTAACACACATCAGTAAATACTA
AAAGTTTTCACATTTTTTTTAGAATAAAATTTATAGAAGTATTTAAGCAATTTATACAAA
TGACAACGTGCCTACATTTTCTCTCACACTAACATCTGATACATTGTTTAGTAGTTAGTC
CATAAATGGTAATGGTTACTAAACTAAGACAGAACTATAGAAGCTACAAATCATGCTA
TATGTTTCTAGAACTTATAAAAATATTCTGAATAATGATTTTTCCTTTCAAAAATACACA
CTTGTAAGCTGGACATTCCCACTTATTTCAAGCTTCTTTATTGACTGAAGGTCAGATACT
CGATGGTTGAAGTTAAACATGTTTCCATTTACACTGACTCTGAAACAATGATGTTCACA
GATAATGGAGATCTGAAAACTCTGCCCTTGGAATATGGGCATTGTGCCATTAGGCAAAC
TCCGTTCCTCTGCTCCCCAAGCATTATTCAGAAAACTATTTCGAACTAAAGCATTTTCTC
CAAATCTTACAGCCAGATGAAATGCAATGTCAGAATTCCTCTTCAGGTCAATACGGAA

GCTGTCAGCATGATGAGGCACTGATCCAGAAATGGCAATGTTTCTTGGTGGATACAGCC
CCTCTGGTATGTAAACTTCATATGGAACTTGAAAATTAGCTGACTGAGCTGCAGCAGAC
GGGTAACCGGGAGCTACTGGTGGTGCCGCACAGTAAGTGGGGAAAGCAGAAGCAGTT
GGTGGTGCCGCAGAGTAAGCGGGGAAAGCAGAAGCCGCCGAAACAAAAGCACCTGAA
GATGGAAGCACTTGTGAGGCCCGCATGTCCTGAAAACTGATGACACTCAGCTGAATCTC
TCCTGACACACCGATTGTATCCACCCTGGACAGAGGGATCCTGTGTTTATATTCTAGGA
AATGTACCCCATTCACTGCCACCATGAAGGAAGTAGTTTGCGCAAGAAAAAGAATTTC
AAAGCGCTGCCCAAATTTAAAGGGCATTTCATATTTCCGCTCTTCTGAACCCCATGTCTC
ATACTGTAAAGTATTGCACACGACACAGCTCTCGCCAAAGCGTGGGTTGAAGTGGAAT
GCAATATCGGAACGAGGAGTCACATTGGTTCCACACTGGAAATTCACAGCAAACCTAT
CACAGTAGCTGTTGACCTGTCCTTGTACTGCCACTATTTTTCCATCATAAAGACCACCAT
GAATGGCACCAGTAAAGGGAATTGGGGGACTGTAGAAAGGCGCCTGAGTCTGAAACC
CCGACATTGTTCGTACAGCCA

>CLEC2L-A_TRINITY_DN98733_c0_g1_i2
GGATTTAAGTTTTATTTTATTTTTCTGCAGTGTCATTTATAACCACATTTGAAAACATCCT
TAAAAAGCAAACAGATATTGAAAGCTTTATAGTCTTAGTCTTTATTTTTATGTCATGCTG
TCCAGTAGAGCAAACTGTTTTGTTAAAAAAAAAGGTTGCTTCATTAGGATAGCTGTACT
TTACCATTTTTTTTCAATCATTTTACAAATATGATATTGTGACTCCCTTGCAGAGCTGATA
AGACAGCAAAAAGCTATACCTAAAAGTAATCAGAGGTACATTCTTTGGAGAAAGAGCA
CTCTACTGATATATCATCATTTAGTTCTTTGGATCAGTACTGTCCAGTGTTGCTCTGGCAG
ACAAGCCTTCATCCCTTCACATATACATATTTGTAGCAATATCATCATAAACGTATATCG
CTTGCTCTTATTGCAAGTGGTTAACTGCTTTCTTCACACAAATATAAGGAAAGAGTGCTG
AGCAATGACGATCAAATAGCATATCAGGTTCAATAGTGATGCATTTGTGCTCAGGACTA
ACACTTCCTCTATTGATTCTAGTATCATTACCATCAATACTGAAGAACGTATCAATCCAG
TGAGCTTTATCTTTTACAAATTTTGCTGCAAATTCCAACATGTCATTGTTTAGATGTGTAA
ACAATGATGCATTCTGAGAAGAACATGACTCTTCTGAGGCATTGTAGTTTTTTTCTTCTTT
GGAAAACAGGTAGCATTTGCTTTTGTATCCTATCCAGTGTTCTGGGCACATATAGTAATT
CTCTGTACCGTTACCATGTGCCTCTCTGAATAGTCCTCTCTGATGTTCCATCTGTGCATTA
TTTAACTTTTGAGTATATTTATGAATAAGCAGCACTTTCCACGCAAATATCAAGGCAAG
ACAGGTGATGCTGAGTGCAGTCATGGAGTAATAACAGAACAGCAGCTTGCCCATTCTCT
GAGCTGTAGAACTCTGTTGTCCAATTCCACCTTTAATTTTTTCACACTTGTATCTTCGTAG
CTTTGTCAGTGT

>CLEC2L-B_TRINITY_DN25632_c1_g1_i7
AGCAATATGATGGCCTTGAATAGACCTTGCAAAAATTCCATCCATTCTTTTTCTTGAGAA
GTCTGAGAAGAGGATGTACTTTGTCTTCTGTTTCCTCTTTAGGTGTTTTGTCGAGTTTCAT
GAAGTGAGGCAGATCAGACCCCTGGAGTACCTATATCAAGGCACTAGGCACATATTCC
CTGATTATGACTTCAGGTGTGATTTTGGTCCCTCTGAAATAATCAGCCTTGGTTCCTCCTT
CTTAATCATATCAGTATGATCAGTCATAACGGATGTGTGAGAATCAGATGCCATACTTT
CTGCCAGCAGGATCACTGTACCAGTAGCCTGAGAAGAAGGTGGTTGTACCATAGGTTC
CTGAAACATCTTTAAAAGGTACATCTGAGTCTGACTGGGCAGAAGACGTTTCAGGCCTA
GGCCTTTTGAAATGAGTCAGCAGGCTGTCTGATAGTGCATATTGTGCCATTTTGTACTGA
GCGAGGTGTTTCAAGCTCTGAATCTCTAACATGTACTAAGAGAGGACAAATGTGTATGC
AGGGTTTTCGGAACAAATGATTCACAAATCTGACACTTCTGATAATTGTGTTCAGCCTC
ATGACAACACAGGCACTGGGAGTGGAGGTCCTTATAGGGAATATGGCCTACCACAATG
ACAATTCCCTCTCTTACCTGACCAAATAACAATTCTGAACACACAAGAAAAATGAGGG
TTCCCAATGGAGCATTTATTTGTATAAGTGAAGGCCGAGGTAACACTGAGTAATATGTG
GACCAACAGACCGACAAGTGCGAGACCTACATTTGGACTGGTGCAGAGATAATCTTAA
GGATCTCACATTTGTGTGTCTACCCATATACCCCATACGCCAGTGAGCTTGGGTCATACA
TCAAACGTAGGAGCATACATGAGGTTTTGCTTTGGTATACCATCCAGACATCAGGTTA

CCTCGAAGAGAGAACCCATACAACAGTGATCTAAAGAAGGAAATCAATAGAGATAGA
GGAAAAACATCATCATAATGCCAGAACTCACTAGAAGCAGACCCACATGTGCTCAAAT
AATCTAAGCTAAGTCACATAATCAAGCCTGCTAAAACAACCACATGCACACACCACCA
CAAGGCTCCATTCTGTTGCTGTAGTATGGAAATAAGCAATGGGAAGCTCAAATATTTAT
ACTTAAGATATATTCATTTTTAAATAAGTAACGTTAATACTATTCAAGGTCAACAGAAT
ATCTCAACAATCTCTCTACACTTCTGCTTATCATAAAGTTTAAAACTGAACTGCTACATT
AGTAAGAAAGCTCCCCAGAAAAACAAACATCGCAAGTTACAAGCAGGTTCACACAAT
TATTACAACCATGTATGTCAATCATTTTTGGATACAGTAGATTTCTTTAAAAAAATATTC
TGTCCTAGACATTTTCTTGTACTTAAAGTGCATGACAGTCAAACTACACCATAACTATAT
ATTTTTTTGCTTAGTATTAGTTGTTTAAATACTTACTAAAACATATACACCATAACTTCAA
GAACAATATGAAACTTTTTTTTTCTTTACATATAAATTATCTGAGTGTACAGGTCATTAA
ATCAGTGAGAGTAAAAAGAAAAACACTTCAGTCAATATTTCTCTTAAAATGTCTTTCAT
GATGTGGTGTGATGGGCAGAAAACATTTCTGAAAAGACAAACAGCCTTCTAAACTGGT
CGTGCTTCCTTGCCACAGTGGTTAGCATCCCCAGCAACAAACAACGCTCTTTGGTTTAGT
ACCTCAGTGATTCCATCTCACAGATCCAAAGTTGATGGATGTTACAATCTAAAGACAAC
AATGTAGTCCCCTCTCTTACTCCACAGTTGAAATCAGGCTTATCATCTCCAAGAACAGT
GCTGCTGACATTGGAACCATCTATCCACTTCCACTGCTTGTCATCAGATCTTGTCAGTCC
AACCCAATAACTAGCATTAGAAATACTCAAAAAATCCAAGATATTGGTGCCTTCAAGT
ACAGCCAGATCAGAGTGCCGAAAATTACACCACTCTCGACTGGTGTTCCAGCTCTTGCT
GCTGTTCCAAGATGCGTAGAAGCATTTCCCATTGTGCAGAAACCAGTCTTCTGGACAAA
GTTCACAGATTCCTTTTCCACTGCTATAGGAAGTGCTACATAAGCGTAGCAAAACGTCT
TGAGCTTGAGCAGCACGAGCCAACTCTGTCTCATTCATGGTTGAACGAGCGAGACCCA
GATTCCAACAAGGGGGCGCAATTACCTGCAATATTATAATAAGGATAAAGAGTGCATT
GAAAACAGCGGATATCCAGCACAGAAACCGAAGAGACCAGTCTTCATCGTCTTTCTTTT
TAAATGGAAATGGAGGTTCCCTTGATTCCTCTCTAGGCAGAGGAGACTCTAAAGTAAAG
TGTACTGCTTCTGGGGTCTCATTCTCTGAATCTCCAGCAACTTCATCAAGATCAACCTGA
GAAACGGCAGAATAGTTTCTTCTTATGGAAGCAAAACTCATACTGATGGTGGCTTCTTA
ATTCTGAAGTGTTAATTTTCGGATGGAACACTGAGCTAGTCTTTCAGACTTAAGACCCTC
TAGATTTTTCTCTCCACAAAGCTGAAGTCCCCTGAAGAAATCTGGCAAGATGCAAGTTT
ATTCAGCTTCACACTGCAGTCTTCTTACTCAAAAGGATTTTGCTCAGATTGTTAACTCAG
AAAATGAGCCACTCAGAAACCTGTTATAATATCAAACCTCACCCTAACTCGACAGTTTC
CGGCGTGTAGGATTACTAATTCTCACATCGCACGCTCTATCTGTATACACTGG

>CLEC2B-A_TRINITY_DN2263_c0_g2_i2
AACTGTCGAACAAGTTTCGGATGATGAGAGGTTGAGAACGATATATACGTGTGGTTATT
AAGGAAATGCAGACTTTACTTGAGGAAGCATGTTTCTGCAGTGCTTGTCATGTGCAGGG
GAGAGCAGGAAATTGGAAGGAGGACTACAACTGTTGCTCTCATATACAGCATTAGAAA
TGAATTGTCTCCTTCTAGTGACAGCTGTGCTTGCAGAAGGCTCCATATAAACTACAATA
AAACTTTTGGATTTTGTAACATGATTAGAGCAAGGAGCACCGAGAACCAAAGAACACC
ACTCAGGATGTCTTCATCTGAGAAGTCTAGCAGATCACTCGCAAACTTAAGTTCAGATA
TTTCTTTCAAAACATTAGTGTTTGTTGTGCTGACTGCAATACTATCAATATTCCTTTTGTT
AAGCTATATTGCGCCTCACTGTGAGATGATTTATAGAAAAAGTGTTTCGACTACAGATA
GAGCTGCAAAGGACTTGGACATCTTAAACAAAATGAAAGAGCTTTGCCATGAATCAAA
GGCTAATGATAATGAAAATGATAGAGCTGCAAAGGACTTGGACATCTTAAACAAAATG
AAAGAGCTTTGCCATGAATCAAAGGCTAATGATAATGAAAATGATAGAGCTGCAAAGG
ACTTGGACATCTTAAACAAAATGAAAGAGCTTTGCCATGAATCAAAGGCTAATGATAA
TGAAAATGAAGTTTCTTGTAAGTTGTGTCCTCATGGATGGATTGGAGATCCAAAAAACT
GCTATTTCTTCTCAACTATAGAAAAAGATTTCAAGAGCAGTGCAGAGTGGTGTTCTTCA
AGACATGCATCCCTTGTGCTGATTAAAGATAAGGAGAAACTGAACTTCATAAAGAACT
GGGCCTACAGCAATACATACTATATTGGACTGAAGCGAAATGATATGGGACAATGGAT
GTGGGCTGACAATACATTCCTTGATAAAACGAGATTCATACTGGAAAACACTACTGAA

CTTAACTGTGTTTTTATGGAACAAGATAAAATTCATCCAACTTCTTGCAACTCATATCAT
CGATGGATATGTGAAAAACAAGTTACAAGAATATGGTGACCAAATAATGTTAAAAAGT
GCAAAAAAAAATTCTGGCTCTGTTTTTTTTTTTTTTTTTATTG

>CLEC2B-B_TRINITY_DN2263_c0_g2_i4
AACTGTCGAACAAGTTTCGGATGATGAGAGGTTGAGAACGATATATACGTGTGGTTATT
AAGGAAATGCAGACTTTACTTGAGGAAGCATGTTTCTGCAGTGCTTGTCATGTGCAGGG
GAGAGCAGGAAATTGGAAGGAGGACTACAACTGTTGCTCTCATATACAGCATTAGAAA
TGAATTGTCTCCTTCTAGTGACAGCTGTGCTTGCAGAAGGCTCCATATAAACTACAATA
AAACTTTTGGAATCGCTCCCAAAGTGGCTTTGCGGGGTATTGCAAATTTCCATGGAGGT
GTGCCTATTAAATTTTCATTGGGCTGAATTTCATTGAATATATTGGATTTGCTTTAGAAAC
CGTGGCCCGCGACGCAAATGGTTTTGTAACATGATTAGAGCAAGGAGCACCGAGAACC
AAAGAACACCACTCAGGATGTCTTCATCTGAGAAGTCTAGCAGATCACTCGCAAACTT
AAGTTCAGATATTTCTTTCAAAACATTAGTGTTTGTTGTGCTGACTGCAATACTATCAAT
ATTCCTTTTGTTAAGCTATATTGCGCCTCACTGTGAGATGATTTATAGAAAAAGTGTTTC
GACTACAGATAGAGCTGCAAAGGACTTGGACATCTTAAACAAAATGAAAGAGCTTTGC
CATGAATCAAAGGCTAATGATAATGAAAATGATAGAGCTGCAAAGGACTTGGACATCT
TAAACAAAATGAAAGAGCTTTGCCATGAATCAAAGGCTAATGATAATGAAAATGATAG
AGCTGCAAAGGACTTGGACATCTTAAACAAAATGAAAGAGCTTTGCCATGAATCAAAG
GCTAATGATAATGAAAATGAAGTTTCTTGTAAGTTGTGTCCTCATGGATGGATTGGAGA
TCCAAAAAACTGCTATTTCTTCTCAACTATAGAAAAAGATTTCAAGAGCAGTGCAGAGT
GGTGTTCTTCAAGACATGCATCCCTTGTGCTGATTAAAGATAAGGAGAAACTGAACTTC
ATAAAGAACTGGGCCTACAGCAATACATACTATATTGGACTGAAGCGAAATGATATGG
GACAATGGATGTGGGCTGACAATACATTCCTTGATAAAACGAGATTCATACTGGAAAA
CACTACTGAACTTAACTGTGTTTTTATGGAACAAGATAAAATTCATCCAACTTCTTGCAA
CTCATATCATCGATGGATATGTGAAAAACAAGTTACAAGAATATGGTGACCAAATAAT
GTTAAAAAGTGCAAAAAAAAATTCTGGCTCTGTTTTTTTTTTTTTTTTTATTG

>CLEC2B-C_TRINITY_DN2263_c0_g2_i7
AACTGTCGAACAAGTTTCGGATGATGAGAGGTTGAGAACGATATATACGTGTGGTTATT
AAGGAAATGCAGACTTTACTTGAGGAAGCATGTTTCTGCAGTGCTTGTCATGTGCAGGG
GAGAGCAGGAAATTGGAAGGAGGACTACAACTGTTGCTCTCATATACAGCATTAGAAA
TGAATTGTCTCCTTCTAGTGACAGCTGTGCTTGCAGAAGGCTCCATATAAACTACAATA
AAACTTTTGGATTTTGTAACATGATTAGAGCAAGGAGCACCGAGAACCAAAGAACACC
ACTCAGGATGTCTTCATCTGAGAAGTCTAGCAGATCACTCGCAAACTTAAGACAGTAAG
CTATATTGCGCCTCACTGTGAGATGATTTATAGAAAAAGTGTTTCGACTACAGATAGAG
CTGCAAAGGACTTGGACATCTTAAACAAAATGAAAGAGCTTTGCCATGAATCAAAGGC
TAATGATAATGAAAATGATAGAGCTGCAAAGGACTTGGACATCTTAAACAAAATGAAA
GAGCTTTGCCATGAATCAAAGGCTAATGATAATGAAAATGATAGAGCTGCAAAGGACT
TGGACATCTTAAACAAAATGAAAGAGCTTTGCCATGAATCAAAGGCTAATGATAATGA
AAATGAAGTTTCTTGTAAGTTGTGTCCTCATGGATGGATTGGAGATCCAAAAAACTGCT
ATTTCTTCTCAACTATAGAAAAAGATTTCAAGAGCAGTGCAGAGTGGTGTTCTTCAAGA
CATGCATCCCTTGTGCTGATTAAAGATAAGGAGAAACTGAACTTCATAAAGAACTGGG
CCTACAGCAATACATACTATATTGGACTGAAGCGAAATGATATGGGACAATGGATGTG
GGCTGACAATACATTCCTTGATAAAACGAGATTCATACTGGAAAACACTACTGAACTTA
ACTGTGTTTTTATGGAACAAGATAAAATTCATCCAACTTCTTGCAACTCATATCATCGAT
GGATATGTGAAAAACAAGTTACAAGAATATGGTGACCAAATAATGTTAAAAAGTGCAA
AAAAAAATTCTGGCTCTGTTTTTTTTTTTTTTTTTATTG

>CLEC4E-A_TRINITY_DN90481_c0_g2_i3

CAGTAGGAAGAGACATATCTCAACACGTATCACCCAAGTAAGATTTGACAAGTGTTTC
ACTTTTTATTTCTTATCAGGGGACAGCAGACTATTTAAAAAAAAGTCATATTGCCCTCTT
CACACAAAATACCCTATTGATGCTTTCTCACAAATCCAGTTATGTTTTATTGAGCATTTA
ATGTCAAGCCATGTTCCGTTTACTTGCAAAACAACACAGTCATCCACATTTGACACTGC
ATCCTGGTTGTTAGGTCTGCCTTGTGCCCAGAACTTGTCTGTGGCGGAAAAATCAGTGCC
ATCCACCCACTGAAATTTGCCTTCTACTGTCTGGTCACTAAGTCCAATGAAGTATGTTTT
ACTTGCAAGGGAACTCTTCAAAAATCCCTGTTCTGCTCCATCATTGATGATAACAAGCT
CAGATTTCATAGATATGCATCTCTTATAAGCAGTATTCCAGTTTTCAGTCTCTGTAGAGA
AGTAGTAACATTTTTCTTTATATTGCTTCCATGGGCTCGAGCAGGGAGAACATAAAAAT
CCACTTTTATTGGCAGATGTCTTCTTGCACAATGCATTGGAGAGGATTTTGCTGTCACTC
TGCAGGTCTTTGATGCTTTTGGCTTGACCATCATTTTTATTGTGGAGTTCAGAAATTTGGG
ATGACATCTTCTCCTCCAACTGTATCATTTCATCAATCAGTGCATCATATTTGCGGTATC
CCACTGTGAAGAATGCTATAATCAGCACAAAGCAGATCATTAAAAAAATGACTAGTAT
GATAAGGAGCTTTGCTGGCACCACTCTCTTCCTCGCTTTCTTCATAGATAAAAATGCTGA
GTTGTCTGGACTTCCTCCATCTTGTTTTAGTGGGTTACTTGAAGTATCTGGCTTTGGAACA
GGAACATCTAAAACACAAATGTCATTCTTTCTTTCTTTCTCTTTCTCCAGTGGTATCCTTA
TTGGGGCGTTCATGTAAACGTGTTCCATCTCCATG

>CLEC4E-B_TRINITY_DN68583_c1_g1_i2
GTGTTCAGCAATACACTGTGATGCAGAAGGAAATGGCACAATACTTGCAAAGTTTTTAG
AACACTTGTCCCTTTTCATTAACATTTAAATACTAAAGAGCAGACATGATTTATGAAGA
GCTTAATCTTCCAACTCAGGACACTTACAATACAATCGAGCAGCTTGAGACTGAGAGA
CAAGGGAAAGAAATGACACCAGGACTGAAGAAAGCTCCGGCAGTACAAGATGATAAA
GTGAAGCACAACAATGGATGCACAAAGATGCTAATTACACTGGCTGTCACAGTGATCA
CCCTGCTGATAATAATTATAATCTTAATTATTCTGAATCTACAGATGTCAGAACAGATA
ATTCAGTACAAGAGGTCATCTGTGTTCCCAAAAATTACAATAATGACAAATGGAGGGA
GTTGTTTCTTATCATGTCCAGATGAATGGCTAGAGTTCAAACAGTCGTGCTACTTCTTTTC
AAAGGACAGCTTCAATTGGAATATGAGCAGATATGCCTGCATGGCTAAGAAAGCTGAT
CTGGTGGTGATTACCAGTAAAGAAGAACAGGACTTTATAAACCATCACTATATTGACAT
GGCCTGGATTGGTCTAACAGATAATGAGCAGGAAGACATTTGGAAATGGGTTGATGGC
ACACACTATGACAACAAAACCAGCTTTTGGTGTCGTGGAGAGCCTAATAATGCACTGGT
GCACAATACTGAAGGTGAGGATTGTGCCATGCTGTGGAATACTACATCATGTCACTTAG
GCTGGAATGATTTAACTTGCCAAAGAGACTTGAAGAGAATTTGTGAAAAACAACTCTG
CACAGTGCTTATGTAAACAGTGGCCTATGCAATGACTTTGACCAGCAGAAAATGTGAG
AAAGTATGTATGCCGGTGAAATATTTGGCAGTAGCTGCCATTTTATTTACACAACCTCAT
TAACAACATTAACATCAATAAAATTTGTTACCCACAAATAGTTAGTGTCTGGCCTGTAAT
GTTACTTTATTCAGTCTTGCACCCACTTTTAGTCTGTAATCAGTGACATGCAAAAAGTTT
TTAGACAGTATGCTATCAGTTTATTTTACTGGCATTTATCCTGGTTGTATTTCCTCTACAG
ATTCTCAGAGTTGTTATTGTACTTATGATATACAGCACTTTTGGCTTAGTTTAGTTACTGT
TTTTTTTTTTTT

>CLEC4E-C_TRINITY_DN46240_c0_g2_i1
GAATGGAAGATGAGGTTTACAGGATTTTTATAATCTCTTCCACAATCAATCATACTTTTG
CTTTAAAAGGCAAATGTACTTATTAGGTTAACAGTTCAAGAGCTAATATTGTTGAAGTT
GTGTTGCAAAATAATGATATGACAGCTTCTAAGTAACTGCAGTGAATCAAAATAACTTT
TTCAACAAATTGCCATTGTACCATAATAAAATGCATACATTTTTAAATAACAGCAGAGA
TAATGATAAACTCAAACAAAAAAGTTCAGTACATAGTTTTTCCTTCAAAACCTTGTTTCT
GTGACAACCATTTTCTTCATGCTGACATCCACAAAAGTAACTATATAGGAACTATGTGA
ACCACTAACTGAAATAATGAAATAATATGTTACTACTGAAACATTAGGAGGATGGTTCT
GTATTTGTCTTATCAGTCTGACTTTGTAAAAGTTAAACTATTTAAATAAACAGTGTTTTA
GCACATGTTGTTGTTTGCTATATAGTCAGCCAAATCAACAATGAAATGAAGCAGATTTA

TAAAGAGTTGCATTTGATCTGTTTTTCACAAATAAAATTGAAGGACCGTTGGCAGTTTTC
ATCATTCCACTCCAACTGACAAGGAGGCCTAGTCCAGAGGGCTGCACAGTCTTCACCCC
TGTGATTGTCACCCTTTGCATCATTAGGTTCACCTTTGCACCAAAATCCAGTTGAAATGT
TGTGTGGTGTTCCATCTATCCAATGCCATGTGCCTTCCACACCTTCATCAGTTAAGCCAA
TCCAGTAATTGTTTCTACACTTATTTTTAAGAAATCCTGCTCTTCCTTGTTGTTGATCACT
ACTAAATCGGCACCCATTTGCTGACAGTATTGTCTGCTGCCGCTCCAAGAGTCAGTGGA
AGTGGAGAAGAAATACAGGGACCTCTTGAATAAGTTCCAGTTTTCAAACAAGACTGAG
TCAAGCACACAAAGATGTCCAATTTCCGAGGGTATAAAGTATCCACTTTTTAATTCAGG
CAGCGCTTTTGATATTGGGCTGTAGTTACTAACCATCTCTTGCATTTTCTCTGTTTCTTGTT
TAAGAAGGTGTGACATTTTGTTATAGTTCACGCTTAGAAGAATAGGGGATGCAAGCAG
ACATGTTGTAATGAACAGCAGAACAATTATCAAAGACCATAGTATGGGTTTCTCATTTA
ATTTATTCCATAGTGTCTGCCCTGCATGAGAGTTTAATCCAAATACATTCTGTGCAGTGG
CTTTATCCATCTCAGGTTCAGAAATAGCTGCATGGTCATTTTCAGATTCAAAAAGCAAA
GAATTATATCGAGCATCATATTCCATTGCTGCAAGTTCTGCTGCTTATTTCCTCAAATAA
CTCAGTGTTAGTGA

>CLEC4E-D_TRINITY_DN23661_c0_g1_i1
AAAAATATGATTTGGATGTTATTATTTCAACAGGTTTATTTTGTATTTACACAAAGTTCTT
CTTAATTTTTATATACAGTTTGCTAGGCTACCCAAAACAGTTCTGCATGAAGAACCTAAT
TCCCCATTTTTAATTTCTGTTGCATTCACTTTAATGTTATTCTGCATTATAAAAACTCTAT
AGCAAAGTAATTCCAAAAATAGGAAAAAGAGGAAAATGTGCACAGGGAACCAATGTG
CAGTTGGAGGGGCTCGGTATAATGGTGAAATTGCTTTCTTTTCAAATACCATTGACTTAC
AACAAATAAAATTAAAACAATATTTACAGATATCATCTATTGCCACATTAGCATGAATC
ACGTAGTTTTAGGTATGCCTATGGATTAGAAATTATGTCATAAAGTCATGGTCAGCAAG
AGTTTCTTCCAGTGAGATTAAGTCTTGTTTAGTTTCTGACTGGCAAGTTTTAGCCAACTG
ATGATAGTCATGTCCACATTAGCATGTCTGCAGGTCAACAGCTTGAGCCCCACTCCTGT
CTGAAATGATTCCTTGCACTCAGCAAGGATGGTAACCAGCAGTCATAACTCAAATGGTC
ACACACTGATGATCGTCCCTTTTTTTTCACAGATGTATGAATAATTTAAATGACATGACA
AGTCATATGTGATAAGTCCAGAAATTGAAGAAATGATTATTGCAGCACAATCCTCTCCA
TTAGAATCGTGACGCTGGCTATTATCAGGCTGTCCAACATCCCATGCCACTTTTCTTTTG
CTAGTATCTTTGCCATCGACCCAGACCCATTCACCTTCTTTTTCGATATCTGACAATCCA
ATCCAATACTTTTCACTTTTATTTGCAAACCAGCTAAGAAATGCCTTCTCCAGGTCATTG
TCTATGACAACATAATCAGCACTCTTATTCTTACAGTCCTCCCTGCCTTGCTCCCAGCTT
CGTTTGCTTGCAGTTGAGAAGAAGTAGCAGCTAGTACTTAATGAATCTCTATCACTTGG
ATGGAATGCTGTCCATCCAGGTGGGCAGATCTGACATCTGGAAATTGCACCACGTTGCA
CACAAAATGAATCTTTCAATTCAGAAAGTGCCTTAGAAAGAGATTGCTTGTCAGATCTC
AGCTGGAATATTTCTACATCTTTCTCATTCGCAGGTCTAGAAATTTCACTGCAGTTGAAA
GGAATCTGTTTGACTTTTGATGCTTCATTCAACTCTGCAGAAACAAAGCTGAAATTTTCT
GAAACCTCCCTGTGTTGGGCAGTTAATTTTTCATATTGAGCCGAGAGTAATTCTGCTTTC
TCTGCCTGATGAGAATGTTGCTTTGATGCATTGTTATACATCGTCGCAATGGCTACAATC
CCAATCAGCAGGATGCCGCAGAGAAGGAGAAAGGCTGTGACAATGAATCTGGTTCGCC
AGGATATCTTTGATGTGTCCGAATCAAAACCAAAAATGCCTGTTTTACCTTCCGCTGCTG
GCATTTGTATAGGTGTGTAGATGTCGCTGTGCTTTGGTGGATTCAGTTTGCTGTAGGATT
CTTCATTAACTGGTTGCAGTCCCTCGTATATGTGCTCATTTGCCATTGTTGCTTTCTCTGG
CTCTTTCTTTCTATCTCCTTCTTCCATCGGGACTTCCATCCCCTCAGGTCTCCCAAACCCA
TCCTACCAACCTTGCCTGGGGCTGTATGAGACATGGGGATCCCGTGGTCAGGAGTCTGT
GCATCAGCCTGGTGGGTGCATGTGTGTGAGCTCCATCCCATCATGG

>CLEC4E-E_TRINITY_DN383_c0_g1_i7
CCTTATTCCCTAACTACTTAGTCGCAGATATAAAACTTAAAAGACATTAGCAAAAAGTA
GCTTCAAAGGCAGGTGACAGAACTTACAGTAAGATTTATTCATCAAATTTATTTTTAAGT

ATTAGCTAATTTTTGCATTTGATGAACATTATGGAACAGGAGAAGACATACCAGGAGCT
TCAGTTTGCAAATGAAAATATTTACTCGGAACTTGATCAACAACAAACTAAGAAACTA
GGAGCAGAAAAGACAGCTCCAGAAACACAAAAATATGTGGCTAAGTATTGCAAACAA
CCAAGAAAATTGTTAATCACCTCCATTATCACAGTGTTCATCCTGCTGGCAGTAATCAT
AGCTGTGATTATTTGTATTTACCAACTTTTTCCGGTGCAGCCCCAGCCCAGCACATCTGT
CGCCTATGGCCCCATGACTGGAAGTCCCCTTGATGAACATTTTAAATGGCATACAGCAA
ATACTGGCAGAGTTAGAGCACACACCAGCCAGCAACAGCCAGCTACAGGCATTTCTTC
AACTTGTCTTTTAGAGGATGCAAAGATGTTTCAAAAGAGCTGTTATTACTTCTCAAACA
CAAGTTTAGAATGGAATGGCAGTAAGAAATGGTGTGAAGACAAGAAAGCTCATTTAGT
AATAATCAAAAGTGAAGAAGAGCAGAAATTCCTAAAAGACGAAGTTACTAATTTTAGA
AACACCTTTATACAAAAACCTACAGGTCATTTTTTCTGGATTGGACTTACAGATAAATTA
AAGGAAGGAGAGTGGCGCTGGGTAGATGGCACTATCTGTGACTGCAACAATACTAACA
AAAATAAAAGATACTGGAGGGAAAAGGAGCCAGACAACTGGAATAATTTAGAGCACT
GTGCTGCACTTGCTGAGAATGGTGACTGGATGGATGGGAAATGTAACTGGACTCTCAGG
TGGATTTGTAAGATGGATGCTACGATTTTCTCTAGCTAATCAATTTTCTCAATGACAGTT
GTTTAAATGTGACACTGATGTTTGATCTAAACAACCATTGTAACAGTCTTGAACATGTTA
ATCATTCAATGATTATTTAATTATTATTTGGTTTGTTCATGAAAGACCCAAGTAATATTG
AGCACATGGACAATAATTCTGCTGTTGATGACTTTCTCCATTATTTCATGTATATGTTTAG
TGTTATATGCCTATATTCTAATCTCTAATGGATTCATATTAAAAGA

>CLEC4E-F_TRINITY_DN9366_c0_g1_i9
TAACTTTATTTGCAACTATTGTTTTTCAAGGCAATGATTTTTTTTTTTATTTTGACATCCAC
AAAAATAACCATAAATGAACGATGTGTACCATTAACTGAAATACCAAATGTAGTACAT
TATGTTGAATTAGTAGTAAACCGTTAAGAAAAAAAGATAAAGATGCCTTATTTGTCTTA
CCAAGCTGACTGTGTAGAAGTCAAACACTTTATAAAGGACAGTGCCAACATGTTTTGCA
TTATTATCCCTTACTTAGGAAAATCACAAAGAAATTAAGTGAATTTATGACAAGCTACA
TCTGGTTGGTTTTTCACAAATAAAATGAAACAAGGACTGGCAGTCTTCATCGTTCCATTT
TGACTGACAAGAATCCCACATGATCGCACAGTTTTCACTATTGGGATTGTTACCCCATG
CATTATTAGGTTCATTCTTGCACCAATGTCCAGATGATTTACTTTGAATTGTTCCATCTAC
CCAACGCCACACACCTTCCTCAATTTCATCAGTCAGTCCAATCCAGTAATCATTTTGGTT
GTTTCTTATAAAGAAAGTCTGTTCTTCAGCATTTGTGATCACCACTAATTCTGATGACAG
ATCTTGGCACATTCTCCTGGCAGTAGCCCAAGTTACAGACTTTTTGTAGTAGAAGTAAC
AGCTCAGCCTAAATTGCTTCCAGTCTGAAGGGCAAGGTTCAATCTGCTTTCTTTTAGAGA
GTTCTGTGAATTCAGTATTTTTTTGTCTGATCTGCCTTTGAAGGTTTTCCGTTTGCGTTAAC
ATGCTTTGAAAGCTGTGATTCAATAAGAGAAGGTCCTGGTTTGCTTGTTCTAGTTGTTTT
GACTTCTGTAAATATAGAATAGTCATGAAGATGCCAATTGCCACCAGGGATAAAATGA
GCATCAGGATGAATAACACCTTACAGTTCCACTTTGTTTTCTTCTTTGTGCTGTTAGGTTC
CTGCATGCCTTCTTTGGCACTTCGTAAAATTTCTGCTTGGGGATTGTTAGTTTGTTGCATA
TTAATTCTAGAATAAATGTTTTCATTTCTACATTGAAGCTCCTCATATGTTGTATTTTTTTC
CATGATCTTCAAAAATTTTAGAAACACCTGAAACTGACAGAGGAGCAGCGAAAGGCTG
TGTACCAGCATTTACAATGAAGAGGGAACCTGTGAGGCACGTTCCCCAGTAGAACAAA
TAAGTAGTGTTAAATCCAGAAGAATACGTAAAACAAACTAGTTCCTTAAAGAGTTCGTC
TTTTATTAAACAAATGCTGATTAAAAAAAGGTAGTTTAGCAC

>CLEC4E-G_TRINITY_DN383_c0_g1_i3
TCAACGAAAGGTGACAGCACTAATAACAAGACTTATTTATCAAATTTATTTTCAAGTGG
TGGTAGTTTGTGTCATCACTGAGCATCATGGAATACGAGAAGACATACCAGGAGCTTAA
GGTTGCAACTGAAGACACTTACTGTGAACTCAATCAACAACAAACCAAGAAACTCAGA
GCACAAAAGACAGCTCCAGAAACACAAAAATATGTGGCTAAGTATTGCAAACAACCA
AGAAAATTGTTAATCACCTCCATTATCACAGTGTTCATCCTGCTGGCAGTAATCATAGCT
GTGATTATTTGCAGTAAGATAATTCCTGAACCTGCTGTTTTAAAAGGCAATTCTTCAACT

TGTCTTTTAGATCATGCAAAGACGTTTCAAAACAGCTGTTATTACTTCTCAAACAGAAAT
TTAGAATGGAACGACAGTAAGAAATGGTGTGAAAACAGAAAAGCTCATTTAGTAATAA
TCAAAAGTGAAGAAGAGCAGAAATTCCTAAAAGACGAAGTTACTAATTATGGAAACA
CCTTTATACGAAAACCTAAAGGTCATTTTTTCTGGATTGGACTTACAGATGAAGTAAAG
GAAGGAGAGTGGCGCTGGATAGATGGCACTAACTGTGACTGCAATAGTAATAACAAAA
ATAAGGGATACTGGAGGGAAAATGAGCCAGACAACTGGAATAATTTAGAGCACTGTGC
TGCACTTGCTGAGAATGGTGACTGGATGGATGGGAAATGTAACTGGACTCTCAGGTGG
ATTTGTAAGATGGATGCTACGATTTTCTCTAGCTAATCAATTTTCTCAATGACAGTTGTTT
AAATGTGACACTGATGTTTGATCTAAACAACCATTGTAACAGTCTTGAACATGTTAATC
ATTCAATGATTATTTAATTATTATTTGGTTTGTTCATGAAAGACCCAAGTAATATTGAGC
ACATGGACAATAATTCTGCTGTTGATGACTTTCTCCATTATTTCATGTATATGTTTAGTGT
TATATGCCTATATTCTAATCTCTAATGGATTCATATTAAAAGA

>CLEC4M-A_TRINITY_DN1982_c0_g1_i1
ATGCATGATAGCCATGCTTGGATATAGTTTCAGTTCCAGTGAGGAAAAGGTTTCATTCA
TATTTACAAATACCTGGTTTACAAAATCTTCTTACATTTCTGCTTATACCAATGGTAATA
ATAATAATACTAGCCAAATCAGGGACATGAAAGCATTTCTGTAGGTCTTTGGTCTTCCC
AATGAATGCAAACCATTAAATAGAGTTATACAGGACAAAATGAAAATAGCACGTGAA
AGAAATGTTTATTTTCCCACTCAGCCTTTCTTTTCAAAATATAAAAGGAATTAAAGTTTT
TGTATTACTCTTAGTGAAAAAATAGTTTACTGTTTGACATTTAACATAATGCTCCATGGC
AATGCCTAGCCTTCACAGTCGGGGTCTGAGACTGAAGTCTTTGCAGCTCGCATTTATAA
ATAGCCTTTACAAAGAGCAAACAGAAGTCGGTGCCTCACAAATAAAGCGTAATACGCT
GGAACAGCGAGAATCACTCCATCTACCTTCAGATGTCATTAAGGCACAGTCTTCGTTAT
ACCAGTCATCAGGCTCACCGCTAACCCAAAATTGTTTCTGTTGTAGTTGCATTCACAAA
AAGTGCCATCTACCCATTTCCACGTATCCTCTTTTTCGTTATCTGTAAGCCCAATCCAGT
ACCACAGTTCACGTTCATATCTGGGTTGCTTATTTAACTCTTTTGCTCTCTTATACAAAAA
GTCCTGCTCCTCAGTACTTTTGATCACCACTAAATCCGATGACCAGTCTTTACATGTACT
CCTTGAATTTGTCCAGCTTTCCCTAGACGCTGAGAAGTAGTAACAACTTTGTTTGAATAT
CTTCCAGCTTGCAGGGCAACATTCAACCTCCCTTCCTTGGTTTGATGATGCAGCAATTTT
GTTCAACATATTCAGTTTATTTTCTATTTCCTTATATTTCTCTTCTGTCCTGTTTTCTTGCTT
ATCCATTTGGTGTTGGATTGTGATGTTAATTTTTTTTTCAAGCTGAAACAGAAGGGCTTC
CTTACTTCTTTGGAGTTCTGTAATATTATCTTCTGTCTCATGGACCTTCATCTGTAGGTCT
GTCTGATTAAATGCCAAGATGGTGATTTTCTCTTCTGCTTCCTTAAATTTACTGTAAACA
CTGAAATTCAATGACTGCAACTCGTGATTTGCTGGCCCATATTGTTTTATTGGCTGTAAA
TGTAAAACAGCCACAGTTATGACCACTGTGAGTAGGACGATAATGACAAATACCATAA
AGAACGGCATCACTCTGCTTGTCAGTGTTTTCTTGTTTACATCCTTAAATTTCTGCGATCC
TTGCTTGTTTGTTTTCTCTAGCTGTGTGTTCACAACTGGAGTGCCATGTCGATTGAAGCTC
ACTTCAGCATATGTTATTTCGTCTTGCATGTTGGTTATTCCAAGTTCTTTTTGTTTTACTGC
TTTAATTTTATTTTGCCTTTCCTTTCAAAGCAAGTTCTATGTTTCCATATACTGACAGGTTT
TAGATGACTCTCTGTGTTTGCTGTGATCTCTTCTTTTGTCTTATTTCCCTTTTGCTGTCTCCT
GCTTACTGTCTATATTTTTCTCAAGTTCTTACTCATGTTTCCTGAACTTTTTTTTTAATCTAT
TTGCTAAATGTTATCTCACCCCACCTTCCTTCCATCCGTCTCTCTCTCTCTTGCTTC

>CLEC4M-B_TRINITY_DN1982_c0_g1_i4
ATGCATGATAGCCATGCTTGGATATAGTTTCAGTTCCAGTGAGGAAAAGGTTTCATTCA
TATTTACAAATACCTGGTTTACAAAATCTTCTTACATTTCTGCTTATACCAATGGTAATA
ATAATACTAGCCAAATCAGGGACATGAAAGCATTTCTGTAGGTCTTTGGTCTTCCCAAT
GAATGCAAACCATTAAATAGAGTTATACAGGACAAAATGAAAATAGCACGTGAAAGA
AATGTTTATTTTCCCACTCAGCCTTTCTTTTCAAAATATAAAAGGAATTAAAGTTTTTGTA
TTACTCTTAGTGAAAAAATAGTTTACTGTTTGACATTTAACATAATGCTCCATGGCAATG
CCTAGCCTTCACAGTCGGGGTCTGAGACTGAAGTCTTTGCAGCTCGCATTTATAAATAG

CCTTTACAAAGAGCAAACAGAAGTCGGTGCCTCACAAATAAAGCGTAATACGCTGGAA
CAGCGAGAATCACTCCATCTACCTTCAGATGTCATTAAGGCACAGTCTTCGTTATACCA
GTTATCAGGCTCACCACTGACCCAATATTGTTTTCTGTTGTAGTTGCATTCACAAAAAGT
GCCATCTACCCATTTCCACGTATCCTCTTTTTCGTTATCTGTAAGCCCAATCCAGTACCA
CAGTTCACGTTCATATCTGGGTTGCTTATTTAACTCTTTTGCTCTCTTATACAAAAAGTCC
TGCTCCTCAGTACTTTTGATCACCACTAAATCCGATGACCAGTCTTTACATGTACTCCTT
GAATTTGTCCAGCTTTCCCTAGACGCTGAGAAGTAGTAACAACTTTGTTTGAATATCTTC
CAGCTTGCAGGGCAACATTCAACCTCCCTTCGTAAAACAGCCACAGTTATGACCACTGT
GAGTAGGACGATAATGACAAATACCATAAAGAACGGCATCACTCTGCTTGTCAGTGTTT
TCTTGTTTACATCCTTAAATTTCTGCGATCCTTGCTTGTTTGTTTTCTCTAGCTGTGTGTTC
ACAACTGGAGTGCCATGTCGATTGAAGCTCACTTCAGCATATGTTATTTCGTCTTGCATG
TTGGTTATTCCAAGTTCTTTTTGTTTTACTGCTTTAATTTTATTTTGCCTTTCCTTTCAAAGC
AAGTTCTATGTTTCCATATACTGACAGGTTTTAGATGACTCTCTGTGTTTGCTGTGATCTC
TTCTTTTGTCTTATTTCCCTTTTGCTGTCTCCTGCTTACTGTCTATATTTTTCTCAAGTTCTT
ACTCATGTTTCCTGAACTTTTTTTTTAATCTATTTGCTAAATGTTATCTCACCCCACCTTCC
TTCCATCCGTCTCTCTCTCTCTTGCTTC

>CLEC19A-A_TRINITY_DN1706_c0_g2_i2
TATTAAATATCTTATTATCAAAAATTACAAAGTATATTTAGCACTTCAAATCATCAAATA
CAGAATGTAGGAATAAACATTTAAGAAAGAGAAATGCAGTACTAATAAAACAAGTAA
AATCCAACAGTGAAGCCAATAAGAAACAACAGGTATATTTGATTTTGTATAAAACTGT
AAATTTTTTAAAATCATCTGCTGAAAACATTTCTTGCGATGTCCATGTAAGACTTCTCCT
TCATATTAGAATATTGGTGCGTATACAGAGCATCTTCGAAGGTCATATTTGCAGACAAA
AGGGAAAGACCATCTGCTACTGTAGTCATTCCAGGTAATAGTACCATTCTGGAAATACC
ATGACCCAACATAATCTTCATTGCCAAGATTGTCTGGTTGGGATTTGCCAAAACGTAGG
AAGTTCACTGGACTTCCATCAGTCCACATAAAAACACCCTCTACAAAAGTGTCATGTAA
TCCAATCCAGTAAGCCTGTCCTTTTATGTAATCATTGGGTCTACCCATTAGTTCATAAAT
GTAGTTATTTTCTTCAAGACTGTGGATGGATGCCAAATGAGCTCCGTAGTAGTTTCTTTG
GCAGTAGGCCTCAGCATTAACCCATGTCATTTTTATTTGTATGGGACGAAAGCATGAAT
TCTTGTACTGGAACCATCCAAATGAGCAGTCCCCCTGATAACAGAGACAGCCATCTGCA
GTGTCACCACCAGGAATAAAGGCAGCCAGGAGTACAGACATCAGTATCAGGTAATTCA
TCTTTACAATGAATACGTCAAGTATGATGAACTTTTTTGGCAA

>CLEC19A-B_TRINITY_DN1706_c0_g1_i1
TTATAACTTTGCCAAAAAAGTTCATCATACTTGACGTATTCATTGTAAAGATGAATTACC
TGATACTGATGTCTGTACTCCTGGCTGCCTTTATTCCTGGTGGTGACACTGCAGATGGCT
GTCTCTGTTATCAGGGGGACTGCTCATTTGGATGGTTCCAGTACAAGAATTCATGCTTTC
GTCCCATACAAATAAAAATGACATGGGTTAATGCTGAGGCCTACTGCCAAAGAAACTA
CTACGGAGCTCATTTGGCATCCATCCACAGTCTTGAAGAAAATAACTACATTTATGAAC
TAATGGGTAGACCCAATGATTACATAAAAGGACAGGCTTACTGGATTGGATTACATGA
CACTTTTGTAGAGGGTGTTTTTATGTGGACTGATGGAAGTCCAGTGAACTTCCTACGTTT
TGGCAAATCCCAACCAGACAATCTTGGCAATGAAGATTATGTTGGGTCATGGTATTTCC
AGAATGGTACTATTACCTGGAATGACTACAATAGCATATGGTCTTTCCCTTTTGTCTGCA
AATATGACCTTCGAAGATGCTATGTCTACAGACCAATATTCTAATATGAAAGAGAAGTC
TTACATGGACATCGCAAGAAATGTTTTCAGTAGATGATTTGAAAAAAATTACAGCTTTA
TACAAAATCAAGTATACCTGTTGTTTCTTATTACATGTGATTTTATGGTTGGGTACAACC
TTTTTATTATTACTGCATTTCTCTTTCTTATATGTTTATCCCTACATTCTGCATTTGATGATT
TGAAGCTTTACATATGTACTGTAATTTTTGATGAAAG

>CLEC19A-C_TRINITY_DN14306_c0_g2_i2

AAAATATTGCACACCATTGTGTTCATTAGAAAGATGAATTACCTGATACTGATGTCAGC
TATCCTGGTCATCTTTATTCCTCAGGGTGACATGCAAATCAACCATTGCCTCTGTGTTCG
TGGGATCTGCCCATATGGATGGTACCAGTACACAGATAATTGCTACAAAGTTATAACTA
CAAAAATGAGTTGGGTGAATGCTGAGGCCTTCTGCCAGAGTAGCTACCTTGGGGCTCAT
TTGGCATCCATCCACAGTGCTGAAGAAAATGATTACATTTTCACACTGACTGGGAACCT
ATCTAATTACCAAACAGGAAATGCATACTGGATTGGAGCCCATGACACTATTGTAGAA
GGCACCTACATGTGGACAGATGGAAGTGTGACAGACTACTTGCATTTTGGAACTGGTCA
GCCAGATAACCTTGGCAATGAAGATTATATTGGATCGTGGCATTTTCAAAATAATGCTA
TTACCTGGAATGACTACCCAGAATCTTTTTCCTTCCCTTTTGTCTGCAGGATCAGCCTAA
ATAGATGCTGTCGTTCTTGAATGAAGAGTTGCAGACTGGCAGCCACGTATGACAAGTCT
CCTGAGAACATCACAAATGTTATGGCTCCCATCCAAGGAATTATATAATTTCTTTTCAA
ATTATTTGCATCTATTGTTTGTACGATTATCCTATTCTTTTATTAAAGGTGTTTTTTTGGT
ACTATTCAATTTTATGACAATGGCCTGCTGAAATGAAATCCACTAGCTGTATCACTTCA
ACAGACATTTATTCAGGTATATGAGTCAATGAACTCCTCTGGCCATCTTTCACTTGGTAT
GCATTTTGTCAGTCCTCAAAATAATAAAAAGGAATTTGAAGCTCAACCCTCAATAATAC
CCTCTAAATACAGATTTTTTTTAACTCAGAAAGTATAGTCAGTGTGAGTGAGCAAAGG
AGTTAACAGGCTTGTGCATTTGATTAAATGTCTACCAAAATCAAACTGTCAGTAGATGT
AAATGTAACATCCGCTTAAGAATTGCAGAACCATATTTTCTTTTTCTGCTCATGTTGTATT
GCTATTTTCTTGCAGGTATGTATGATCTGCTCTGATTGCATTGTGGAGGTTCTAGGCATTC
CATTCTATACTGTCATTATTGATATAACGTTGTCATTTGGGCTAAGTTGCTGAACAAATG
GTGTATGCTACAGGTCTTAATAATAATACAATTCA

>CLEC16A-A_TRINITY_DN2288_c0_g1_i1
GATTAGCGATGGCTGAAATTTTGGATATGTGGGAATGGTAATGTCACTTATTAAAGGAA
GATTTGGGTACTTCGAACGTGGTTGTTGGTTTTCAAAATGGGTAACGGAGACTAAAATC
CATTCAGGATGTTTGGCCGTTCTAGAAGCTGGATGGTTGGCGGTCCAGGAAGGTCTTCA
AAGAGCATTCACTCTTTGGACCACTTAAAAAATATGCATCACCTCTTAACTAAAAACAC
TACTGTCACTGATCACAACCGTGACCTGCTAGTAGAAACCATCCGTTCTATCACTGAAA
TTCTCATTTGGGGTGACCAGAATGACAGCTCAGTGTTTGATTTCTTTTTGGAGAAAAATA
TGTTTGCCTTCTTCTTGAATATTCTTCGGCAGAAATCTGGGCGCTATGTCTGTGTGCAGCT
GCTTCAGACCCTGAATATTTTGTTTGAGAACATCAGCCACGAAACATCCCTTTATTACTT
ACTGTCCAACAACCATGTGAATTCAATTATCGTTCACAAGTTTGACTTCTCTGATGAGGA
GATTATGGCATACTACATATCATTCCTGAAGACACTGTCATTGAAGCTCAACAACCATA
CAGTACACTTTTTTTACAATGAGCACACAAATGATTTTGCATTGTATACAGAGGCAATC
AAATTTTTTAATCATCCAGAAAGCATGGTCAGAATTGCTGTCAGGACCATCACTCTTAA
TGTTTATAAAGTGAACAACCAGCATATGCTACATTATATTAGAGACAAAACTGCTGCCC
CCTATTTCTCCAATCTGGTCTGGTTCATTGGGAGCCATGTGATAGAACTGGACAACTGTG
TCCAGACTGATGAAGAACACGCAAACAAGAGCAAGCTGAGTGACCTTGTTGCAGAGCA
CCTCGACCACTTGCATTACCTAAATGACATTCTGATTATCAACTGTGAATTTCTAAATGA
TGTGCTTACAGATCACCTGCTGAACAGACTGTTCCTGCCTTTGTATGTCTACTCCTTAGT
AACTCATGAAAAGTGTGGAGAGCTTCCAAAAATCAGCGCGCAGGTGTCACTCTATCTC
CTTTCACAGGTCTTTCTGATCATACACTACTCACCTTTGGTGAACTCCTTGGCTGATGTTA
TTTTGAACGGTGACCTTTCTGTGTTTTCTGCACAGACAGAGCAAGATGTACAGAAGAGC
ATGGCAAAGTCCAATATACGATGCTTCACAAAGCCTGCAGAGAGTCTTGAGAAATCAC
TTGAGATCAGTAAGCAGAGGGGGAGAAAAAAAGCACAGAAAAGGCCAAATTATAAG
AATGTGGGTGAGGAGGAGGAAGAGGAAAAACCTCCAGATGAAACTCTGGAAGATCCT
GACAAGGCAAAAGGTACAGACGGAGCTTCAAAAAGCAACAGGACTACTTTGGAGACA
GAAGAAATAGAGATGGTAATTATGGAGAGGTGTAAAATATCAAAAGCTGTGGTAATGG
AACAGAATACTACAGATGAAGAGAAGAGTGCTGCTGCAGCAGCTGGAATAGAGACTT
CACAAAACAGGCCATTTTTGGATGTGGTATATAATGCTCTGGACTGCTCGGGAGATGAT
TATTATGCATTGTTTGTCCTTTGTCTGCTCTATGCAGTGTCCCACAACAGAGGTATTGATT

CTACTCTGCTAGAAAAAATCCAGCTTCCATTTCGGCAAACAGCTGAGAAGACAGTATAT
AGTCATGTTCTTGCTGAGAGGCTAATCAGGATAACGATCAATGCTGCCCAGCCAGACA
GCAAGATTCGACTTGCAACTTTGGAACTTAGTTGTTTGCTTCTGAAACAGCTTGTAATTT
CAGACAGCGGCTGTGTTATAAAGGACATACACCTGGCTTGCCTGGAGGGGGAAGAAAT
TTTTCTTGATGTGTTTGAAGATGAATACAGAAGCATGATGAACAAACCACTGAATGTAG
AGTACCTGATGATGGATGCTTCCATTCTGCTGCCCCCCACTGGGACTCCGCTGACTGGTA
TTGATTTTGTTAAGAGACTTCCCTGTGGAGATGTCGAGCGAACACGAAGAGCAATAAG
AGTTTTTTTCATGTTGCGAGCTTTATCACTGGAACTGAGAGGAGAACTGGAGACTCAGC
TGCCCTTGACAAGGGAGGAAGATCTGATTAAAACTGAAGATGTTTTGGATCTTAATAAC
AGTGACTTGATTGCCTGCACAGTGATTGCAAAGGATGGAGGACAAGCTCAGCGATTTCT
GGCTGTTGATATTTACCAGATGAGTTTAGTTGAACCCGAGACCAAAAGACTTGGCTGGG
GTGTGGTGAAGTTTGCTGGACTTCTGCAGGATATGCAGGTCACTGGGGTGGAAGATGAC
AGCCGTGCGCTAAATATCACAATTCACAAGCCCACATCAAATCCTCATGCCAAACCATT
TCCCATTTTACAGGCTACATTTATCTTTTCTGATCATATCCGCTGCATAATTGCAAAGCA
GAGACTGGCAAAGGGCCGCATCCAGGCACGGCGTATGAAAATGCAGAGAATAGCAGC
ATTGCTAGATCTTCCTGTTCAGCCATCTCCTGAGGTAATGGGCTTTGGACATGTTACAAG
TGCAGCTACCCAGCATCTGCCTTTTCGATTTTATGACCAGTCAAGACGTGGATTAAGTG
ACAGTGCAGTTCAGCGTTCTGTGTTTGCTTCTGTTGACAAAGTTCCAGGTTTTGCTGTAG
CCCAGTGTGTAAACCAGCATACTTCTGCACCAGCATCCTCACCATCCCCTCCTTCCAGC
AGTAGCCCCAATGGCAGTGGGAGCACTGGTCATTGTGATTCAGTCACAGCTAGCACAA
CATCCACTCCTGCAGCCCAAAGTCCATCAGACGATCCAGCATCTTTGGAAGAGTCATCG
CTGAATCTGCCTGCGCAGGTGGAGTTTGTTGATGAGACTAGCTGTAGCACTCCAGGCCC
CAGCAAGAACTTCCAGAGGAACTCTGAGTCTGAACCAGCTAACCTGGCCCCAAGCCTT
ACCCCTTCTCAACAGCCAACCATATCATTACTGTCAGATGACAATACAGATGCACGCAG
TGTGGAGTCCTTGACACTTGTCCCACCTGCTGATCCACATTGCATCTTAAGTTTTAGCAA
CACTGTACAGTCCTCAGTGCTGTCTGAGTCAGCATTAACCAATGAGGGGTATCATGCTG
AGACTTTTGAACCAGAAACAAGTACAGAAGTGTGAACAGAACCCATTAGACTTTTTTCT
CTTCTTGGAATGGGAGACTCTGTGAACTTTCAATGACTTTTGATGCAAAACACTGCAAT
ATACCACGTTGTTCATGGTAGAAGTGAAATGCAAGCAACTGTCTTTTAATGTCTATGGG
AGAGATGTTGAAGGAAGACTGATGAAGCTGAATTTCTTCACTGATTAGTCCATATAATA
AAAGGAAAGTGCAGTCTCCCTGCTGATTGAACAAAATTTCCAGGTGTTATTTATTCACA
CAGCTTAGTTCTGCTATTTTCACAGTGTTGAACAATGACTTGAAGGTGGACAGCTGAAG
GAGGTCTTCTTTTCTTCCCCATTTTTATTGTCTGCCGCCAAGTTTTGTTAGACTAGGCTTTT
GTATCTGCAGGTTGTCAGTCACTACAAGTCCATGTGATGTGTGAAAATACCATTTGTCAT
CTGATCTAATGGAGTCAGGATCGTAGTTTTGAAATAATGGCCTGCCTAAATAAAATCAT
TTACCATGATCTTCCAAACAAGTTGTATCTTGTACACTAAAATTGCACTGGATGTTTAGG
GACCAGTATAATTTTGTGAAACATACGTTCTTATTTATTCCTTTTGTTGTAGTTATAAATA
TGGGTTATGTTAAAAATGTTAAAGTGGCCTACAGTAATATAGCAGATGCATTATGCCAT
GTGTTTGCATTCAGTTAGATGCATTTTCCCTGTATTTAGTGCAGATTTAG

>CLEC16A-B_TRINITY_DN2288_c0_g2_i2
CTAAATCTGCACTAAATACAGGGAAAATGCATCTAACTGAATGCAAACACATGGCATA
ATGCATCTGCTATATTACTGTAGGCCACTTTAACATTTTTAACATAACCCATATTTATAA
CTACAACAAAAGGAATAAATAAGAACGTATGTTTCACAAAATTATACTGGTCCCTAAA
CATCCAGTGCAATTTTAGTGTACAAGATACAACTTGTTTGGAAGATCATGGTAAATGAT
TTTATTTAGGCAGGCCATTATTTCAAAACTACGATCCTGACTCCATTAGATCAGATGACA
AATGGTATTTTCACACATCACATGGACTTGTAGTGACTGACAACCTGCAGATACAAAAG
CCTAGTCTAACAAAACTTGGCGGCAGACAATAAAAATGGGGAAGAAAAGAAGACCTC
CTTCAGCTGTCCACCTTCAAGTCATTGTTCAACACTGTGAAAATAGCAGAACTAAGCTG
TGTGAATAAATAACACCTGGAAATTTTGTTCAATCAGCAGGGAGACTGCACTTTCCTTTT
ATTATATGGACTAATCAGTGAAGAAATTCAGCTTCATCAGTCTTCCTTCAACATCTCTCC

CATAGACATTAAAAGACAGTTGCTTGCATTTCACTTCTACCATGAACAACGTGGTATAT
TGCAGTGTTTTGCATCAAAAGTCATTGAAAGTTCACAGAGTCTCCCATTCCAAGAAGAG
AAAAAAGTCTAATGGGTTCTGTTCACACTTCTGTACTTGTTTCTGGTTCAAAAGTCTCAG
CATGATACCCCTCATTGGTTAATGCTGACTCAGACAGCACTGAGGACTGTACAGTGTTG
CTAAAACTTAAGATGCAATGTGGATCAGCAGGTGGGACAAGTGTCAAGGACTCCACAC
TGCGTGCATCTGTATTGTCATCTGACAGTAATGATATGGTTGGCTGTTGAGAAGGGGTA
AGGCTTGGGGCCAGGTTAGCTGGTTCAGACTCAGAGTTCCTCTGGAAGTTCTTGCTGGG
GCCTGGAGTGCTACAGCTAGTCTCATCAACAAACTCCACCTGCGCAGGCAGATTCAGC
GATGACTCTTCCAAAGATGCTGGATCGTCTGTTTCCTGCTTTGTAAGAGCTGCTGCTGCT
TGAAGACCTAAACCCAGTGTCCACCATGACTGTCAGCTTCCGACAGACTAAAAACTCG
CCGCCGTCCTTTCCTGCCAGACCTTACTGATGGACTTTGGGCTGCAGGAGTGGATGTTGT
GCTAGCTGTGACTGAATCACAATGACCAGTGCTCCCACTGCCATTGGGGCTACTGCTGG
AAGGAGGGGATGGTGAGGATGCTGGTGCAGAAGTATGCTGGTTTACACACTGGGCTAC
AGCAAAACCTGGAACTTTGTCAACAGAAGCAAACACAGAACGCTGAACTGCACTGTCA
CTTAATCCACGTCTTGACTGGTCATAAAATCGAAAAGGCAGATGCTGGGTAGCTGCACT
TGTAACATGTCCAAAGCCCATTACCTCAGGAGATGGCTGAACAGGAAGATCTAGCAAT
GCTGCTATTCTCTGCATTTTCATACGCCGTGCCTGGATGCGGCCCTTTGCCAGTCTCTGCT
TTGCAATTATGCAGCGGATATGATCAGAAAAGATAAATGTAGCCTGTAAAATGGGAAA
TGGTTTGGCATGAGGATTTGATGTGGGCTTGTGAATTGTGATATTTAGCGCACGGCTGTC
ATCTTCCACCCCAGTGACCTGCATATCCTGCAGAAGTCCAGCAAACTTCACCACACCCC
AGCCAAGTCTTTTGGTCTCGGGTTCAACTAAACTCATCTGGTAAATATCAACAGCCAGA
AATCGCTGAGCTTGTCCTCCATCCTTTGCAATCACTGTGCAGGCAATCAAGTCACTGTTA
TTAAGATCCAAAACATCTTCAGTTTTAATCAGATCTTCCTCCCTTGTCAAGGGCAGCTGA
GTCTCCAGTTCTCCTCTCAGTTCCAGTGATAAAGCTCGCAACATGAAAAAAACTCTTATT
GCTCTTCGTGTTCGCTCGACATCTCCACAGGGAAGTCTCTTAACAAAATCAATACCAGT
CAGCGGAGTCCCAGTGGGGGGCAGCAGAATGGAAGCATCCATCATCAGGTACTCTACA
TTCAGTGGTTTGTTCATCATGCTTCTGTATTCATCTTCAAACACATCAAGAAAAATTTCTT
CCCCCTTATAAAAGCGTCGTAAGAGATGGACACTTTCTTCCCTTGCACCCTCCAGGCAA
GCCAGGTGTATGTCCTTTATAACACAGCCGCTGTCTGAAATTACAAGCTGTTTCAGAAG
CAAACAACTAAGTTCCAAAGTTGCAAGTCGAATCTTGCTGTCTGGCTGGGCAGCATTGA
TCGTTATCCTGATTAGCCTCTCAGCAAGAACATGACTATATACTGTCTTCTCAGCTGTTT
GCCGAAATGGAAGCTGGATTTTTTCTAGCAGAGTAGAATCAATACCTCTGTTGTGGGAC
ACTGCATAGAGCAGACAAAGGACAAACAATGCATAATAATCATCTCCCGAGCAGTCCA
GAGCATTATATACCACATCCAAAAATGGCCTGTTTTGTGAAGTCTCTATTCCAGCTGCTG
CAGCAGCACTCTTCTCTTCATCTGTAGTATTCTGTTCCATTACCACAGCTTTTGATATTTT
ACACCTCTCCATAATTACCATCTCTATTTCTTCTGTCTCCAAAGTAGTCCTGTTGCTTTTT
GAAGCTCCGTCTGTACCTTTTGCCTTGTCAGGATCTTCCAGAGTTTCATCTGGAGGTTTTT
CCTCTTCCTCCTCCTCACCCACATTCTTATAATTTGGCCTTTTCTGTGCTTTTTTTCTCCCC
CTCTGCTTACTGATCTCAAGTGATTTCTCAAGACTCTCTGCAGGCTTTGTGAAGCATCGT
ATATTGGACTTTGCCATGCTCTTCTGTACATCTTGCTCTGTCTGTGCAGAAAACACAGAA
AGGTCACCGTTCAAAATAACATCAGCCAAGGAGTTCACCAAAGGTGAGTAGTGTATGA
TCAGAAAGACCTGTGAAAGGAGATAGAGTGACACCTGCGCGCTGATTTTTGGAAGCTC
TCCACACTTTTCATGAGTTACTAAGGAGTAGACATACAAAGGCAGGAACAGTCTGTTCA
GCAGGTGATCTGTAAGCACATCATTTAGAAATTCACAGTTGATAATCAGAATGTCATTT
AGGTAATGCAAGTGGTCGAGGTGCTCTGCAACAAGGTCACTCAGCTTGCTCTTGTTTGC
GTGTTCTTCATCAGTCTGGACACAGTTGTCCAGTTCTATCACATGGCTCCCAATGAACCA
GACCAGATTGGAGAAATAGGGGGCAGCAGTTTTGTCTCTAATATAATGTAGCATATGCT
GGTTGTTCACTTTATAAACATTAAGAGTGATGGTCCTGACAGCAATTCTGACCATGCTTT
CTGGATGATTAAAAAATTTGATTGCCTCTGTATACAATGCAAAATCATTTGTGTGCTCAT
TGTAAAAAAAGTGTACTGTATGGTTGTTGAGCTTCAATGACAGTGTCTTCAGGAATGAT
ATGTAGTATGCCATAATCTCCTCATCAGAGAAGTCAAACTTGTGAACGATAATTGAATT

CACATGGTTGTTGGACAGTAAGTAATAAAGGGATGTTTCGTGGCTGATGTTCTCAAACA
AAATATTCAGGGTCTGAAGCAGCTGCACACAGACATAGCGCCCAGATTTCTGCCGAAG
AATATTCAAGAAGAAGGCAAACATATTTTTCTCCAAAAAGAAATCAAACACTGAGCTG
TCATTCTGGTCACCCCAAATGAGAATTTCAGTGATAGAACGGATGGTTTCTACTAGCAG
GTCACGGTTGTGATCAGTGACAGTAGTGTTTTTAGTTAAGAGGTGATGCATATTTTTTAA
GTGGTCCAAAGAGTGAATGCTCTTTGAAGACCTTCCTGGACCGCCAACCATCCAGCTTC
TAGAACGGCCAAACATCCTGAATGGATTTTAGTCTCCGTTACCCATTTTGAAAACCAAC
AACCACGTTCGAAGTACCCAAATCTTCCTTTAATAAGTGACATTACCATTCCCACATAT
CCAAAATTTCAGCCATCGCTAATC

>CLEC16A-C_TRINITY_DN2288_c0_g1_i3
GATTAGCGATGGCTGAAATTTTGGATATGTGGGAATGGTAATGTCACTTATTAAAGGAA
GATTTGGGTACTTCGAACGTGGTTGTTGGTTTTCAAAATGGGTAACGGAGACTAAAATC
CATTCAGGATGTTTGGCCGTTCTAGAAGCTGGATGGTTGGCGGTCCAGGAAGGTCTTCA
AAGAGCATTCACTCTTTGGACCACTTAAAAAATATGCATCACCTCTTAACTAAAAACAC
TACTGTCACTGATCACAACCGTGACCTGCTAGTAGAAACCATCCGTTCTATCACTGAAA
TTCTCATTTGGGGTGACCAGAATGACAGCTCAGTGTTTGATTTCTTTTTGGAGAAAAATA
TGTTTGCCTTCTTCTTGAATATTCTTCGGCAGAAATCTGGGCGCTATGTCTGTGTGCAGCT
GCTTCAGACCCTGAATATTTTGTTTGAGAACATCAGCCACGAAACATCCCTTTATTACTT
ACTGTCCAACAACCATGTGAATTCAATTATCGTTCACAAGTTTGACTTCTCTGATGAGGA
GATTATGGCATACTACATATCATTCCTGAAGACACTGTCATTGAAGCTCAACAACCATA
CAGTACACTTTTTTTACAATGAGCACACAAATGATTTTGCATTGTATACAGAGGCAATC
AAATTTTTTAATCATCCAGAAAGCATGGTCAGAATTGCTGTCAGGACCATCACTCTTAA
TGTTTATAAAGTGAACAACCAGCATATGCTACATTATATTAGAGACAAAACTGCTGCCC
CCTATTTCTCCAATCTGGTCTGGTTCATTGGGAGCCATGTGATAGAACTGGACAACTGTG
TCCAGACTGATGAAGAACACGCAAACAAGAGCAAGCTGAGTGACCTTGTTGCAGAGCA
CCTCGACCACTTGCATTACCTAAATGACATTCTGATTATCAACTGTGAATTTCTAAATGA
TGTGCTTACAGATCACCTGCTGAACAGACTGTTCCTGCCTTTGTATGTCTACTCCTTAGT
AACTCATGAAAAGTGTGGAGAGCTTCCAAAAATCAGCGCGCAGGTGTCACTCTATCTC
CTTTCACAGGTCTTTCTGATCATACACTACTCACCTTTGGTGAACTCCTTGGCTGATGTTA
TTTTGAACGGTGACCTTTCTGTGTTTTCTGCACAGACAGAGCAAGATGTACAGAAGAGC
ATGGCAAAGTCCAATATACGATGCTTCACAAAGCCTGCAGAGAGTCTTGAGAAATCAC
TTGAGATCAGTAAGCAGAGGGGGAGAAAAAAAGCACAGAAAAGGCCAAATTATAAG
AATGTGGGTGAGGAGGAGGAAGAGGAAAAACCTCCAGATGAAACTCTGGAAGATCCT
GACAAGGCAAAAGGTACAGACGGAGCTTCAAAAAGCAACAGGACTACTTTGGAGACA
GAAGAAATAGAGATGGTAATTATGGAGAGGTGTAAAATATCAAAAGCTGTGGTAATGG
AACAGAATACTACAGATGAAGAGAAGAGTGCTGCTGCAGCAGCTGGAATAGAGACTT
CACAAAACAGGCCATTTTTGGATGTGGTATATAATGCTCTGGACTGCTCGGGAGATGAT
TATTATGCATTGTTTGTCCTTTGTCTGCTCTATGCAGTGTCCCACAACAGAGGTATTGATT
CTACTCTGCTAGAAAAAATCCAGCTTCCATTTCGGCAAACAGCTGAGAAGACAGTATAT
AGTCATGTTCTTGCTGAGAGGCTAATCAGGATAACGATCAATGCTGCCCAGCCAGACA
GCAAGATTCGACTTGCAACTTTGGAACTTAGTTGTTTGCTTCTGAAACAGCTTGTAATTT
CAGACAGCGGCTGTGTTATAAAGGACATACACCTGGCTTGCCTGGAGGGGGAAGAAAT
TTTTCTTGATGTGTTTGAAGATGAATACAGAAGCATGATGAACAAACCACTGAATGTAG
AGTACCTGATGATGGATGCTTCCATTCTGCTGCCCCCCACTGGGACTCCGCTGACTGGTA
TTGATTTTGTTAAGAGACTTCCCTGTGGAGATGTCGAGCGAACACGAAGAGCAATAAG
AGTTTTTTTTCATGTTGCGAGCTTTATCACTGGAACTGAGAGGAGAACTGGAGACTCAG
CTGCCCTTGACAAGGGAGGAAGATCTGATTAAAACTGAAGATGTTTTGGATCTTAATAA
CAGTGACTTGATTGCCTGCACAGTGATTGCAAAGGATGGAGGACAAGCTCAGCGATTTC
TGGCTGTTGATATTTACCAGATGAGTTTAGTTGAACCCGAGACCAAAAGACTTGGCTGG
GGTGTGGTGAAGTTTGCTGGACTTCTGCAGGATATGCAGGTCACTGGGGTGGAAGATGA

CAGCCGTGCGCTAAATATCACAATTCACAAGCCCACATCAAATCCTCATGCCAAACCA
TTTCCCATTTTACAGGCTACATTTATCTTTTCTGATCATATCCGCTGCATAATTGCAAAGC
AGAGACTGGCAAAGGGCCGCATCCAGGCACGGCGTATGAAAATGCAGAGAATAGCAG
CATTGCTAGATCTTCCTGTTCAGCCATCTCCTGAGGTAATGGGCTTTGGACATGTTACAA
GTGCAGCTACCCAGCATCTGCCTTTTCGATTTTATGACCAGTCAAGACGTGGATTAAGT
GACAGTGCAGTTCAGCGTTCTGTGTTTGCTTCTGTTGACAAAGTTCCAGGTTTTGCTGTA
GCCCAGTGTGTAAACCAGCATACTTCTGCACCAGCATCCTCACCATCCCCTCCTTCCAG
CAGTAGCCCCAATGGCAGTGGGAGCACTGGTCATTGTGATTCAGTCACAGCTAGCACA
ACATCCACTCCTGCAGCCCAAAGTCCATCAGTAAGGTCTGGCAGGAAAGGACGGCGGC
GAGTTTTTAGTCTGTCGGAAGCTGACAGTCATGGTGGACACTGGGTTTAGGTCTTCAAG
CAGCAGCAGCTCTTACAAAGCAGGAAACAGACGATCCAGCATCTTTGGAAGAGTCATC
GCTGAATCTGCCTGCGCAGGTGGAGTTTGTTGATGAGACTAGCTGTAGCACTCCAGGCC
CCAGCAAGAACTTCCAGAGGAACTCTGAGTCTGAACCAGCTAACCTGGCCCCAAGCCT
TACCCCTTCTCAACAGCCAACCATATCATTACTGTCAGATGACAATACAGATGCACGCA
GTGTGGAGTCCTTGACACTTGTCCCACCTGCTGATCCACATTGCATCTTAAGTTTTAGCA
ACACTGTACAGTCCTCAGTGCTGTCTGAGTCAGCATTAACCAATGAGGGGTATCATGCT
GAGACTTTTGAACCAGAAACAAGTACAGAAGTGTGAACAGAACCCATTAGACTTTTTTC
TCTTCTTGGAATGGGAGACTCTGTGAACTTTCAATGACTTTTGATGCAAAACACTGCAAT
ATACCACGTTGTTCATGGTAGAAGTGAAATGCAAGCAACTGTCTTTTAATGTCTATGGG
AGAGATGTTGAAGGAAGACTGATGAAGCTGAATTTCTTCACTGATTAGTCCATATAATA
AAAGGAAAGTGCAGTCTCCCTGCTGATTGAACAAAATTTCCAGGTGTTATTTATTCACA
CAGCTTAGTTCTGCTATTTTCACAGTGTTGAACAATGACTTGAAGGTGGACAGCTGAAG
GAGGTCTTCTTTTCTTCCCCATTTTTATTGTCTGCCGCCAAGTTTTGTTAGACTAGGCTTTT
GTATCTGCAGGTTGTCAGTCACTACAAGTCCATGTGATGTGTGAAAATACCATTTGTCAT
CTGATCTAATGGAGTCAGGATCGTAGTTTTGAAATAATGGCCTGCCTAAATAAAATCAT
TTACCATGATCTTCCAAACAAGTTGTATCTTGTACACTAAAATTGCACTGGATGTTTAGG
GACCAGTATAATTTTGTGAAACATACGTTCTTATTTATTCCTTTTGTTGTAGTTATAAATA
TGGGTTATGTTAAAAATGTTAAAGTGGCCTACAGTAATATAGCAGATGCATTATGCCAT
GTGTTTGCATTCAGTTAGATGCATTTTCCCTGTATTTAGTGCAGATTTAG

>KLRG1_TRINITY_DN6253_c0_g1_i1
TTTTTCAGAGTAAGAATTGTGCTTTTTTTGTAACATTTATTTTTTAGACAGTCCTACAATT
GTAACTGTAGTCCACTTTTAATGCAAGCTGCTAAATGCATCCTTTGTACATATGAAGTTG
AAGTCTTCTTCACACGATGAGGCTGAAAGTCCATGGGAGTTAATGACTAGACATCTCTG
TCCCACATAATTACTTCTCACAGGAAAAATGGTGGTATTCAGAGGAATTCCATCTTCCC
AAATCCACATGTCATTTTGTTTTGTGATCCCAATCCAGTGATATGTTTCTTTTGCTACTTT
GTTTGCAAAATTCATTGCTTCCTTTTGTTGTTCCTTAAATATCGATGAATGATGTGCTGAA
CAAAATTCTTTGGAAGAAGTCCAGTTCTTCTTTTTATCAGAAAATAGATAACATTTACTA
TGGTATCCAATCCATTTTTCTTGGCATACGAAGCACTTTCCTGTACCATTGCTTTCTGAAT
CTTTGCACTTCTGAGTTATCATCTTCCATTCAACTAACAGAAGAGTGAGGATTGACACTG
CCAACAAAAGGCACAATGTGGAAATGAGGAACATAATGCAACAACAATTGAAATTAG
ATGATTTAGGATCTTTCTCATTTCCAGAGTGCTGTTTTTGGTGTTCCAGTGTGCTGGGCTC
CTTGACTGTACCTCTGGCTGTTCTTGTGGTGGCTTTCTCATTTCCAGAGTGCTGTTTTTGGT
GTTCCAGTGTGCTGGGCTCCTTGACTGTACCTCTGGCTGTTCTTGTGGTGGCTCTTTCTTT
AGCTGTTGGCTCAATTTTTTTGAGTAAAACTGAATTAATGTATATATTTGCATCTTTCTTC
CTCACATCTTGTACAGCTTGATTATTCAGATTTTCAGCTTTTGTTTGTTGTTTAAATTCAG
ATATGTTGCAATAGATAACTCTGTTTTGTCCTTTCATGTTTTGTGAGCTTTCTTCTTCCAA
AGGTCCTGCTGTAAACTATGTTACTTTTTTAGCAGATAAGATGTATTTGTTTGTCGCTTTG
ATTTTCTTCCAGGTTGCTGAAGGTTCTACTTTCTGTTCTGCATTATTAGCATTAACTTTAC
ATTGCTAGTGACAGATGGTCAGTTCTATCCCCATA

>SELE-A_TRINITY_DN68820_c1_g1_i11
CTGGGATGAAACTGCAGTCGGCGGTTTCAGGGACATTTTGTAGCTGTTCGTTTCCAAAC
AGCTACTCGTTCAGCTGATCGAGGGGAACGCAGAGGGACTGATCAGCTGATTTTCCATG
CCACTTCCACCAGGGAACTTTCTTTACTACGTTGTTAGTCAGCAGCTCGACCTGAAGAG
GGAGCTGCTGACTTAAGTCCTGGGAGAATGCCACATTTGTCTCTGTGGCTTTTTTAAAT
CATGCAGTTCTCCCAGGCAAGTGAAGATATTTAACCTCTCCACCAGGAAGAAACTGCA
GTCGGCAGTTTCTGGGTGATACTAGCTGTTTTCAAAGAGCTACGCATTTCAAGCTGATT
TAGGGGAATGTGAACAGACTGATCAGCTGTTTCTTGGTGCCACTTCCACCAGGGAAACT
TTATTCACCTAGTAATAGTTACCACGCAAAAAAGACAAAGCAATGCAGAACCCTGTGC
GAATGAAGGCTTTTATTCGCAACGGATACTTAATGACATTTGATGAAGCTAAAGAATAC
TGTCAAACATACTACGATGGCCTGGCAGAGATCTATCAAGAATCAGAGAATGCTCAAA
TACCAGAATTTATTCATGAGAGACAGTGCTGGATAGGTACTGAAAACTGTCAGAGGGT
CTTTGCCACAAATTATACTGAATGGTGCTACAGCAGAAGTAAACGCAATGTCAGCTTTT
TACCCTGGAAGAAAACAAAACCCGAATACAACCATGTGCAAGGGACATATGCCTGTGT
CTTTATTAAAGATGATGCTATGTGGCATCTTGAACCATGCAAAACAAAGAAAACATCA
CTCTGTCAAAAATTGCACTGTGGTACCCAAAGCTGTAGCGGACGAGGCAGATGCATGG
AGGAGCTGAACCTCTACCAGTGCAAGTGCTTTCCAGGGTTCAGTGGTCCTAACTGTGAG
ACTGTGACACACTGCCCAAAGCTTGCTCCTCCAGATGATGCAGAAGTGAGGTTTGTAGT
GGGAAGCCATGGACCTCTGGCTTTTTCTGCGGTGTGGAATTTTAGCTGTATAGAAGGATT
TATGCTGAATGGAACCTCACAGACTACTTGTCTACCTTCTGGATTGTGGAGTCACTACTC
ATTTCAGTGTGTGGCTAAGACAGATTATCCTTCCAACCTTACGGAAGCTTGTGTTGCTGC
TGCCACTGCTGGGAGTTCCATTTTGATATCTATCACTGTGTTCTTCATTTGGAAGCATTAC
TCTGCAGGAGGTGCGTGTAAAAACTTTAGAAACACTGAGGCTGACTACCAGTGTCTGCT
GCTAGAAGAAGTAGAGAGAGATTTGGAAAATACAGTCTTGTGAGCACTGTAGAATTAA
ATGCATGTTAAACAATGCTGAACTTGTCACTACCTTGTCAGACTTAAAAATGAATCATT
ACACATTCCTGTCTCTTGAAAAGTAATGTATTTATCCCATATTCATTAATTTCTGTTCTAC
CCTGACAGAGCAAGTGAGGCACACTTGGTGTAGTTGCAGGCAGCACTAATAGTTATGA
TTTGCCTTTACCAGAAAACAGATTCCAAGCATATATATGGCATGCTGTTGGGCATAATG
AATCCTCAAACAAGCATCCACGATTCCATATTTTGCATAGTTAGATACCCGTGAACAAA
AATGCAATGTACTTTGTCATTAGTTTAAGATTGCTGTAAGTGGCATGTAGACACACTGCT
CAAGAAGTAGTAATCCCAGTAAATGCATGTACTTGCTGCAGAGAGAATGAAACTACTT
TTGAAGATCTGTAATACATTACATTTCTCTACAGGCTTTCCCCTGATGTGCCCCTGAATT
CAATATCTGTATCTGTTCTAAACATGTCAGTGTTTGTACTGATTATTGCAATTGTTATTCA
CTGTGCTTTTTATTTTGAGAGTGAGTTAAACTGCTAGAATTGCTTGGAATATCAGTTGTA
TATCCATGCACTGCTTATAGTTTCTTGTAGATTTAAATATGTGAAAAGTTTAATGGGAGT
TAAGAATCACCTGCCTGGACTGTCTGACCGTTATGTTCATGTTTGTCCATAATCACTCTG
ATACTTTATACAATAGTGTAGCTATTATTTTGTAGTATACTAAAACAGTGCAACATCAGC
AGCACTTTTGAATGCTTAACAAAGTTGTTTTGTAACCCTAATAAATCAGATGGTGTGATG
GACTGTCTTTGTATCAGGGGTAAAAGCAGTGCTGATAGCAAATGTTTATGTCAATCATC
ATATATGCCTGCTTTGTGGCACAAAATAAAAGTTTAAGAAGTATATTTCTTCTGAAT

>CD209_TRINITY_DN7436_c0_g4_i1
AAGTTGCTCATTAACACTTCCACATGAGAATTGAAAAAGAATATTTAAGACATTTGAAC
AACTTCAGACAAACTGAAAAACAACTGAAATGACAGCAAGACTACAGAAAGCAGCAA
AAGAAGAAATGATGAAGCGTGGAAAAGGATATTCAGTGCTGTTGATTATTTCCACTATC
ATCCTTGTCGCTCTGCTGGTCATAAATGTAATCTTATTGCTTTTAACTATACGGATGTCAG
AAGAGTTAAGTAAGTGCATGAATAGCACACATCCAACTGAGCACCCAAAAACTACAGC
AATGACAACAACAACAACAACAACAACAACAACAACAACAGCAGCAACAACTGCACCTTT
TACTAGCTGTAACCCAGATGAAATACGTTTTAACAACAGCTGTTATTTCTTCTCCTCCAA
CCTACATAATACCTGGAGTGAAAGCCAGGAATATTGTAAAAAGAAGAATTCTGATTTA
GTTGTAATCAAAAGTGCTGAAAAACAGCAATTCATGAAACTGAACACTGCAAAGGATG

GTGGATTCTATTGGCTTGGACTACAATATGCTGGTGCACAAACGTGGAAATGGGTGGAT
GGGACTATTTGCGATGTCCATGACAAGAAAACAACATATTTTGTTGATGGAAAACCTAG
CCCCAGTGAACCAGATAAAGACTGTGTCTACATGACATATCCCTCAGGACTATGGAAT
ACTGAGAATTGTGGTTTCAATTACCGATGGGCATGTGAGAGAAAGATAACTCACTGATG
AACAACATTATCTAGGAAGTCAATAGCATTATCTGCAACTAATCATGATGGCAAGGGTT
TTGAAGTTCAGAAGAAAATGCTGCCAGACAAAAATGTGAAGCTTGGTGATTGTGAATA
AGAGCTCTGTGATCGAATGCTATAACATTTAATTGCCCATTGATTTTAACATGGCAATTC
CTGATAATCATATGGAAGCATGAATTATTCTAATGATGTCTGGTTCTGTTTTCTCACTCA
GATATTTGAAATATAAAACAGCCTTGTTTGATTTCTTCTCAATGCATTTTAAGAATAAAC
TAAGCTTTCATGACTCTAAATATAATGTTTTCATGCTCTCTTGTTTTCTCTTTGTATAATA
GGAACCAACAGACCAGAGCCTCCCATGTGGATGCAGTGGGCGGATGGTTGGGCAGCA
CATGTGGACATTGATCACACACTAGGAATGGAGGTGCTGCAGTGGCCACAAATCTTCTT
AG

>CD302_TRINITY_DN8442_c0_g1_i6
ATATGGGACGTGAGTTGGCCCTCTTTTTTTTTTTTTTTTTTTTTTTTTTTTTTTTTTGCTAT
GAATGGATTTTCTTTCATTTCTTGGATACCTTATAGACCAAGACTGAGAAGAAGTTTGCA
AAGCCTACCCACTTGCCTTAAGTCATTCAGAAGACAGTATTACATGGAACTAACAATCC
TTCCTTCATGTTGTGGAATACAGGAACATGAACACTTTCTTCTTTCAGGGTGGCCTGCAC
AGGGAGGTTCATTTCTGAACCTGCTATCAGAAGTGTTAAGGGTACCAAAAACTCTCAGA
CAACTGTACCACAAACACCTCTGAGAAATTAATCAGCTCAGAGACAACTGTAACACAA
CACCTCTAGAAACACATTTTCAGACGTCATTTACCTTACAGAGGTGATGCACAAATTAC
CTTTTTCTGATCCACTGAAAGAAGATATGTTGTGACGAACAACTTTTTTTTCTTCTTTTGG
TGCTGGTTCAGAACATGCATTTTCTTAATTATGTTGCAACAAACATCTGCAGCATATTTC
TGTGTGAGAAAATATTTAATTCTGTACTTTAATACCTTTAAGCTGCTACACAGCTAAAAC
CCATGAGTCCTGAAAATAACAGTGGAAAGTCTGACAAAAGACTCTACAGGTGAACTTT
TCACAAATGCATTCAGACTTTTGAAAGAAGGTAATAAACAGATCACTTTTTAGCACATT
CTACATCCTGATAAGTAACATTTCTATGGCTTTATATAGTTAGTACAAATACTGAAAGG
ATAAATATTTTCTTGTGGTTTTCAGATACAGACTTTTAAACTGTTATATATCCTGTGTTAC
ATGTTCAACTTCCTCAGAGTCTAATAAAATGTGTTCATCATGTGGAGTTCGGGCTGCTGG
TTGATAATCAAGTGATGACGATCCCAATGAGGTCTGTTTTCGTTCACACAGGAACCAAA
ACACTACTGATGAGATACTCACGAATGTGACAGACATGATAACCAGGACAGTAATCAA
AGCCTTCTTGTCACCTTGCTCCAGTGTCCAGATTTGATATGAATGACGGCACATATCTCG
GCAAGAGGCAAATCTTCACTGTATTGTTTAGTCCAGTTCGAATATGACACTTCTGAGTTG
TCAAACCATTTCAAACTATCCGTGTCTGTATCATAAAACATGCCAAGCCAAACTTCTTC
AGGTCCCTGCCAATTTGTCCTGTAAGTGTTTACTAAAAACACATTTTCTTCCTCACTGTTC
ACACTCACAAGGTCAGCACTGGAAGCTGAAGCTCTGCAGAGTTCCCTTGCATCTTCTAT
ACCAAGGGAATGTTTTGAAAATTCATCCAAAACAGTATAACATCTGTTCTTATACGATA
TCCATGAAATCTTAGTCCCAGATGGACATTCTAAACACATAACGGAAGGGCAATGGAA
TACAAAATAAAAACAGCCCCAACAAAACAGCAGTGCAGATGCCTTTTTCACTTTCTGC
ATGTTTATATTTCATAGTCTAAAACATTACTCGTACTCCACAACGTTTTGGTCTGCAAC

>CD22_TRINITY_DN22556_c1_g1_i5
TATATATATATATATATATATATATATATATATATATACATACATATATAAAATCCAGTTTTCA
CAGGGTGAATCAAATGAACCTTATGATGTAATTCAGAGCTGCTAGGACACCTGTATTTC
ACAGCTGTAGTTATTTTTGAGGTCACCTTGTTGTGTCAGCAACTGTTTAAAAGAAATAAT
GAACAGATAATTTCCACCCACTGAGAAACATATGATAAATGATAAGATTCTCTGACTG
AAATTGCATTTACATTGAAATATGAGGAATGCAAGTCATATCTGAAACCAGATTTCATG
CCATTTTGTTGTCACAGAATCTTTGTTCTCTAAAATATGTTACATCATTAGCTTGAATTCA
AAAGTTCCTTTGCAAATATTTTGTTGTAATGACTGAGGAAGTATCAACTGAACTTGGGA
TATCAACTTCTGGTTTTCTTTTCTGTTTAAATATGTTATTGCTCATACTGGAATAAAAATC

TTCTTTTGACTGCATTATTGGATAATCCCTTTGGTATTTTCCTCTAATGATGCAGAGAAGG
AAAAAAGGAATAGCAATAACCACTGATATTCCAATTGCTATTAATGCTTCTGTTGCAGC
TAATAAAGTAGTTGTATCATGTAATTTTAAAGATAATGTCAATGATTTTGTTGACCCAAT
ATCATTGTGTGCTTCACAGCTGAAATTCCCAGTATCTTCAGAACTGATGTTTGTGAACTT
TAGCAGCTTCCCGGGCTGAACTGGAAGGTATTCATTTTCTTTATACCACGTGTAGTTTAT
AACAGGAGGATTGCTGCTTTTATAGTTGCAGGTTAGATGCATGTCTTTTCCTTCTCTTAA
ATTTATGGGATGGTGTGTTATTTTAACTTCTTTAGGTGGATACATAACATCTATCTCGAAT
TTCGGGGAATATGCCGATCCAACTGTGTTATCAGCTTTACACCTGTAAGTACCACTGTG
ACTGGTCTTAATTTCTTTAATGAATAAATACTTTTCAGAATTGTCACTCTTCTTTTTGTTA
ATGTACCAGGCATATGTTGTCACTTCTGGGTTACTCCTGTTAACTTTGCAGGATAAGTTC
ATAGAGTCACCTTCCATAATATCCATGGGAGTAATATGAGTAATTTTTACATCTTTTGGT
GCAAATAAAACATTCAGGATAACTGCAGGAGCACTTTCTATGACAGAATCAACTACAG
CAAAACATGCATACCGCCCAGCATGATTCTCTTTAATGCGGTCAAAGCGCAACTCTCTG
TCTTCACCAACTATTTCCCAAGACTGTCTACTACGTTTACGCCATTGATATTCTGTGATTT
TAGCATCACTGTTATTTGTGGAACAAATTAGTTTTACTTTGTCTCCTTCTTTAATAGTCCC
TTCTGGTTGTACGGTTATGGTTATTACATTTGTGGCACCTATGTGGAAGATCAGAGGAAG
TAGCAGAATGAATTCCATGTTAGTATGTTTCATTTTTTAAATTACAGCAGAAAAGCCTTT
ATCTTTGTAAGCAATTTCTTTCCAAACCAAATCAGCAACTCTGCATTCCAAAAGGAAAT
ACTTATTGCAACTGAAACAATACAGCACTTCCACTTTTTCCTTTGGCACTCCAGGAGCTC
TGTGTCCATGCCTTTAAATGTGCTTGAATACATTCTCATTGGGCACACACAAAAGGAAG
TCA

>MBL2_TRINITY_DN1058_c0_g2_i2
GTTTTATTTAAGTATGCACAACCAACTGAATTACATACAGTCATTTTTCATATGATGTGT
ATAGGTATGCCTCATTCCACTCTACTTACAGAAAACAAAAATAAAGACAACTTGGCAA
TTGTAAAGTTTAGAATCAATTTACTTTAAAGTACAAAATATGTAATGACTTGTACAAAC
AGATGCTATTCTCTTATAAAAATAATGGCAGTGCTGAATATATTTTTGTATCTCTTTACA
GGAATGGGCATCACTTATTACTCTATATGATTTGTTCATAATGTAACTGTTAGGATTATG
TCTTTTTGTATTTTGAGTTAAAACAAAAACCCATAATTAAAAGATCAATTGTTCAAATTC
TGAACTCTTTTCATTAATTCAAGTAATACACTGATATGACATTATTTCACAAAAGCAGC
ATAACTATATGAAATGCATATATTCCACTAGTATAAAACAAACTTAGAAACATCCTAGA
TATTCCTCACAGATACTTTGCCAAGCAGTTCCCCAAAGTATATACAGTTATACAACAAC
CTACAAACTGTTATATATTATTGTTTATTTGAAATCATTCCAACAGATCAGGCTTACCTT
ATATACTGTGCGGTAATTGTTTTTTTAACATGTTAGTCATTATATCTGGTTTGTGAGGGA
AGAATACTACCAAAATAAATACTTTGTTATTCTCTGTCCTAATGTTTGTTACTCACTGCA
GGCTTCATTTGTGAAAGCAACCAAAGCCCAGTAAGTATACCATTTAATGTAAAAAAAA
AAGAGTTGTGTGTCCCACTATGACAATAATTCACTGGCAATCATTCTGCATCCTCACAT
GAAAAAAGATAATCCTAAAAGCTGGGTCATTGCCAATCTTGCATCATTTCATTGTTAAT
GTTTCTGTTTTTAATGTCCTTAATATCAAATATTCCTTCACAGCTCATCACAGGTATTAAA
TTCTTCAATATTATATTTAAAGAACAAAAACATAAAAAATGAAAGTACGCACCATAAG
GTTATGAAGAAAGGATGCTGAGCTAAGTTACTTAAATCATAAAACTATGATTGTTACCC
TTAAATTAGAGACAGGAATATTGTAACATTACTTAGCTGATGAAGAACACATTAAACTG
TAATTAAAGTTTGGAGCTTACTCAACGCATAGTGATAAAAAGAATCCACTGATAAAAC
AAATGGTTTCCATGCTAAGGCAAAATCAATGAAATTACTGTGAGACTTCTGAAAAATCA
TTTACAACTCACAGATAACAAGACAATGATTTGAACAGGGCTTGTCATTCCACTTTCCT
GTGGTTTGTATTGATGCACAATCTTCAGCATTATTATGATTATTTGGTTCACCAGAATTCC
ATTTTGAATATTTTATTGGATTCCCATCTAAATATACAAATTTGCCTTCTGTTTTTTCATCT
GAAACTGCAATCCATGCATCTTTCTTGACCCTCTTTACAATTTCTTCTATAGCTTTGTTTT
CAGCTTCTGTCATTGGAACAGCCATTCGAGAACCAACACGTGCACATTCTGACTTGGCA
GTTGAAAAGTCAGACATTGTTTCTCCAGTAAAAAATGTTTTGTCTCCAACTTTCCAGCTT
TTACCTAGGAACTGCAAAACTGGAATTCCTGGACTGCCAGTTGCACCTTTCATCCCTTCA

TTTCCCTTTGGGCCTGTTGACCCAGTAGCTCCCTTTTGTGCAGCAGGGCCTGCTTTTCCTG
GAGGACCTTGAGCCCCCTTTAATCCTTGTCCTGGTGGGCCTGTATTTCCTTTGTCACCCTT
AAGTCCATCTCTCCCTGGTAATCCAGGTATTCCTGGTATTCCCTGAACTGCAATACATGT
TGGCTGGTTTGCAGGAGATTGACAAAAAGCTGCCATTAGCATTATCACAGCTGTGAAAA
TACTGAATGGGTGAAGAAGCATGATTTGAGCAGTAAACTAGGAGCCTTCTCAGTGAAG
AACTTGTCTAAACTGCACACATGCGAAGAGTCCACATCAGGAATCAAACTCTGGGACT
TCCAGGGGATGGTCTGAGCCAGCAGCAGTGCTTTCACACAGCTCTTCCTCCATAAAAAA
GAATTCTCAAGCCAAAGGGA
