# Supplementary material for: Potential Involvement of the South American Lungfish Intelectin-2 in Innate-Associated Immune Modulation
Source: Int J Mol Sci. 2024 Apr 27;25(9):4798. doi: 10.3390/ijms25094798 (PMC11084424; doi:10.3390/ijms25094798)
Supplement: Supplementary file 1 [file ijms-25-04798-s001.zip › Supplementary Table S1.pdf]

Supplementary Table S1. Protein-ligand interaction of *Lp*ITLN2-B in the Calcium-binding site by GOLD.

Carbohydrate	H-bond (H-distance)	Hydrophobic interaction
Arabinose	His289 (3.03 Å)	Tyr269; Glu270; Asn286; Glu288; Gln300
Fructose	His289 (2.86 Å)	Glu270; Asn286; Glu288; Gln300
Fucose	His289 (3.03 Å); Asn286 (2.92 Å)	Tyr269; Glu270; Glu288; Gln300
Galactose	His289 (2.74 Å); Asn286 (2.88 Å)	Glu270; Glu288; Gln300; Trp314; Trp323
Glucose	His289 (2.76 Å); Asn286 (2.83 Å)	Glu270; Glu288; Gln300; Trp323; Trp314
N-acetylglucosamine	Asn286 (2.53 Å); Asn268 (2.60 Å)	Glu270; Glu288; His289; Thr305; Trp 314; Trp323
Lactose	His289 (2.78 Å)	Asn268; Tyr270; Glu270; Cys285; Asn286; Glu288; Gln300; Trp314; Trp323
Lipopolysaccharide	His289 (3.31 Å); Glu288 (2.28 Å); Asn286 (2.53 Å)	Tyr269; Glu270; Gln300; Val304; Ala313; Trp314; Trp323
Maltose	Asn286 (2.99 Å; 2.53 Å) Glu288 (2.28 Å); Tyr269 (2.59 Å)	Asn268; Glu270; Cys285; Gln300; Trp314; Trp323
Mannose	His289 (2.76 Å) Asn286 (3.01 Å)	Glu270; Glu288; Gln300; Trp314; Trp323
Poly(I:C)	Glu270 (2.93 Å); Asn286 (2.53 Å); Glu288 (2.42 Å); His289 (2.77 Å);	Gln300; Trp323
Rhamnose	His289 (3.03 Å); Asn286 (2.92 Å)	Tyr269; Glu270; Glu288; Gln300
Ribose	-	Glu270; Asn286; Glu288; His289; Gln300; Thr305; Trp314
Sucrose	His289 (2.90 Å); Glu288 (2.28 Å); Asn286 (3.01 Å; 2.53 Å)	Tyr269; Glu270; Gln300; Trp314; Trp323
