# Supplementary material for: Potential Involvement of the South American Lungfish Intelectin-2 in Innate-Associated Immune Modulation
Source: Int J Mol Sci. 2024 Apr 27;25(9):4798. doi: 10.3390/ijms25094798 (PMC11084424; doi:10.3390/ijms25094798)
Supplement: Supplementary file 1 [file ijms-25-04798-s001.zip › Supplementary Table S2.pdf]

Supplementary Table S2. Protein-ligand interaction of *Lp*ITLN2-B in the Calcium-binding site by DockThor.

Carbohydrate	H-bond (H-distance)	Hydrophobic interaction
Arabinose	Asn286 (2.25 Å)	Tyr269; Glu270; Glu288; Gln300; Trp314; Trp323
Fructose	Glu270 (2.91 Å) Glu288 (2.66 Å; 2.57 Å); His289 (2.72 Å)	Asn286; Gln300; Trp314
Fucose	Glu270 (2.73 Å); Glu288 (2.47 Å; 2.53 Å); His289 (2.54 Å)	Asn286; Gln300; Trp314; Trp314
Galactose	Glu270 (2.47 Å); Glu288 (2.52 Å); His289 (2.79 Å)	Tyr269; Asn286; Gln300; Trp314
Glucose	Glu270 (2.62 Å); Glu288 (2.84 Å; 2.65 Å); His289 (2.99 Å); Trp314 (2.75 Å)	Asn286; Gln300
N-acetylglucosamine	Asn286 (2.53 Å); Asn268 (2.60 Å)	Glu270; Glu288; His289; Thr305; Trp 314; Trp323
Lactose	Asn286 (2.97 Å); His289 (2.28 Å); Gln300 (2.35 Å); Trp314 (3.35 Å);	Trp323
Lipopolysaccharide	Asn115 (3.18 Å); Ser310 (3.43 Å); Ala313 (3.44 Å)	Ile116; Lys119; Glu288; Gln300; Gly301; Val304; Thr305; Cys306; Gly307; Phe309; Gly311; Phe312; Trp314;
Maltose	Asn286 (2.88 Å); Gln300 (2.70 Å)	Trp314; Trp323
Mannose	Glu270 (2.79 Å; 2.71 Å); Glu288 (2.58 Å; 2.54 Å); His289 (2.75 Å)	Tyr269; Asn286; Gln300; Trp314; Trp323;
Poly(I:C)	Gln300 (2.50 Å); Trp314 (2.26 Å; 2.88 Å)	Asn286; Gly301; Trp323
Rhamnose	Asn286 (2.92 Å; 2.95 Å); Glu288 (2.53 Å); His289 (2.84 Å)	Gln300; Trp314; Trp323
Ribose	Glu270 (2.62 Å); Asn286 (2.53 Å; 2.60 Å); Glu288 (2.63 Å; 2.63 Å)	Gln300; Trp314
Sucrose	Glu288 (2.78 Å); Gln300 (2.97)	Glu270; Asn286; Trp314
